# Recent trends in nitrogen cycle and eco-efficient nitrogen management strategies in aerobic rice system

**DOI:** 10.3389/fpls.2022.960641

**Published:** 2022-08-25

**Authors:** Muhammad Shahbaz Farooq, Xiukang Wang, Muhammad Uzair, Hira Fatima, Sajid Fiaz, Zubaira Maqbool, Obaid Ur Rehman, Muhammad Yousuf, Muhammad Ramzan Khan

**Affiliations:** ^1^Institute of Environment and Sustainable Development in Agriculture, Chinese Academy of Agricultural Sciences, Beijing, China; ^2^National Institute for Genomics and Advanced Biotechnology, Islamabad, Pakistan; ^3^College of Life Sciences, Yan’an University, Yan’an, China; ^4^Department of Agronomy, University of Agriculture, Faisalabad, Pakistan; ^5^Department of Plant Breeding and Genetics, The University of Haripur, Haripur, Pakistan; ^6^Institute of Soil Science, Pir Mehr Ali Shah Arid Agriculture University, Rawalpindi, Pakistan; ^7^Pakistan Agricultural Research Council, Islamabad, Pakistan

**Keywords:** aerobic rice, nitrogen cycle, eco-efficiency, microbial activities, current trends, agronomic practices

## Abstract

Rice (*Oryza sativa* L.) is considered as a staple food for more than half of the global population, and sustaining productivity under a scarcity of resources is challenging to meet the future food demands of the inflating global population. The aerobic rice system can be considered as a transformational replacement for traditional rice, but the widespread adaptation of this innovative approach has been challenged due to higher losses of nitrogen (N) and reduced N-use efficiency (NUE). For normal growth and developmental processes in crop plants, N is required in higher amounts. N is a mineral nutrient and an important constituent of amino acids, nucleic acids, and many photosynthetic metabolites, and hence is essential for normal plant growth and metabolism. Excessive application of N fertilizers improves aerobic rice growth and yield, but compromises economic and environmental sustainability. Irregular and uncontrolled use of N fertilizers have elevated several environmental issues linked to higher N losses in the form of nitrous oxide (N_2_O), ammonia (NH_3_), and nitrate (NO_3_^–^), thereby threatening environmental sustainability due to higher warming potential, ozone depletion capacities, and abilities to eutrophicate the water resources. Hence, enhancing NUE in aerobic rice has become an urgent need for the development of a sustainable production system. This article was designed to investigate the major challenge of low NUE and evaluate recent advances in pathways of the N cycle under the aerobic rice system, and thereby suggest the agronomic management approaches to improve NUE. The major objective of this review is about optimizing the application of N inputs while sustaining rice productivity and ensuring environmental safety. This review elaborates that different soil conditions significantly shift the N dynamics via changes in major pathways of the N cycle and comprehensively reviews the facts why N losses are high under the aerobic rice system, which factors hinder in attaining high NUE, and how it can become an eco-efficient production system through agronomic managements. Moreover, it explores the interactive mechanisms of how proper management of N cycle pathways can be accomplished via optimized N fertilizer amendments. Meanwhile, this study suggests several agricultural and agronomic approaches, such as site-specific N management, integrated nutrient management (INM), and incorporation of N fertilizers with enhanced use efficiency that may interactively improve the NUE and thereby plant N uptake in the aerobic rice system. Additionally, resource conservation practices, such as plant residue management, green manuring, improved genetic breeding, and precision farming, are essential to enhance NUE. Deep insights into the recent advances in the pathways of the N cycle under the aerobic rice system necessarily suggest the incorporation of the suggested agronomic adjustments to reduce N losses and enhance NUE while sustaining rice productivity and environmental safety. Future research on N dynamics is encouraged under the aerobic rice system focusing on the interactive evaluation of shifts among activities and diversity in microbial communities, NUE, and plant demands while applying N management measures, which is necessary for its widespread adaptation in face of the projected climate change and scarcity of resources.

## Introduction

### General background

Over explosion in industrial growth, economic developments, and rapidly growing population have blown up the demand for food and challenged future food security (Fahad et al., 2017; Fukase and Martin, 2017). Rice (*Oryza sativa* L.) is cultivated widely across the globe, except in Antarctica due to the icy conditions throughout the year. About 90% of the global rice is cultivated in Asia, the topmost continent for rice cultivation (Wassmann et al., 2009; Rose et al., 2014), where China is the largest grower and producer (Tang et al., 2014) constituting 30% of global rice production (Zhang et al., 2015). Rice is a thermosensitive crop and a major cereal crop globally after wheat. It is cultivated in nearly 95 countries where it is a staple food and fulfills the dietary requirements of more than half of the global population (Ainsworth, 2008; Wassmann et al., 2009; Paterson and Lima, 2010; Liu et al., 2013).

It has been indicated that global rice production should necessarily increase by approximately 20% by 2030 and 30% by 2050 at the rate of 0.6–1% increase annually to meet the demands of the growing population projected to result from rapid population growth, and economic and industrial developments (Peng et al., 2009; Shew et al., 2019). Investigating various strategies to increase grain yield in rice has been a key research focus of agronomists for many years (Shew et al., 2019; Mongiano et al., 2020; Zhao et al., 2020; Xu et al., 2021). During recent decades, most of the rice-growing regions experienced an obvious warming trend across the globe, and this trend is projected to change the duration of rice growth and undermine the grain yield (Liu et al., 2013; Raza et al., 2019; Fatima et al., 2020). A reduction in the grain yield has been reported in rice in the range of 6 and 10% with every 1°C increase in temperature (Stuecker et al., 2018; Wei et al., 2021). Meanwhile, climate change decreased the grain yield of wheat and maize by 3.8 and 5.5% since the 1980s, respectively (Lobell et al., 2011; Zhao et al., 2017).

Since the 1980s, the growth duration for rice across the globe has been extended, and an increase in yield has also been observed (Liu et al., 2012; Tao et al., 2013) due to improved management practices and developments in breeding programs, which helped in the production of new crop varieties (Xiao et al., 2008; Chen et al., 2009; Hall and Richards, 2013; Driedonks et al., 2016). An increase in the potential rice grain yield by about 30% has been observed through the development of semi-dwarf rice varieties, and a further 15–20% increase was reported through the utilization of heterosis programs (Chen et al., 2009; Zhang et al., 2009). It has been suggested that the varietal adjustments could help in stabilizing growth duration, such as the length of the pre-flowering phase and extent of the grain-filling period, which ultimately increases the grain yield (Liu et al., 2010; Sun et al., 2018). The varietal developments in summer maize contributed greatly to increased grain yield from 42.6 to 44.3% during the 1950s to 1970s, from 34.4 to 47.2% during the 1970s to 1990s, and from 21 to 37.6% during the 1990s to 2000s (Chen et al., 2012; Wang et al., 2021b; Thakur et al., 2022). Additionally, it has also been indicated that changes in irrigation measures and sowing dates could make great contributions to the increase in grain yield, particularly among cereals (Chen et al., 2012). Due to the deep involvement and tangled behavior of soil nutrient properties, climatic conditions, management measures, and crop varieties, more often, it is very difficult to determine the peremptory variable for change in grain yield (Hirzel and Matus, 2013; Fahad et al., 2017; EL Sabagh et al., 2021). A detailed and deep understanding of how to differentiate the impacts of the influencing variables on the grain yield may provide worthy knowledge for insights into the development of sustainable rice systems in the face of future projected climate change and scarcity of resources (Mahdu and Mills, 2019; Saud et al., 2022). Therefore, the objectives of this study were to conduct thorough investigations and provide insights into the recent advances in major pathways of the N cycle under the aerobic rice systems and suggest agronomic management measures to interactively increase the N uptake by plants, thus reducing N losses and environmental hazards, and improving the grain yield and widespread adaptation of aerobic rice system.

Nitrogen is an essential mineral nutrient required in higher amounts for optimum plant growth and developmental processes. It is a necessary element of amino acids, nucleic acids, and many photosynthetic metabolites, which makes it critical for normal growth and metabolism (Koch et al., 2019; de Bang et al., 2021). It has already been predicted that N fertilizers could fulfill the overall 48% requirements of the inflating global population. But, uncontrolled and irregular amendment of N fertilizers has elevated the environmental sustainability issues linked with higher N losses either through leaching or volatilization, thereby creating environmental pollution due to greenhouse effects and contributing to ozone depletion (Raza et al., 2018). Irregular agricultural management practices share major contributions toward anthropogenic emissions across the globe, which necessarily demand the efficient utilization of synthetic N fertilizers that can only be achieved through appropriate agronomic management approaches (Smith and Siciliano, 2015; Aryal et al., 2021). Increasing NUE is an essential soil–plant interactive trait that has been under consideration for many years because it explains the overall grain production and also estimates the economic return. Generally, NUE is defined as the sum of N uptake efficiency and N utilization efficiency, thus explaining that increase in NUE boosts the crop yield, biomass, and quality (Congreves et al., 2021; Manschadi and Soltani, 2021; Riache et al., 2022).

Nitrogen is one of the major mineral nutrients that share greater roles in photosynthetic pathways, hormonal and enzymatic processes, and other proteomic changes, which are necessary for the growth and developmental stages of plants (Balestrini et al., 2020; Sandhu et al., 2021). Inefficient, excessive, and uncontrolled incorporation of N fertilizers subsequently produces enhanced crop yield, however, by compromising the economic and atmospheric concerns. To make the future inflating population more food secure, it is necessary to make the food production systems more sustainable with no harm to atmospheric sustainability (Farooq et al., 2022b), which can only be possible through an urgent upgradation to increase NUE in the agriculture sector (Farooq et al., 2022a). Poor plant population, traditional incorporation methods, and uncontrolled excessive application usually increase the N losses, ultimately posing environmental threats (Mahmud et al., 2021). These losses eventually raise an urgent need for the incorporation of improved agronomic management measures to increase food production, improve economic benefits, and reduce environmental hazards.

The first section of this review article discusses the importance of global climate change status and challenges to rice production, and thereby reviews the importance of replacing the traditional rice system with an aerobic rice system. Later, it narrows down toward the discussion about shifts in N cycle pathways (ammonification, nitrification, denitrification, leaching, and volatilization) under the transformed aerobic rice system. The second section of the article specifies the investigation of challenges due to high N losses associated with the aerobic rice system and how an aerobic rice system can be an important and eco-efficient rice production system under changing climatic conditions. Moreover, it also reviews the major factors impacting the N management and NUE. The final section expands toward the evaluation of several agricultural and agronomic management measures aiming to reduce the high N losses, while increasing the NUE and grain yield under an aerobic rice system with limited threats to environmental safety.

### Climate change and challenges to rice production

Climate change is a reality and subsequently impacts the population, ecosystems, and livelihoods (Hornsey et al., 2016; Stecula and Merkley, 2019). Due to the widespread impact of climate change, it constitutes major developmental challenges for the global community, particularly for the poor and natural resource-dependent populations in developing and underdeveloped countries (Devkota and Paija, 2020). The global phenomenon of climate change impacts the communities unevenly and irregularly both within and between countries (Ali et al., 2017). Within a country, more often, climate change impacts the poor, politically marginalized and disfranchised populations, and the least equipped who are at the topmost to experience the negative impacts and less likely to diversify their livelihoods (Mannke, 2011; Islam and Winkel, 2017).

Due to irregularities and unevenness in the variabilities of climatic components, the mean earth surface temperature has been increasing since the beginning of the 19th century. Based on the temperature fluctuations projected over the last few decades, the global atmospheric temperature has risen by 1.09°C since the 1950s based on the facts revealed in the Intergovernmental Panel on Climate Change (IPCC) annual report 6 (AR6) working group I (WGI) report, and most of this temperature change was noticed in the last 30 years of the 19th century (IPCC, 2021). Three major factors that are known to contribute to climate change are natural variations, human-induced factors, such as greenhouse gas emissions (GHGs), and land-use changes. Human-based interventions, such as fossil burning and industrial developments, have raised the atmospheric carbon dioxide (CO_2_) concentrations from 284 to 410 ppm from 1832 to 2013 (Hoffman et al., 2014), respectively, consequently causing global warming and changes in the mean atmospheric temperature. Future climate change projections have revealed that changes in temperature; precipitation intensity, frequency, and patterns; and extreme climatic disasters would be more intense in the coming years (Tabari, 2020). Moreover, it is expected that patterns for global warming will show unevenness across the globe due to which arid and oceanic regions will be facing more threats under uncertain extreme climatic events (Solomon et al., 2007; Dastagir, 2015). Meanwhile, it has also been anticipated that the earth surface temperature will increase more slowly than that revealed by the projections of climate models due to more absorption of CO_2_ by oceans (Balmaseda et al., 2013, 2015). Hence, the inefficiency of the climatic models to project the accurate hydrological cycle over the tropical regions create several uncertainties in patterns, frequency, and intensity of precipitation (Lorenz et al., 2012).

**FIGURE 1:**
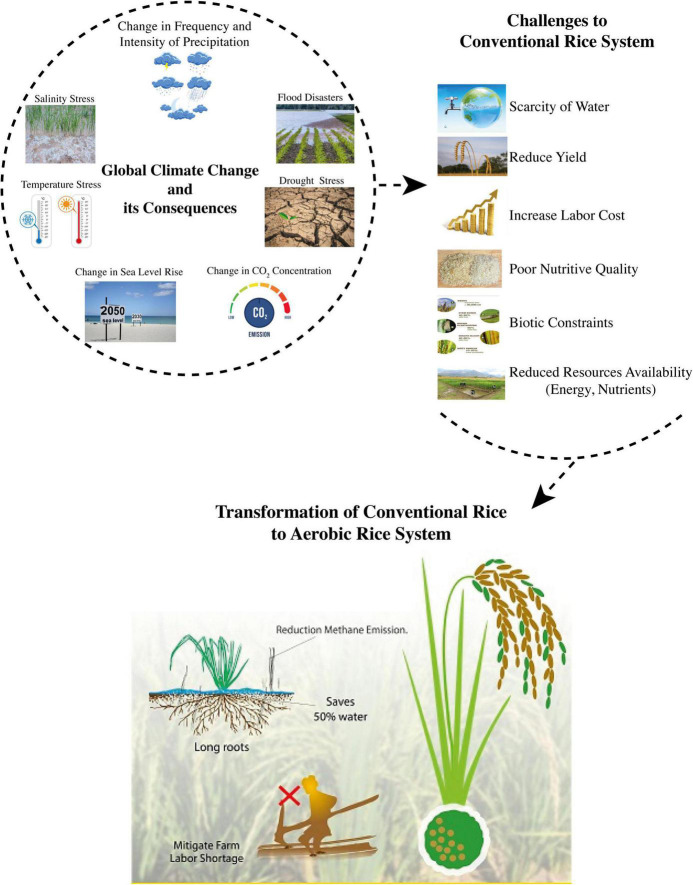
Global climate change and challenges to rice production (Sources: Horie, 2019; Hu et al., 2019; Elbasiouny and Elbehiry, 2020; Song et al., 2022).

Climate is considered as one of the environmental components for the success or failure of a crop plant. It assists and plays an important role in short- and long-term crop planning, particularly under changed climate conditions or climate disaster events (Figure 1). Past climate changes that occurred in recent decades caused several vulnerabilities to food production systems globally (Lobell and Field, 2007), as well as regionally (Horie, 2019; Luo et al., 2022). Over the next 30–50 years, the mean surface temperature is predicted to rise by 2–5°C due to global warming caused by climate change (IPCC, 2021). Stress due to high temperatures and other temperature-related extreme events are expected to become more frequent and intense imposing negative impacts on crop growth duration and yield (Li et al., 2012; Wang and Peng, 2017). During the past 30 years, the mean surface temperature has increased due to uneven and uncontrolled human-based activities, such as deforestation and lack of afforestation, industrial developments, and unsteady burning of fossils, which boosted GHG emissions (Onoja et al., 2011). Extreme climatic disasters are expected to adversely impact the crop growth duration, overall growth, and development (Kang and Eltahir, 2018).

Global rice production has been threatened due to uneven and unsteady climatic variabilities, particularly in Asia. Climate change and rice production are closely interlinked with each other, as the flooded rice system contributes to GHG emissions and causes global warming, thus climate change ultimately impacts the rice cultivation system (Figure 1). According to the estimates of the Food and Agriculture Organization (FAO), rice production is necessarily required to increase by 40% by the end of the 2030s to meet the food security aims under the scarcity of resources (FAO, 2016). This increase in food, if ensured with the least occupation of eco-efficient measures, may aggravate the issues of environmental pollution. China and India are the topmost populated countries, sharing 20 and 28.5% of the total global rice cultivated area, respectively (Hwalla et al., 2016). Most of the rice across the globe is cultivated under a flooded system, such as 90% of rice cultivation in China and 46% in India is produced under well-puddled and well-saturated soil conditions (Pathak et al., 2005; Oo et al., 2018; Kumar et al., 2021). Rice cultivated under conventional flooded conditions is considered to be one of the main factors of GHG emissions (Oelbermann, 2014). Therefore, various transformations have been introduced for rice production, considering the future scarcity of resources with less methane (CH_4_) emissions but serve several issues of environmental hazards due to high N losses via volatilization and leaching.

The emission of GHGs from either flooded or modified rice soil is majorly dependent on soil organic matter (OM) content, land use, cropping intensity and pattern, irrigation management, microbial abundance and functioning, soil chemical and physical properties, and environmental variables (Oo et al., 2018). Moreover, the emission rates of GHGs from rice soil are also dependent on coupled soil, crop, and fertilizer management practices. Commonly, the GHG emission rate is higher under the conventional rice system, but the modification of the conventional system to fulfill food security objectives and make rice production more sustainable under limited availability of natural resources causes high N losses due to intermingled nitrification–denitrification processes (Huang and Gerber, 2015; Islam et al., 2020). Therefore, for fulfilling the future food requirements under the scarcity of resources and reducing GHG emissions, modification of the conventional rice system or replacement with an aerobic rice system is necessary. However, due to high N losses and environmental pollution issues, widespread adaptation of aerobic rice necessarily requires investigation and evaluation of adjustments in the pathways of the N cycle and microbial abundance and functioning under the aerobic rice system through agronomic management approaches.

### Adopting the aerobic rice system

**FIGURE 2:**
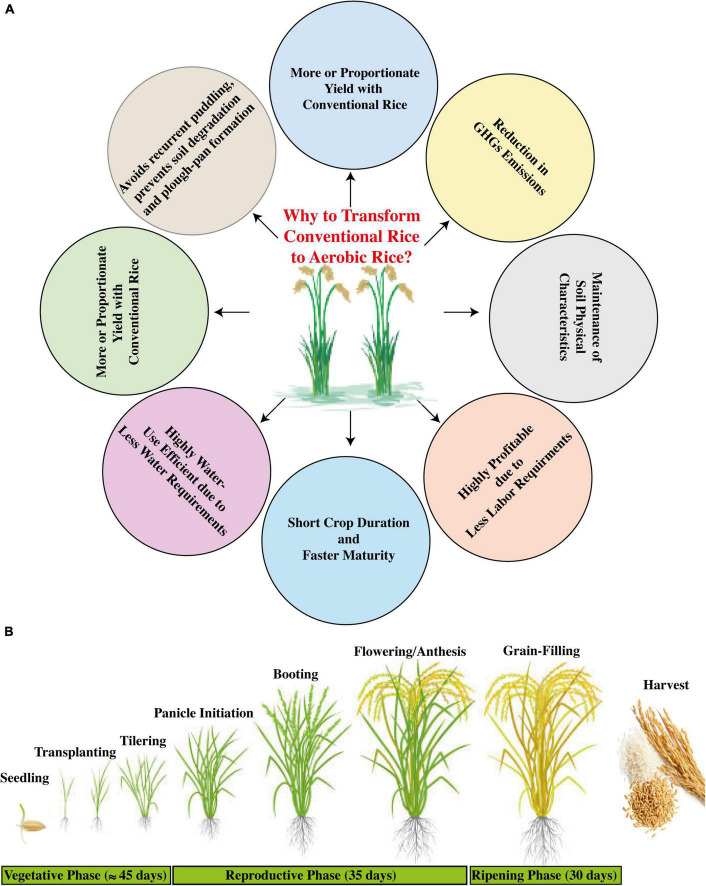
Transformation of conventional rice to aerobic rice system (Source: Joshi et al., 2018). **(A)** Benefits of aerobic rice system (Source: Silwal et al., 2020), and **(B)** elaboration of its growth phases (Source: Shah et al., 2014).

Rice is an important staple food crop in many countries around the globe. In Asia, the social and economic stability is mostly dependent on flooded rice production, and it is a key element for the livelihood and dietary requirements of more than two billion population worldwide (Bishwajit et al., 2013). Conventionally, the rice production system implicates submerged conditions, within the range of 5–10 cm deep standing water during the whole rice growth period. Globally, the flooded rice system utilizes about 30% and within Asia more than 45% of the total global freshwater (Kadiyala et al., 2012). The increasing scarcity of freshwater resources due to accelerated demand for water from diversified sectors has threatened conventional rice sustainability and is projected to be more vulnerable under projected climate change, which necessarily demands various developmental modifications of novel water-saving technologies without compromising environmental safety and public health (Rasul, 2016). Traditional rice cultivation is increasingly experiencing problems due to a shortage of resources, such as irrigational water, energy, and labor. Puddling is an irremissible approach for conventional rice systems; however, it results in the deterioration of soil structure, thus hurdling the land preparation for the succeeding crops with exceeded energy requirements in attaining optimized soil tilth (Khairul Alam et al., 2020). Therefore, such factors accentuate the dire need of bringing modifications to conventional systems through the incorporation of water-saving methods, such as cultivating aerobic rice, which can serve as an eco-efficient system by saving natural resources without affecting the grain yield (Figure 2).

Naturally, rice is a drought-sensitive crop, and a reduction in irrigation supply can cause a decline in yield (Ahmadikhah and Marufinia, 2016). Previous studies have developed several modified technologies to reduce water application in rice systems, such as raised bed cultivation (Bhuyan et al., 2012; Kadiyala et al., 2012), alternate wetting and drying system (AWD) (Howell et al., 2015), saturated soil culture (Singh et al., 2021), and system of rice intensification (SRI) (Chapagain et al., 2011). Some of these are not water-use efficient due to continuous requirements of puddling and soil saturation during crop growth, and therefore, considerable water input cannot be obtained. Aerobic rice cultivation is a water-saving technique where rice is grown under non-saturated and non-puddled conditions of soil (Joshi et al., 2018; Singh et al., 2021). This modified technology mainly targets the irrigated lowland area where irrigational water is not sufficient for rice cultivation, and is also adaptable in upland areas where supplemental irrigational water facilities are ensured (Belder et al., 2004; Thakur et al., 2015). During the early years of aerobic rice technology, grain yield of up to 6.5 t ha^–1^ with approximately 60% water saving was observed (Kadiyala et al., 2012; Nie et al., 2012; Sandhu et al., 2019), depicting that aerobic rice cultivation can be a sustainable rice production system on a wide scale under limited natural resources and climate change.

## Pathways of N cycle in rice-based systems

### Eco-efficient rice system

Natural resources required in agriculture, including water, energy, and land, are decreasing every passing day, and therefore a projected increase in crop output needs to be attained eco-efficiently. Eco-efficiency means ‘getting more crop outputs with minimized use of inputs in agricultural systems, in terms of quality and quantity without compromising environmental safety (Keating et al., 2010; Czyżewski et al., 2020). The concept of eco-efficiency was introduced to stabilize the crop yield of high-input cropping systems (flooded rice systems in lowland areas), and also to increase the overall net yield of low-input cropping systems (upland rice cultivation) through the use of different transformational modified technologies (Gołaś et al., 2020). The aerobic rice system can be termed as an eco-efficient cultivation approach because of no requirements for continuous flooding and puddling throughout the growing season (Kadiyala et al., 2012; Devkota et al., 2013). Due to the higher utilization of irrigation water in flooded rice, it can be designated as a water-intensive food commodity. Undergoing this, it is crucial to develop a resource-use efficient rice system to manage the increasing irrigational water stress and also to make it more sustainable under future projected climate change and limited availability of natural resources. The conventional rice system has already become vulnerable due to looming freshwater crises, and this situation is predicted to become more severe due to global warming, climate change events, GHG emissions, and the lowering of groundwater levels. Irregular pumping and uncontrolled wastage of water have already imposed serious threats to agricultural sustainability in many countries where groundwater shares a great role in agricultural farming (Qureshi, 2018). Therefore, these threats have paved the way for researchers to develop eco-efficient aerobic rice systems in the last few decades by interacting the features of drought-resistant upland rice varieties with lowland rice varieties exhibiting high-yielding traits (Belder et al., 2005; Matsuo and Mochizuki, 2009).

### Why increase in N-use efficiency is important?

**FIGURE 3:**
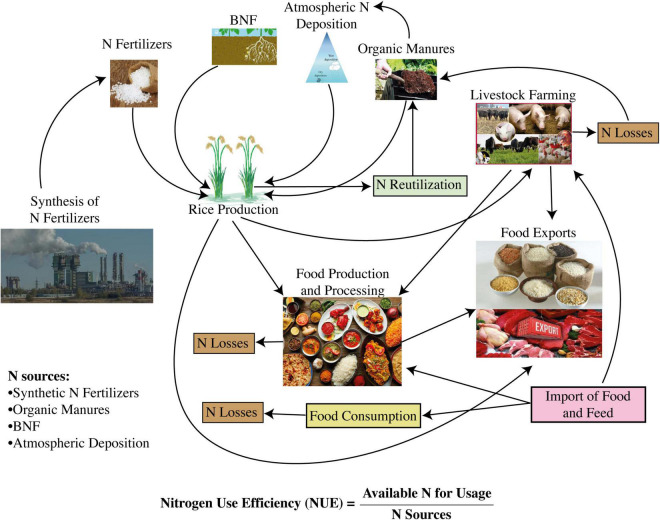
An embedded approach indicating nitrogen-use efficiency in food production and consumption system (Source: Lassaletta et al., 2016; Erisman et al., 2018).

Multiple chemical and biochemical pathways along with environmental variables impact the complex trait of NUE (Farooq et al., 2022a). Different agricultural major research wings define NUE differently, such as plant physiologists take into account various steps during N management, that is, N uptake, N assimilation, N allocation, and N remobilization, more obviously during leaf senescence (Sandhu et al., 2021). They also divide the NUE based on the different components of the N cycle, such as N uptake efficiency, N assimilation efficiency, N allocation efficiency, and N remobilization efficiency (Lee, 2021). Agronomists define the NUE as grain yield comparable to the total amount of available N from soil inclusive of N fertilizer application (Perchlik and Tegeder, 2017; The et al., 2021). Globally, all agricultural researchers agree that NUE is composed of two critical components, the N uptake efficiency and the N utilization efficiency, which can easily be monitored under controlled as well as field conditions (Lee, 2021). N uptake efficiency is defined as the overall capability to absorb, or uptake, N supplied from soil N pools, whereas N utilization efficiency is the indigenous capacity of the plant to utilize the absorbed N, and thereby facilitate assimilation and remobilization to produce end harvest products. Most appropriately, N uptake efficiency can be described as the optimum harmonization between the efficiencies of N assimilation and N remobilization (Dellero, 2020; Zhao et al., 2021). Meanwhile, N assimilation efficiency can be defined as the overall capacity for the assimilation of inorganic N to manufacture amino acids and various other essential N-containing molecules, while N remobilization efficiency can be ascribed based on the total amount of N being remobilized between the source and sink tissues (Dellero, 2020; Zhao et al., 2021). Each component of NUE is related to various complex traits, which include root morphological parameters, leaf senescence, N remobilization, and the capacities to extract available N from soil N pools (Lammerts van Bueren and Struik, 2017; Ierna and Mauromicale, 2019). Drivers that can improve NUE mainly include N uptake through different genetic modifications, improved N assimilation and remobilization, and their regulation. Moreover, a combination of conventional breeding approaches and agronomic practices is also crucial to increase NUE. Figure 3 represents the key components of NUE in a food production-consumption system. It also depicts the occurrence of N losses that hinder the improvement in NUE.

### N cycle in aerobic and anaerobic rice systems

Rice under fluctuating and challenging soil and environmental conditions has developed various N uptake systems. Plants preferably take up N from soil both in the forms of ammonium (NH_4_^+^) and nitrate (NO_3_^–^) (Chen et al., 2016, Chen J. et al., 2020). Under the flooded rice system, NH_4_^+^ is the major form due to submerged and anaerobic conditions, whereas NO_3_^–^ is the abundant form of N under upland aerobic conditions owing to intensive nitrification rates from applied organic and synthetic N fertilizers (Lee, 2021). Synthetic N fertilizers, such as urea which is the most commonly used synthetic N fertilizer, could be degraded into NH_4_^+^ and CO_2_ by urea-decomposing enzymes that are released by microbial communities in soil (Kojima et al., 2007; Zanin et al., 2014). However, it has been reported that urea can be imported from the atmosphere to root cells via biological pathways (Beier et al., 2019). Several inhibitors have been introduced for technical issues to enhance synthetic N fertilizer use and NUE (Nikolajsen et al., 2020).

**FIGURE 4:**
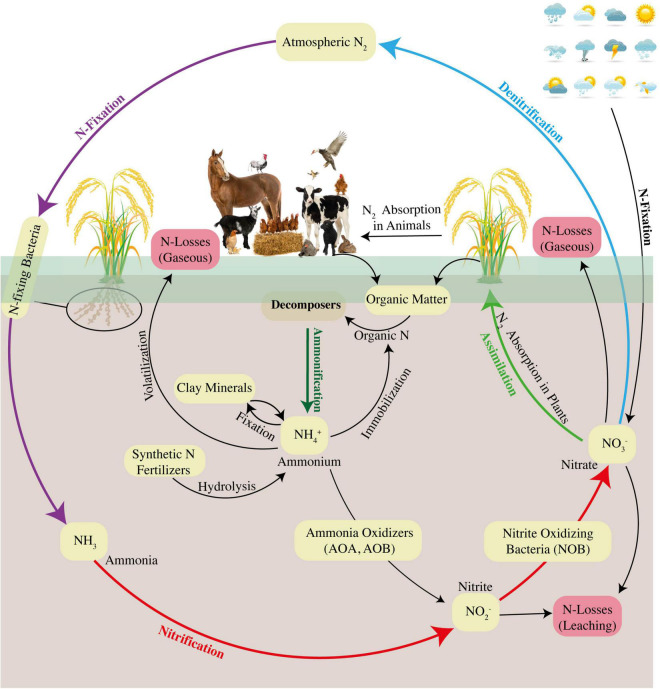
Understanding the N dynamics and overviewing the pathways of the N cycle under the aerobic rice system (Farooq et al., 2022a).

Many abiotic and biotic factors are involved in N cycling under both aerobic and anaerobic conditions, and the major pathways involved are ammonification, nitrification, denitrification, and fixation (Ishii et al., 2009, 2011; Oshiki et al., 2018). Researchers have started to focus on the contribution of different pathways to the N cycle and simultaneously explore the role of environmental factors and microbial functions to have better insights into the dynamics of the N cycle. However, the research has not been diverted to focus on insights into N dynamics under the aerobic rice system, which is urgently needed for better future adaptation and rice sustainability. In addition, recently, the pathways of microbial activities, fungal denitrification, and CH_4_ oxidation under rice flooded conditions have also been considered. Under conventional flooding and aerobic rice systems, the irrigation methods and management practices directly affect the availability, uptake, and loss of soil N (Amanullah and Hidayatullah, 2016; Amanullah et al., 2016). Under submerged soil and puddled conditions, there is a stagnant anaerobic condition in the field, which restricts the process of soil nitrification (Grzyb et al., 2021). Alternatively, under the aerobic rice system, the field is irrigated and dried through the AWD irrigation approach, where high water percolation could produce high nitrification rates, thus exposing the soil N to increased losses due to shuffled pathways of nitrification–denitrification (Xiong et al., 2010; Ju and Zhang, 2017). Hence, water management approaches during a change in the regime from conventional rice to aerobic rice impact the potential soil N availability, uptake, and assimilation. N cycle is composed of six key steps, namely, assimilation, ammonification, NH_4_^+^ oxidation, nitrite (NO_2_^–^) oxidation, denitrification, and fixation (Figure 4). The details of the most important steps are presented in the following section.

#### Assimilation and ammonification/mineralization

Assimilation is generally defined as the process that produces higher quantities of organic N which is further used to produce amino acids, proteins, and nucleic acids, whereas ammonification is the conversion of organic N into NH_4_^+^ via microbial communities (Silva et al., 2019; Zhang et al., 2019b; Grzyb et al., 2021). The NH_4_^+^ produced during ammonification is released into the ambient soil environment and thereby becomes available for either nitrification or assimilation (Figure 4). In the presence of both NH_4_^+^ and NO_3_^–^, rice plant preferably uptakes NH_4_^+^ more quickly than NO_3_^–^; hence, NH_4_^+^-based synthetic N fertilizers are usually applied in rice cultivation (Yi et al., 2019). Organic N fertilizers are also used in rice cultivation where organic N is converted to NH_4_^+^, and the process is termed as N mineralization (Silva et al., 2019). Comparatively, ammonification rates are higher in flooded rice soil than in aerobic rice system because of oxygen depletion in submerged conditions; however, this depleted oxygen condition restricts the process of oxidation of NH_4_^+^ to NO_3_^–^ (nitrification) and N immobilization (conversion of inorganic N to organic) principally through microbial assimilation activities, consequently causing accumulation of NH_4_^+^ in the soil (Mahmud et al., 2021).

#### Ammonium and nitrite oxidation (nitrification)

Microbial oxidation of NH_4_^+^ to NO_3_^–^ via nitrite (NO_2_^–^) is termed as nitrification (Barth et al., 2019) during which autotrophic nitrifiers can gain their energy for their routine activities (Figure 4). Meanwhile, some heterotrophic nitrifiers can process the conversion of inorganic and organic N to NO_3_^–^ or NO_2_^–^ with no acquired energy, and this process is termed as heterotrophic nitrification (Martikainen, 2022). Here, the discussion is only about autotrophic nitrification due to its large contribution to the N cycle relative to heterotrophic nitrification. Nitrification is a two-step process: first is the oxidation of ammonia during which the NH_4_^+^ ion is converted into NO_2_^–^, and second is the oxidation of nitrite during which the NO_2_^–^ ion is converted to NO_3_^–^. Ammonia oxidation can be processed by ammonia-oxidizing bacteria (AOB) (such as *Nitrosomonas* and *Nitrosospira* species of *Betaproteobacteria*, and *Nitrosococcus* species of *Gammaproteobacteria*) and ammonia-oxidizing archaea (AOA) (Hayatsu et al., 2017; Nacke et al., 2017; Lu et al., 2020; Lukumbuzya et al., 2020). Recent advances have found that AOA communities are more abundant than AOB, whereas AOB may have more functions during nitrification in the upland rice system (Banning et al., 2015; He et al., 2018; Liang et al., 2020). A Positive correlation between abundance and share in nitrification activities has been observed regarding AOB in flooded rice soil; therefore, it has been deduced that AOB might have more activities for ammonia oxidation over AOA (Meng et al., 2020). However, in the rhizosphere of a flooded rice system, the abundance of ammonia oxidizers significantly increased after the mixed application of synthetic N fertilizers, such as urea, and organic N fertilizers, such as biochar (Zhang et al., 2019a). So, understanding the role of microbial communities, particularly during nitrification under differential soil conditions and modified rice systems, is worthy to increase NUE.

In the flooded rice system, the diversity of AOB communities was less diverse as compared to other agricultural soils (Mukhtar et al., 2017). It has been observed that diversity in AOB communities was impacted by a change in rice varieties, and moreover flooded rice fields were characterized by an increased abundance of *Nitrospira* species (Ding et al., 2019), while clones related to the *amoA* of *Nitrosomonas* species were also detected in the paddy fields (Ke and Lu, 2012). Partially, this difference for *Nitrospira* and *Nitrosomonas* species may arise due to differential fertilization management, since *Nitrosomonas* species are found to be predominant in soil environments with high NH_4_^+^ concentration, whereas *Nitrospira* species are considered as major AOB in almost all agricultural soils (Peng et al., 2012). The second major step in the process of nitrification is the NO_2_^–^ oxidation which is primarily carried out by nitrite-oxidizing bacteria (NOB) belonging to the genera *Nitrospira, Nitrobacter, Nitrospina*, and *Nitrococcus* (Han et al., 2018). The literature regarding the community structure for NOB is still limited, but recently, large-scale 16S rRNA gene analyses showed the existence of a *Nitrospira-*like 16S rRNA sequence in flooded rice soil (Lopes et al., 2014) carrying out the potential responsibilities of *Nitrospira* species in NO_2_^–^ oxidation during flooded conditions. *Nitrospira* species were also observed in more diversity and abundance, especially, in agricultural grasslands and in soils with greater application of wastewater (Koch et al., 2015).

#### Denitrification

Denitrification is defined as the conversion of NO_3_^–^ and NO_2_^–^ to gaseous forms of N (Figure 4). In paddy rice soils, N oxides function as an alternative electron acceptor, whereas non-denitrifiers have the capacities to reduce gaseous forms of N, such as nitrous oxide (N_2_O), which can also serve as alternative electron acceptors (Bueno et al., 2015; Yoon et al., 2019). Denitrification can also be defined as the microbial reduction of NO_3_^–^ to NO_2_^–^ linked to electron transport phosphorylation, leading to the gaseous release of N either in the form of N_2_ or in form of N oxides (Sun et al., 2016; Wang H. et al., 2020). As per definition, the key to the denitrification process is based on the availability of N oxides, NO_3_^–^, and NO_2_^–^ that are being formed from the process of autotrophic nitrification pathway substrate, and ammonia which is being derived from NH_4_^+^ (Zhu et al., 2013). The incorporation of synthetic N fertilizers and soil OM mineralization are the major sources of NH_4_^+^ in the soil environment (Mahal et al., 2019; Farzadfar et al., 2021). The production of N_2_O from differential soil conditions primarily includes two biological reactions: the first step is the nitrification of NH_4_^+^ under aerobic soil conditions, and the second step is coupled nitrification/denitrification pathway that takes place under flooded soil conditions (Butterbach-Bahl et al., 2013; Zuo et al., 2022). The pathway projected for nitrifying microbes to discharge N_2_O during nitrification has been termed as nitrifier denitrification (Wang et al., 2022).

The pathway of denitrification is dominant once NO_3_^–^ is produced and optimized environmental conditions, such as high soluble carbon (C) contents, are ensured with optimized microbial functioning in the reduction of N oxides (Rajta et al., 2020). Respiratory denitrification or simply the denitrification process has been defined as a bacterial respiratory pathway; however, a clear differentiation is necessarily required between the denitrification pathway and nitrifier denitrification pathway, as the relative proportion of N_2_O output from these pathways is impacted by differences in the environmental conditions. A third pathway has also been found in varying soil conditions that is, more importantly, dominant in low soil pH conditions, which involves the chemical decomposition of NO_2_^–^ (Frostegård et al., 2021). Chemo-denitrification pathway, also known as the non-biological pathway, is linked with nitrification so intimately that it is often more difficult to ascertain whether N_2_O or nitric oxide (NO) is being manufactured via chemo-denitrification or nitrification process (Otte et al., 2019). Past research on denitrification was the outcome of the incompetency to mass balance total incorporated N inputs and N outputs in an agricultural system. The major proportion of the unaccounted N was intended to be lost as gaseous N, which consequently caused decreased agronomic NUE (AEN). Deep studies on global change processes have depicted side impacts of gaseous N losses due to nitrification-denitrification pathways (Guardia et al., 2021). Nowadays, N_2_O is considered as the most poisonous GHG with more leading influences on environmental and agricultural sustainability, because it is the most important component of global warming, nearly 320 times stronger than CO_2_, mainly because of the atmospheric lifetime of approximately 120 years (Naser et al., 2020). Additionally, as the levels of whether industrially or biologically fixed gaseous N utilized by plants increase, the overall production of N_2_O because of coupled nitrification-denitrification pathways will also boost and ultimately could have potential impacts on environmental sustainability.

Diverse groups of bacteria and archaea are involved in denitrification, and unlike nitrification, their functioning abilities are infrequently dispersed based on their taxonomy (Nishizawa et al., 2013; Grzyb et al., 2021). Culture-based analyses have discovered that the community abundance of denitrifying bacteria varies under differential soil conditions ranging from 103 to 105 per gram of soil sample (Ishii et al., 2011; Maeda et al., 2011; Wang et al., 2022). Stable isotope probing (SIP), functional single cell, and functional gene identification are some of the cross-functional strategies being applied for a more clear identification of microbial communities in rice soils (Yoshida et al., 2012). In cross-functional analysis, such as SIP, succinate has been utilized as an electron donor for denitrification because of its capacity to enhance the denitrification process rather than fermentation (Albina et al., 2019). Moreover, cross-functional analyses have also discovered that bacteria that belong to orders *Rhodocyclales* and *Burkholderiales* accompanying novel clades were demonstrated as the prevailing members of the ^13^C-succinate-assimilatory community under certain soil conditions (Saito et al., 2008; Wang Z. et al., 2020), while there was no correlation with archaea under respective soil conditions. On the other hand, 16S rRNA gene analysis comparatively demonstrated the microbial association and depicted that bacteria linked with the order *Burkholderiales*, particularly those belonging to the genus *Herbaspirillum* and *Rhodocyclales*, were in abundance in rice soils under denitrification-promoting soil conditions (Jang et al., 2019).

### Higher N losses under aerobic rice system

Currently, high N losses are the major threat to rice sustainability after water, due to their crucial roles in the growth and development of the plant (Jia et al., 2019). Aerobic rice preferably utilizes the NO_3_^–^ form of N, which may reduce the N losses due to ammonia volatilization (Mahmud et al., 2021). But, AWD irrigation management under an aerobic rice system causes the decomposition of OM which further leads to high N losses in the form of gaseous ammonia under flooding conditions, and when soil becomes dry, rapid nitrification-denitrification processes lead to increased N losses in the form of N_2_O (Sahrawat, 2010; Kadiyala et al., 2012). The abundantly available form of N for rice plants under an aerobic system is NO_3_^–^, which limits the loss of ammonia due to volatilization, which is in contrast to the abundant form of available N in the conventional rice system (Huang J. et al., 2017). Due to irrigation management through the AWD system in the aerobic rice systems, high ammonification through increased decomposition of OM occurs during dry soil conditions, which leads to N losses during flooded soil conditions (Virk et al., 2020). Thereby, soil again becomes dry which stimulates the ammonification process again subjecting toward nitrification-denitrification causing more gaseous N losses (LaHue et al., 2016; Cui et al., 2018). Apparent N recovery (ANR) and AEN are greatly impacted by irrigational, soil, and crop management, along with soil microbial activities which are dependent mainly on varying soil conditions (Fuhrmann et al., 2018; Shahane et al., 2019). This further ensures that the amendment to increase synthetic N fertilizers could produce sustained grain yield due to increased plant N uptake and biomass production but at the risk of environmental pollution due to high N losses, as the total amount of available N is beyond that required by plants (Maragatham et al., 2010; Zhou et al., 2017). Therefore, widespread adaptation of this transformed rice system requires the understanding and evaluation of eco-efficient N management measures for efficient use of N by plants and reducing its losses, while ensuring profitability and healthy environmental sustainability (Nie et al., 2012; Awan et al., 2014).

## Toward eco-efficient N management

### Gross soil N transformation pathways

Nitrogen is an essential component for proper plant growth, and its forms and amount are mainly checked by N production and consumption pathways in natural soil conditions (Galloway et al., 2008; Zhu et al., 2011; Zhang et al., 2016). If N is not efficiently conserved in different soil conditions, it will potentially cause high N losses and ultimately may induce negative impacts on human health due to threats to environmental sustainability (Zhang et al., 2013, 2018c; Ren et al., 2021). Therefore, quantification of simultaneous occurring pathways, i.e., N production and consumption, is essential in the identification of whether different soil conditions can efficiently retain N or require need further amendments. Understanding and interpreting fundamental pathways and characteristics of soil N transformation is crucial for better N management and reducing N losses under the aerobic rice system. Most of the previous studies of N transformation have been conducted using measurements of net N turnover rates; however, the recently designed technique of 15N pool dilution (Luxhøi et al., 2005) together with numerical models have been utilized in the measurements of gross N transformation rates (Müller et al., 2004). This measurement tool has essentially proved its importance in revealing the interactive relationships between N mineralization and N immobilization, N turnover and N losses, and N forms and their availability to plants (Zhengchao et al., 2013; Lama et al., 2020).

#### N mineralization

Ammonification is the process of N mineralization where the reduced form of organic N (NH_2_) is converted into gaseous NH_3_ or reduced inorganic form (NH_4_^+^) as the end product, as discussed in the previous sections (Farooq et al., 2022a). Many species of ammonia oxidizers are involved in this biochemical reaction, such as bacteria (AOB) and archaea (AOA), to obtain the required energy for their metabolism via oxidation of organic N to NH_4_^+^. Later, the NH_4_^+^ ion is readily available for the processes of assimilation and incorporation into proteins (amino acids) and other metabolic pathways. Moreover, it has also been described earlier that if the NH_4_^+^ N is not readily taken up by the microbial communities or excessively present than required in the metabolic processes, it will lead to high N losses to the ambient environment, soil, or water for plant uptake via nitrification (Jacoby et al., 2017). Under the presence of different forms of N in the soil, rice plant prefers NH_4_^+^ rather than NO_3_^–^ form of N for its uptake (Abbasi et al., 2017; Coskun et al., 2017; Yi et al., 2019; Chalk and Smith, 2021). Meanwhile, N evolved from organic sources is readily converted to NH_4_^+^ via biological N mineralization, which is also referred to as ammonification (Silva et al., 2019). N mineralization rate under aerobic rice systems is usually less than other cereals that show high oxygen depletion during flooded conditions. Summarizing the discussion of previous sections regarding N pathways, it can be deduced that the amendment of N-containing organic fertilizers along with certain synthetic N fertilizers in aerobic rice systems can prove to be an efficient approach to increase NUE (Awan et al., 2014; Huang et al., 2018).

#### Ammonium oxidation

The biological oxidation which involves the conversion of NH_4_^+^ to NO_3_^–^ via the intermediate production of NO_2_^–^ is termed as nitrification (Black et al., 2019; Stein, 2019). In rice soils, NH_4_^+^ is readily oxidized to NO_3_^–^, which subsequently accumulates in higher concentrations in the soil solution. Biological oxidation of NH_4_^+^ ions to NO_3_^–^ via NO_2_^–^ stipulates the translocation of N through the negatively charged soil particles, and hence, this oxidative conversion determines the actual N fate in the soil. Generally, nitrification in rice soil is mediated in two ways, as described in the previous section of N pathways, autotrophic nitrification during which the autotrophic nitrifiers acquire their required energy for their metabolic processes (Jung et al., 2011), and heterotrophic nitrification during which heterotrophic nitrifies convert the inorganic N (NH_4_^+^) and organic N to NO_3_^–^ via intermediate NO_2_^–^ without acquiring any of their required energy (Zhang et al., 2012; Hayatsu et al., 2021; Martikainen, 2022). The most likely form of N to translocate from soil solution to plant roots via mass flow is NO_3_^–^ rather than NH_4_^+^, and if not readily taken up by the plant, it will cause leach down during flooding or be lost via denitrification during dry soil conditions. Therefore, concluding the above discussion, fulfilling the aim to increase the overall NUE of the applied organic and inorganic N fertilizers in aerobic rice systems necessarily requires adaptive management measures to reduce the N losses (Norton and Ouyang, 2019).

#### N loss pathways

##### Ammonia volatilization

Strong N management should intend to acquire higher crop yield, by balancing the soil fertility status and reducing the environmental hazards (Bayu, 2020). Currently, the recent advanced studies about the N cycle and environmental hazards have enriched the knowledge to achieve better N management goals (Mahmud et al., 2021; Móring et al., 2021). However, due to specific natural and socio-economic features, limited N supply or over- and misuse of N fertilizers occur, where the former condition will cause reduced crop yield and the latter will lead to risks of environmental pollution. N loss pathways majorly involve ammonia volatilization, denitrification, leaching, and runoff, and integrated consideration of all these components during N management is necessary rather than focusing only on one component (Liu et al., 2020). Ammonia volatilization into the atmosphere majorly contributes to the production of secondary aerosols, which could be translocated from the production point and consequently may deposit in other ecosystems. Leaching of NO_3_^–^-N from rice soils is also a major source of pollution spread in groundwater reserves, where NO_3_^–^ entering the soil surface via leaching or runoff may stimulate eutrophication (Wick et al., 2012). N oxides emitted during nitrification or denitrification pathways are major GHGs that compromise the availability of a sustainable and healthy environment (Subbarao et al., 2013; Hu et al., 2017; Groenveld et al., 2020; Hassan et al., 2022). Various forms of reactive oxygen can pose impacts on environmental sustainability if immobilized or denitrified in previous N_2_ forms (Pan et al., 2022). Therefore, the quantification of different N loss pathways is worthy to minimize environmental hazards arising from irregular inputs of synthetic N fertilizers while sustaining the widespread future adaptation of the aerobic rice system (Farooq et al., 2022a).

Amendment of synthetic N fertilizers and organic manures in the agricultural sector is the largest source of NH_3_ emissions (Xu et al., 2015; Kang et al., 2016). Volatilization of NH_3_ normally occurs from the applied N fertilizers due to the transport of NH_3_ from the surface of an ammoniacal solution to the ambient atmosphere (Behera et al., 2013; Ju and Zhang, 2017). Fertilizer management practices, soil properties, environmental variables, and irrigation methodologies are the leading factors that impact the process of ammonia volatilization, plant N availability and N uptake, and overall NUE (Cameron et al., 2013). Among these, fertilization methods have major impacts on ammonia volatilization and emissions, where potential NH_3_ emissions could reach up to 64–68% of total amended N fertilizer (Drury et al., 2012; Woodley et al., 2020). Ammonia volatilization is usually reduced during irrigation or precipitation, as N fertilizer can move below the soil surface. Efficient irrigation depth varying from 5–75 mm could greatly reduce the volatilization of ammonia (Viero et al., 2015). Variation in soil pH impacts the comparative concentration of NH_4_^+^ and gaseous ammonia in the soil solution, where NH_3_ production and emission are elevated under high pH conditions (Rochette et al., 2013). Hence, calcareous soils with naturally higher pH can suffer from increased gaseous ammonia losses. As soil saturation increases, ammonia volatilization of synthetic N fertilizers can either be increased because of the faster hydrolysis process or decreased due to dilution in the soil solution. Moreover, high temperature encourages the hydrolysis process of synthetic N fertilizers and the rate of NH_3_ diffusion from soil solution to the ambient environment (Lei et al., 2017).

There could be many ways to reduce the NH_3_ emissions via fertilizer management, such as deep placement of synthetic N fertilizers, use of urease inhibitors, and modifying the fertilizer types (Li et al., 2015, Li Q. et al., 2017, Li et al., 2019), which could increase NUE due to decreased ammonia emissions by 40–83%. Another meta-analysis study has already shown that deep placement of N fertilizers prominently reduced NH_3_ losses by more than 60% (Huang et al., 2016; Yao et al., 2017) but at the cost of higher N_2_O emissions. On comparing the deep-band placement of N fertilizers with broadcasting, the former greatly reduced the NH_3_ losses by 69%, but a twofold increment in N_2_O emissions was observed (Yao et al., 2017). Nitrification inhibitors (NIs) can potentially be used commercially in rice soils to reduce N_2_O emissions and increase NUE (Ruser and Schulz, 2015; Huérfano et al., 2022); however, high NH_4_^+^ concentration persists in the soil for a prolonged duration due to the amendment in synthetic N fertilizers, thus increasing the risk of high NH_3_ volatilization (nearly 21–28%) more obviously in soil with high pH (Soares et al., 2012; Xia et al., 2017). Therefore, in summary, the trade-offs of comparative methods of fertilization of synthetic N fertilizers alongside mixed application with organic manure deserve further focused studies under different soil conditions for better evaluation of N dynamics and improving NUE. This will pave the way for the widespread adaptation of an aerobic rice system with higher profitability and no compromise on a healthy environment.

##### Accumulation and leaching of NO_3_^–^ to groundwater

The upland rice production is primarily characterized by high gross nitrification rates where after the incorporation of synthetic N fertilizers, rapid conversion of NH_4_^+^to NO_3_^–^ occurs under favorable saturation and optimized temperature conditions (Norton and Ouyang, 2019). Generally, the upper soil layer is majorly dominated by NO_3_^–^ except for a shorter time duration after the amendment of synthetic N fertilizers (Messiga et al., 2021). Higher soil NH_4_^+^ content and pH could greatly increase during the short-term application of synthetic N fertilizers, leading to high NH_3_ volatilization. The temporal and spatial division of NO_3_^–^ in the soil profile can be greatly modified by irrigation and fertilizer management; however, frequency and intensity of precipitation also play a key role (Huang T. et al., 2017). The higher NO_3_^–^ accumulation in the soil profile occurs due to several factors, including higher amendments in synthetic N fertilizers than crop demand, leading to excessive ecosystem damage (Chang et al., 2021a); intensive irrigation or heavy precipitations causing occasional leaching of NO_3_^–^ to subsoil surface leading to eutrophication (Huang J. et al., 2017); restricted denitrification of NO_3_^–^ because of higher oxygen content and limited availability of oxidizable C content (Huang et al., 2014); and poor immobilization of NO_3_^–^ by microbial communities (AOA, AOB, NOB, etc.) under differential availability of soil C (Qiu et al., 2013; Sawada et al., 2015).

Transportation or movement of NO_3_^–^ out of the reaches of the plant rooting zone is termed as leaching, which is affected by various factors, such as soil structure, soil texture, intensity and frequency of precipitation, and irrigation practices (Sebilo et al., 2013; Bijay-Singh and Craswell, 2021). In the upland rice systems, N leaching was denoted as inconsequent loss pathways because of higher evaporation than annual precipitation (Jiao et al., 2017; Jankowski et al., 2018; Sigler et al., 2020). Later, it has been found that frequent precipitation periods during summer could cause drainage of water leading to downward movement of NO_3_^–^, which is supported by soil textural factors (Huang T. et al., 2017; Manik et al., 2019; Hess et al., 2020; Kaur et al., 2020). Hence, it was summarized that the amount and the intensity of annual irrigation and precipitation impact the overall leaching of NO_3_^–^ (Huang J. et al., 2017). Constituting the relationships between leaching of NO_3_^–^ and application rates of N fertilizers either by synthetic or organic sources could provide sufficient information to estimate N leaching in similar soil-climatic conditions (Dybowski et al., 2020). Measurements covering several sites in different soil and climatic conditions to establish an empirical model denoting different drought, normal, and saturated years could serve beneficially in the estimation of NO_3_^–^ leaching for certain soil-climatic conditions because of the similarity of other variables which impact the leaching pathways (Woli and Hoogenboom, 2018; Rupp et al., 2021).

Synthetic N fertilizers have been widely used across the globe for higher crop production. The exceedingly high N content in intensively managed rice systems could lead to serious NO_3_^–^ contamination of groundwater via percolation and short-term recharge (Bastani and Harter, 2019). Once the concentration of NO_3_^–^ exceeds the standard values, it may take several years and even decades to recover (Wild et al., 2018). Following this, regulations in the use of synthetic N fertilizers undergoing climatic and soil conditions could play an important role in simultaneously decreasing the surplus N in the system, specifically NO_3_^–^ pollution of groundwater (Wick et al., 2012; Wild et al., 2018; Moloantoa et al., 2022). Although continuous efforts across the globe have reduced the NO_3_^–^ concentrations, most of the regions worldwide still have levels exceeding the standards and suffering from groundwater contamination (Abascal et al., 2022).

##### N_2_O emissions

In recent years, deep insights into the N cycle and advanced studies have been made in the understanding of the factors and processes involved in N_2_O emission pathways from different soils (Butterbach-Bahl et al., 2013; He et al., 2016; Wang et al., 2021a). In many circumstances, biological processes like nitrifier nitrification and denitrification, denitrifier denitrification, and coupled nitrification-denitrification share great roles in N_2_O emissions (Zhang et al., 2019a). It has been estimated that the application of synthetic N fertilizers or organic manures usually enhances the above-mentioned processes and N_2_O emissions. The share of different biological pathways in producing and emitting N_2_O majorly depends on soil conditions (pH, temperature, water content, and oxygen levels) and microbial C and N content (Signor and Cerri, 2013), where these factors are categorized as soil properties, climatic characteristics, and management measures. The temporal and spatial shifts in these factors are subject to changes in N_2_O production and emissions from cropping systems (Han et al., 2017; Dorich et al., 2020). To obtain reliable data for N_2_O emissions, conducting high-frequency experiments covering various sites with different soil and climatic conditions is necessary to develop mechanistic and statistical models and develop respective mitigation approaches (Ma et al., 2018; Zhang et al., 2022).

Under varying soil conditions, N_2_O production and emissions have been found to be closely related to soil NO_2_^–^ accumulation in soil profile after synthetic N fertilizer amendment (Sosulski et al., 2020). Synthetic N fertilizers increased soil N_2_O fluxes from 24.3 to 46.4%, and there has been a significant correlation between N_2_O fluxes and NO_2_^–^ concentration (Sosulski et al., 2020). When NO_2_^–^ exceeded 60 mg N kg^–1^ of applied N fertilizer, its reduction increased when the oxygen concentration levels were below 5% in accordance with nitrifier denitrification (Ju and Zhang, 2017). Moreover, it has also been observed that the accumulation of NO_2_^–^ and emissions of N_2_O are significantly dependent on the type of N fertilizer and amendment methods like mid-row band placement of N fertilizer increased the intensities of N_2_O and NO_2_^–^ relative to broadcasting (Maharjan and Venterea, 2013). In summary, several approaches have been proposed regarding mitigation of N_2_O emissions, which are as follows: optimization of the application rates of synthetic N fertilizers and manures and avoiding ammonia accumulation in the soil profile, slowing down of the ammonia oxidation pathways by different management approaches for nitrification, and replacing the conventional use of NH_4_^+^-containing N fertilizers with NO_3_^–^-containing N fertilizers.

### Factors important to increase N-use efficiency

Improvements in NUE under aerobic rice systems and other non-N_2_-fixing crops are required to fulfill the future food requirements of the projected inflation in the population via improved and modified N-use efficient varieties along with other crops, irrigation, and fertilizer management practices (Mboyerwa et al., 2021). Management practices that focus on improving plant N uptake and NUE involve irrigation methods, fertilizer input management, crop variety management in terms of water and NUE, plant N uptake management, use of controlled-release N fertilizers, use of NIs and urease inhibitors for synthetic N fertilizers, and other agronomic integrative crop management approaches (Pan et al., 2017; Gweyi-Onyango et al., 2021; Williams et al., 2021; Ebbisa, 2022; Ma X. et al., 2022). Several studies regarding NUE have demonstrated that it is majorly associated with the type and method of fertilizer inputs, irrigation methods, availability of soil N, and plant N uptake (Ye et al., 2013; Rawal et al., 2022). In modern agriculture, coupled management approach of irrigation and N management is an eco-efficient and widespread adopted technique, which can sustain rice yield under scarce water resources while ensuring environmental sustainability. It has been found that the coexistence of N and irrigational managements reduce N losses and application rates of N fertilizers, and improve NUE and irrigational water inputs nearly by 20% (Yang et al., 2020). Therefore, a comprehensive understanding and evaluation of all these above-mentioned factors impacting NUE and their management are effective in increasing the overall NUE under aerobic rice systems.

#### Irrigation and fertilizer inputs

Nitrogen recovery under the aerobic rice system is generally low, while it is prominently higher in the soil N pools by about two times than required (Zistl-Schlingmann et al., 2020). Sources for N fertilizers play an essential role in changing the overall NUE, such as controlled release in comparison with conventional synthetic fertilizers (Lawrencia et al., 2021), organic relative to inorganic N fertilizers (Tamele et al., 2020), and N fertilizers with the incorporation of new NIs. Besides, it has been proved that alternations in irrigation (Bagheri Novair et al., 2020) and modification in the methods and types of N amendments provides improved rice growth, yield, N uptake, and N utilization due to increased activities of glutamine synthase and glutamine synthetase in plant roots (Zhang et al., 2017). Currently, it is well-understood that continuous flooding is discouraging compared to AWD systems in increasing NUE where more improvements have been seen when applying slow-release N fertilizers along with NIs (Ye et al., 2013). However, a moderate AWD irrigation approach is an essential practice in integrative and progressive rice management to the enhanced NUE (Zhang et al., 2018a). But it has also been observed that only the AWD irrigation approach (without any amendments) significantly reduced the water inputs and improved N fertilizer control without having any negative impact on NUE in the aerobic rice system. Hence, it can be deduced that the alternative modified irrigation regimes, such as AWD or moderate AWD and furrow irrigation, are convenient for freshwater scarce areas to sustain future rice yield, improve NUE, and ensure a healthy environment (Jia et al., 2021).

#### Selection of rice cultivars

The selection of rice cultivars highly responsive to N fertilizers is necessary to decrease the N losses by increasing the plant N uptake, thereby improving NUE. These highly responsive rice varieties can readily absorb, utilize, and remobilize soil available N and encourage a sustainable rice production system (Mauceri et al., 2020; Giordano et al., 2021). Shifts in the concentration ratio of NH_4_^+^–N and NO_3_^–^–N content change the response of rice cultivars to soil available N, where cultivars with modified root features and highly responsive to soil N pools show increased N accumulation than the cultivars less responsive to soil N pools and with low NUE. Coupled organic and inorganic N fertilizer application can increase the rice grain yield but essentially requires the genotype selection process through integrated approaches of japonica and indica rice varieties (Farooq et al., 2021) via integrative progressive crop management that can improve NUE and other crop features in physiological and agronomic perspectives (Fan et al., 2021; Deng et al., 2022).

#### N forms as a particular index

Rice plants preferably uptake the NO_3_^–^ form of N whether under an aerobic or flooded system, which is considered as an indicator molecule for plant growth and physiological developments. It has been deduced that changes in NO_3_^–^ assimilation genes and the transporter can improve the NUE in aerobic rice systems (Iqbal et al., 2020a,b). Moreover, another study has demonstrated that bentonite hydro-char composites (BTHC) may reduce the abundance of archaeal *amoA* genes that probably limit the nitrification pathways and improve soil NH_4_^+^ N pools (Chu et al., 2020). NUE coupled with ammonia volatilization has not been evaluated comprehensively in aerobic rice systems; however, the application of N fertilizers in varying forms reduced the N losses among rice cultivars that are highly responsive to different soil N forms (Li Y. et al., 2017; Chen G. et al., 2020). High-affinity transporter systems among the rice cultivars are highly efficient in the utilization of soil available N and have an important function in the NUE of aerobic rice systems. Nitrite and nitrate-reductase activities are usually increased in cultivars poorly efficient in the utilization of soil available N under an aerobic rice system which ultimately demands an increase in N fertilizer inputs (Hakeem et al., 2011, 2016).

#### Role of microbial communities in rice soils

Paddy soils hold a confound diversity of soil microbial communities and soil microfauna, which mainly include N fixers, nitrifiers, denitrifiers, methanogens, methane oxidizers, plant growth regulators, phosphate-dissolving microfauna, sulfur oxidizers, decomposers, and nutrient recyclers (Prasanna et al., 2011; Manjunath et al., 2018; Zhu et al., 2020). The diversity and structural composition of microbial species vary under different textural and structural compositions of soil along with differences in climatic and other conditions. For example, relative to other soil habitats, rice soils generally have a prepotency of actinomycetes and Gram-positive bacteria. However, the microbial communities existing in floodwater have an abundance of Gram-negative bacteria and algae, whereas percolating water is commonly abundant only with Gram-negative bacteria (Kimura and Asakawa, 2005; Zhang et al., 2018d; Liu et al., 2021; Li et al., 2022). The microbial communities in paddy soil are mainly composed of large numbers of diversified fungal and bacterial species that function in numerous ecological activities (Omomowo and Babalola, 2019). In addition, several species of archaea, oomycetes, and other microbial species play crucial roles in mediating diverse ecosystem activities and other ecological functions that balance soil health and productivity (Wang et al., 2017, 2021b), and bacterial and fungal species have been observed to play leading roles under certain conditions. Generally, bacterial communities during differential soil conditions under the paddy system are mainly dominated by *Proteobacteria*, *Chloroflexi*, *Actinobacteria*, and *Acidobacteria*, whereas the dominant fungal communities include *Ascomycota*, *Basidiomycota*, and *Glomeromycota* (Yuan et al., 2019; Tian et al., 2021).

The diversification and activities of microbial communities existing in paddy fields are generally influenced by biotic and abiotic factors, which include temperature, precipitation, humidity, pH, agrochemicals, cations and anions, soil texture and structure, and the rice cultivar (Kim and Lee, 2020; Zheng and Lin, 2020). Agricultural management practices for aerobic rice, such as the method of crop establishment (direct-seeding) and the duration of alternate flooding and drying conditions during the rice growth period, also influence the microbial community structure and abundance. For example, it has been demonstrated that while under water-saturated soil conditions in the aerobic rice system, the symbiotic association between mycorrhizal fungi and lowland rice plant roots is restricted due to differential availability of oxygen content, as aerobic microbes similar to other fungi are greatly impacted by the amount of soil moisture content (Klinnawee et al., 2021).

Comparing the relative abundance of soil microbes, it has been observed that bacteria are the dominant group over archaea and fungi existing in the rhizosphere soil that surrounds plant roots, whereas among the bacterial phyla, *Proteobacteria* is the predominant phylum found in most paddy rhizosphere soils (Somenahally et al., 2021; Zhang et al., 2021). China-based research studies have demonstrated that the most abundant bacterial genera affiliated with the core microbial communities of rice rhizosphere soils under standard crop management are *Anaeromyxobacter*, *Arenimonas*, *Arthrobacter*, *Bacillus*, *Bellilinea*, and 15 other bacterial genera, all of which are well-known to share in the overall growth and health of rice plants (Zhou et al., 2020; Sahu et al., 2022). The community abundance and structural composition in the root rhizosphere soil of rice are impacted by cultivation practices, e.g., crop cultivated with organic soil amendments is harbored with several genera that potentially promote plant growth, which include *Anabaena*, *Azospirilllum*, and *Rhodobacter* (Edwards et al., 2018; Zhang et al., 2018b). Rice soils that are irrigated continuously occupy large numbers of methanogenic archaea that mediate the generation of GHG, i.e., CH_4_ emissions. In contrast, in rice soils that are alternatively kept under aerobic conditions, the dominance of methanotrophic bacteria is observed. They utilize methane gas for their own metabolic requirements, restricting it to emit into the atmosphere (Yvon-Durocher et al., 2014; Harman et al., 2021; Mahabubur Rahman and Yamamoto, 2021).

Plant roots release a wide range of compounds technically called as root exudates into their rhizosphere vicinities in the form of sugars, aromatic acids, amino acids, polysaccharides, aliphatic acids, and fatty acids that pull microbes to form a mutualistic affiliation. The pattern and composition of such root exudates emphatically influence the makeup and abundance of microbial communities in the rice rhizosphere (Checcucci and Marchetti, 2020; Jamil et al., 2022). The domestication of rice, in terms of its species and origins, has also been impacted by the composition of fungal communities in the rhizosphere, e.g., five genera of arbuscular mycorrhizal fungi (AMF) (namely, *Claroideoglomus*, *Acaulospora*, *Redeckera*, *Pacispora*, and *Scutellospora*) are significantly positively associated with other fungal species in the rhizosphere of wild rice, while marginally a set of only three groups of AMF (*Claroideoglomus*, *Gigaspora*, and *Redeckera*) are strongly positively correlated with other fungi that harbor in the rhizosphere of domesticated rice varieties (Schmidt et al., 2020; Chang et al., 2021b).

**FIGURE 5:**
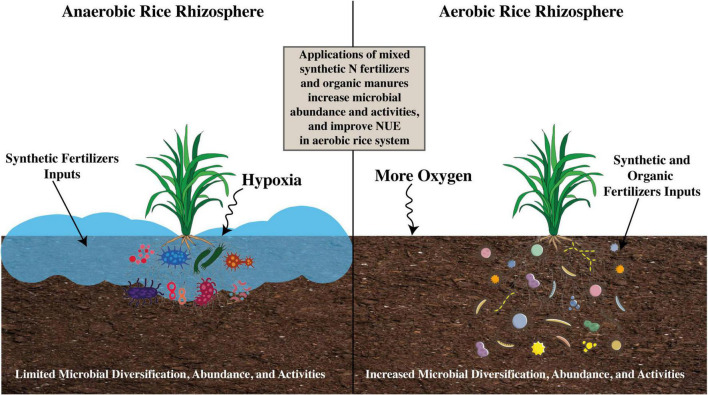
Impact of soil management practices on microbial community abundance and activities under varying soil conditions (Rachwał et al., 2021; Wipf et al., 2021; Beule et al., 2022).

Soil cultivation practices also impact arbuscular mycorrhizal fungi. When the AMF abundance harboring rice rhizosphere grown under aerobic rice system was compared with those found under traditional rice cultivation methods, all the samples of AMF in the roots taken from conventional rice system belonged to just one genus, *Glomus*, whereas samples being taken from aerobic soil environment belonged to *Acaulospora* and *Glomus* (Bernaola et al., 2018; Davidson et al., 2019). Several research studies have demonstrated legion plant-beneficial microbes, which include *Aspergillus, Bacillus*, *Trichoderma*, *Clostridium*, *Penicillium*, and *Azotobacter* that are more abundant in the rice rhizosphere soil under the aerobic system (Carvalho and Castillo, 2018; Chamizo et al., 2018; Khadka and Uphoff, 2019). Hence, the above discussion further provides evidence that soil and crop management practices have strong effects on the microfauna of the rice rhizosphere. Figure 5 presents insights into how crop and soil management practices in aerobic rice systems influence the activities, abundance, and structural composition of soil microbial communities.

#### Effects of management measures on differential roles of ammonia oxidizers (ammonia-oxidizing archaea and ammonia-oxidizing bacteria)

Several studies have observed the effects of various fertilizer managements on AOB, which reported that although organic and inorganic fertilizer amendments together increased the relative abundance of 16S rRNA gene copies, plant growth and developmental phase played a key role in the structural composition and relative abundance (Tang et al., 2019; Hernández-Guzmán et al., 2022). In contrast, another study has reported that change in fertilizer regimes rather than plant growth stage played a central role in changing the relative abundance and structural composition of AOB communities (Chen et al., 2014; He et al., 2021). Hence, it can be concluded that changes in N fertilization regimes make the AOB more sensitive and can be used as an index for soil N availability (Wang F. et al., 2018; Xu et al., 2022). The incorporation of different organic and inorganic N fertilizers changes the soil physical and chemical properties by altering the forms and amount of essential nutrients, which consequently change the abundance and the structural composition of AOB communities (Farooq et al., 2022a). Synthetic N fertilizers, such as urea, increase the soil NH_4_^+^ content, an important substrate providing the necessary energy for ammonia oxidizers and increasing their abundance as well (Mahmud et al., 2021). It is hypothesized that organic N available in the soil should be mineralized into such available form necessarily required by AOB; however, the slow release of N may increase the competition among plants, heterotrophs, and AOB (Taylor et al., 2021). Other soil properties, such as pH, negatively influence the abundance of ammonia oxidizers, more specifically AOB abundance, depicting that moderate acidic soil conditions favor the abundance and activities of AOB over moderate alkaline soil conditions (Wessén, 2011; Yao et al., 2011; De Gannes et al., 2014). Phylogenetic cluster analysis of *amoA* gene has determined that most AOB were belonging to *Nitrosospira* under varying applications of organic and inorganic N fertilizers (Saiful Alam et al., 2013; Zhao et al., 2015). Hence, it is concluded that different management measures for N fertilization change the soil available N regimes and ultimately also change the relative abundance and structural composition of AOB (Min et al., 2016).

Considering the relative abundance of AOA under differential soil conditions, *Nitrososphaera* and *Nitrosopumilus* clusters have been indicated as the most abundant AOA communities (Prosser and Nicol, 2012; Li et al., 2018; Yao et al., 2022). Structural composition and relative abundance of AOA communities are inclined to be less influenced by variations in soil N regimes and environmental conditions as compared to AOB (Liu et al., 2014; Lu et al., 2018; Zou et al., 2022). Eco-physiology analysis of AOA communities has depicted the dual ways to gain their energy both by oxidizing NH_4_^+^ to NO_2_^–^ autotrophically and by assimilation of carbon and other energy sources from organic substrates heterotrophically (Kerou et al., 2016; Ma M. et al., 2022). Disordered response of AOA to different N fertilization regimes has been observed, which might be anticipated due to the effects of plant growth stages, environmental variations, and other soil factors which might have altered the activities of AOA autotrophically and heterotrophically (Amoo and Babalola, 2017). Previous studies have reported contrasting results about the relative abundance and structural composition of AOA communities. Some studies demonstrated that different N fertilizer management approaches significantly impact the AOB communities over AOA (Sun et al., 2019; Ding et al., 2020), whereas a few studies revealed that the structural composition and the relative abundance of AOA communities were significantly sensitive to different fertilizer management strategies (Mukhtar et al., 2019). Amazingly, the structural composition of AOA has been noticed to depict an intensive correlation between AOA composition and soil properties and environmental conditions, which necessarily reported that a single soil or environmental parameter does not decide the structural composition of AOA in the soil (Jiang et al., 2014; Zhang et al., 2019a).

Various factors are involved in influencing the abundance and structural composition of ammonia oxidizers and require different optimum growth temperatures (Zeng et al., 2014; Lehtovirta-Morley, 2018). Larger soil temperature variations ranging from 9 to 31°C partially contribute to the differences in the structural composition and abundance of AOA and AOB (Liu J. et al., 2018; Wang and Huang, 2021). The growth stage of rice also greatly influences the abundance of ammonia oxidizers, as the AOB and AOA were found at peak abundance during the heading stage (Song and Lin, 2014; Wang et al., 2017). Water management, dissolved oxygen, and soil salinity status are other factors along with plant growth that influence the abundance and composition of AOA and AOB (Chen et al., 2017; Guo et al., 2020).

## Toward better N management

### Management of N cycle

**FIGURE 6:**
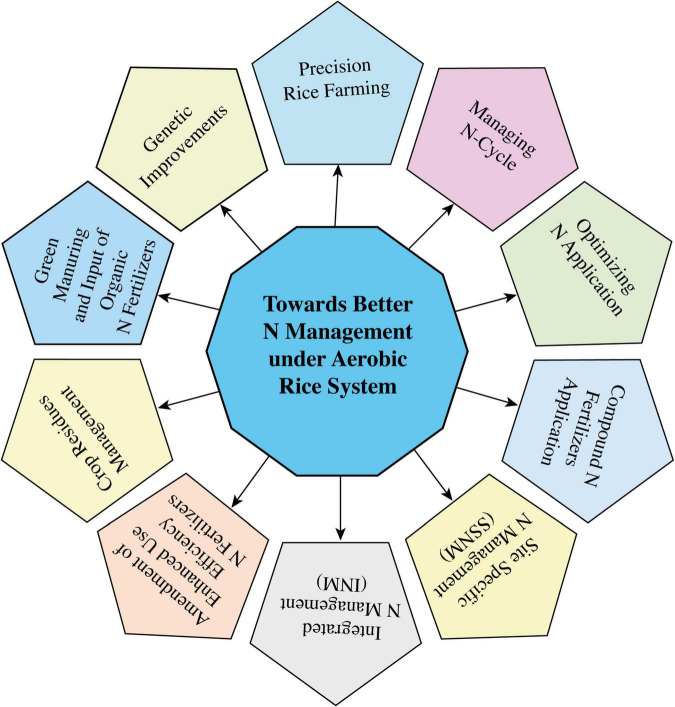
Eco-efficient management strategies to improve NUE under the aerobic rice system (Farooq et al., 2022a).

The NUE of different agricultural systems, especially rice (Figure 6) and livestock in Asia, is very low when compared to China, and this efficiency ranged between 40 and 15%, respectively, whereas the N recycling ratio of livestock manures to agricultural systems was nearly 43% (Ju and Zhang, 2017). On the contrasting side, among other developed countries of Europe and America, the NUE of agricultural systems and livestock and N recycling ratio of livestock manure were about 60, 20, and 80%, respectively (Wu et al., 2018; Zhan et al., 2021). Therefore, there is a large management gap in the attributes of the N cycle across the globe, which requires integrated approaches to make improvements in NUE, particularly in underdeveloped and developing countries (Erisman et al., 2018). In agricultural systems, due to heavy N losses during crop production and animal rearing, and the fake conceptual facts that animal- and human-based wastes cannot be efficiently recycled to crop production systems, overall NUE remains low. Thus, maintaining or improving the rice productivity under modified rice systems can only depend on the continuously increasing use of synthetic N fertilizers, which ultimately lead to further environmental hazards. There can be several ways to improve the N cycling process and provide a solution to this state of uncertainties, such as by reducing the excessive inputs than demand and synchronizing the fertilizer inputs with crop growth stages (Liu J. et al., 2016), reducing N inputs and N losses to the environment for improved NUE in the aerobic rice system (Mboyerwa et al., 2022), and by relinking the crop production systems with livestock systems to properly incorporate their manures to increase N recycling rate and crop yield to fulfill food security aims (Sekaran et al., 2021).

### Optimized inputs of N fertilizers

The current study not only focuses on the management of N fertilizer application, but also emphasizes N management of the applied fertilizers and how could applied fertilizer input be better utilized by plants (Figure 6). Under aerobic rice cultivation, which is an intensive agricultural product system, potential target grain yields can only be attained through external N inputs except for indigenous N supply in the soil (Bailey-Serres et al., 2019). Mineral N fertilizers should essentially be applied with full use of organic manures and crop straw incorporation or biologically fixed N to fill up the soil N consumption (Ladha et al., 2022; Shi et al., 2022). Moreover, should consider the use of mineral N fertilizers only when the organic manures and other non-synthetic N fertilizers cannot meet plant N demands (Geng et al., 2019). Currently, upland rice systems are the most intensive agricultural systems where synthetic N fertilizer is the prevailing form of N inputs across the globe (Liu T. et al., 2018; Castillo et al., 2021; Yuan et al., 2021). Generally, proper management of synthetic N fertilizers includes attributes of inputting the fertilizer in the form of the right nutrient sources, in the optimized quantity, and synchronizing the time and place of fertilizer input with plant growth and development (Johnston and Bruulsema, 2014). These features are associated with each other, as the N application rate not only depends on potential yield but also on input methodologies, timing, and types. If the synchronization of the former with the latter three aspects was not undertaken as correct, most of the applied N input would be lost to the ambient environment, and the amended fertilizer would not meet the plant requirements. Therefore, farmers would require more inputs of N fertilizers to achieve the target yield. Currently, the N input rates for major cereals, such as rice, wheat, and maize, are very high ranging between 150 and 250 kg N ha^–1^ for target grain yields because most of the N fertilization is done through the broadcast method which leads to ammonia volatilization (Guo et al., 2022). So, the input of mineral and non-synthetic N fertilizers should be done properly not only for good crop yield, but also for environmental sustainability. The aim of optimizing the N inputs should be fulfilled in such a way so as to reduce the increased use of synthetic N fertilizers without compromising the yield. To achieve this aim, N input methodologies must be improved to reduce the N losses, hence permitting a decrease in the overall N fertilizer input rate without compromising the potential quantity and quality of the crop yield along with environmental sustainability.

Taking an example of low NUE in intensive agricultural systems of Northern China, if the soil is amended with 300 kg N ha^–1^ during the respective growing season, the total available N fertilizer for plant uptake would be 171 kg N ha^–1^, where the N losses would be nearly 43% under conventional cropping systems. Meanwhile, If N losses are reduced by undertaking optimized fertilization application techniques and balancing the available N fertilizer for plant uptake, the actual required amount of N fertilizer could be reduced by 78 kg N ha^–1^ to a total required nearly 222 kg N ha^–1^ (Ju and Zhang, 2017). Realization of the importance of quantity and type of N fertilizers with better synchronization between fertilizer application place and methodologies is still challenging across the globe. Partially, this challenge could be justified by the presence of a technical issue of how to merely determine the quantity of N fertilizer based on the estimated potential yield. Moreover, determining the overall N supply from organic manures is also a technical problem at the farmer’s scale. Regionally, various cost-effective measures involving the concepts and methodologies of N supply rate from different fertilizers should be adopted to decide on the optimized N fertilizer application which could ultimately be helpful in maintaining soil health and avoiding environmental hazards (Singh, 2018). Another challenge that hampers the proper management of N fertilizers in intensive agricultural production systems is the small landholdings when considering the economic as well as social aspects. Therefore, proper management of cropping system transfer and policy makings for small landholders with managed awareness systems are essentially required to be undertaken and advocated by government agencies potentially aiming toward the solution of the projected future challenges of low NUE in aerobic rice system to save the economic resources without sacrificing agricultural production and environmental health (Anas et al., 2020).

### Application of N compounds

Currently, several types of N compounds exist that increase the plant N uptake and overall NUE in different aspects without causing any environmental damage (Figure 6). Considering the N accumulation in rice grains and straws, it has been noticed that conventional synthetic urea functioned badly over controlled-release urea (Wang L. et al., 2018). BTHC are the compounds that have been noticed to decrease ammonia volatilization when applied during rice flooding conditions, and it subsequently leads to an increased grain yield and NUE (around 40%) (Chu et al., 2020). Moreover, it has been demonstrated that the application of zeolite compounds along with nitrifying bacteria increased crop biomass and NUE while decreasing the ammonia volatilization N losses (Cataldo et al., 2021). The application of organic manures, such as rice straw and synthetically manufactured composts like Azolla compost, significantly increased the concentration of total soil N pools and also improved the soil structure and nutrient recycling (Bagheri Novair et al., 2020; Seleiman et al., 2022).

### Agricultural management

Agricultural management aiming to increase NUE without compromising the crop productivity should be in accordance with local soil and climatic conditions which could involve intensive and extensive soil and crop residue management practices, as the N harvest in grains and soil N pools vary between intensive and extensive approaches (Sarkar et al., 2020; Zistl-Schlingmann et al., 2020). Another challenge that is associated with the aerobic rice systems is regarding tillage practices, as consecutive no-tillage or normal tillage practices accompanied an increase in N- and water-use efficiencies (Habbib et al., 2019); however, they increased the crop competition due to higher weed invasion. Hence, integrated crop management measures along with advanced irrigation management practices are required in aerobic rice systems for higher NUE and crop productivity. Some of the agricultural management strategies to improve NUE are discussed in the following section.

#### Site-specific nutrient management

During recent decades, an approach termed site-specific nutrient management (SSNM) was designed aiming to increase the nutrient-use efficiency of major nutrients, such as N, phosphorus (P), and potassium (K), and to generate field-specific fertilizer recommendations (Chivenge et al., 2022). The SSNM is a dynamic, generally plant-based, soil and crop growth season-specific nutrient management approach which intends to synchronize nutrient provision and demand based on the differences in plant requirements, indigenous nutrient provision, and nutrient recovery from applied fertilizers and other resources (Figure 6). The major focus of SSNM is to improve the crop yields and economic outputs for the local farmers by increasing nutrient-use efficiencies (Rodriguez, 2020). Field-based and growth season-specific fertilizer requirements for a specific crop are measured before the start of the respective crop season to properly plan the protocol considering the climatic and soil conditions to better synchronize with critical growth stages (Hulmani et al., 2022). Therefore, plant fertilizer requirements should necessarily be calculated aiming to reduce the production losses due to nutrient deficiencies and avoid mining the soil nutrients while ensuring the profitability that could only be possible with proper synchronization with potential crop demands under differential conditions (Sharma et al., 2019). For N fertilizer inputs, SSNM determines the optimum amount of fertilizer that is required to be applied, and further calculates the balanced supply throughout the crop growing season to synchronize the peak demands for N by the plant, depending on the needs of crop varietal types, soil texture and structure, and variations in the environmental conditions (Peng et al., 2010). Further approaches, including leaf color charts, can also be utilized for additional in-season modifications of the projected N amendments, therefore empowering further tweaking against the actual crop growth conditions (Chivenge et al., 2022). SSNM was initially conceived in Asia for smallholder rice producers, where field occupations tend to be small with large spatial variabilities regarding the soil nutrient statuses and management. This nutrient management technique is generally based on the principles of estimating the nutrient requirements by evaluating the gap between aggregated quantity of nutrients required to attain specifically targeted crop production and the indigenous supply of that specific nutrient (Sharma et al., 2019). Hence, this approach could encourage the optimized application of N fertilizers in aerobic rice systems through adjustments in the timing of N fertilizer amendment to fulfill the potential N requirements of the plant to improve the NUE and avoid environmental hazards (Rodriguez, 2020).

#### Integrated nutrient management strategies

Integrated nutrient management (INM) includes the optimum and balanced use of indigenous N components, i.e., plant residues, organic manures, biological N fixation, mineral N fertilizers, and their complementary interactive pathways to increase N recovery and NUE (Sarwar et al., 2021). The positive impacts of the INM approach involving the mixed use of organic (e.g., biochar, manures, and compost) and inorganic N resources are either due to optimization of physico-chemical properties of soil (Oladele et al., 2019) or due to better plant root growth and improved supply of required N along with other micronutrients (Thind et al., 2012). The proper evaluation and understandings of the interactive pathways under INM are necessary to properly exploit the positive impacts which are critical in increasing the net economic returns to the farmers in terms of targeted yield while maintaining the soil quality and improving NUE (Figure 6). The complementary interactions of N supplied from synthetic and non-synthetic fertilizers with other micronutrients could lead to a considerable increase in crop production and NUE. Therefore, balanced and judicious use of N fertilizers from all available resources (organic and inorganic) through INM approaches will subsequently lead to higher aerobic rice production for future rice sustainability under a scarcity of resources in face of climate change, improved NUE, and minimum environmental hazards.

#### Incorporation of enhanced use efficiency N fertilizers

Currently, the beneficial use of several fertilizer products is they have the capabilities to improve the NUE of applied N fertilizers by limiting N losses linked with the rice production system (Figure 6). These fertilizer products are principally based on certain features, such as either they can slow the release rate of N or can intervene with N transformation pathways and reduce N losses. Slow- and controlled-release N fertilizers are the forms of N fertilizers having significant roles in controlling various N losses, thus impacting N availability and recovery (Ransom et al., 2020). Relative to NH_4_^+^-containing N fertilizers, NO_3_^–^-containing N fertilizers are more susceptible to leaching, whereas the former is more prone to volatilization loss than the latter. A range of slow- and controlled-release N fertilizers are now commercially available, and their utilization along with potential mitigation strategies leads to a sustainable agricultural production system by limiting N losses and thereby improving NUE (Mahmud et al., 2021). These slow- and controlled-release N compounds can reduce N losses due to their potential to delay the N release patterns which can improve the synchronization between plant N requirements and soil N supply. In addition, neem-coated commercial urea can also be widely used in aerobic rice systems, as it is demonstrated to be a slow-release N fertilizer in South Asia (Naz and Sulaiman, 2017). However, the widespread usage of such controlled-release N fertilizers is still challenging due to higher manufacturing costs and limited availability, which necessitates the need for measures that can extend the use of these compounds.

### Resource conservation and management practices

#### Residue management

The plant parts left in the barren field after crop harvesting are termed as crop residues, which can play a vital role in plant growth and development, as they affect the availability of nutrients to plants due to the addition of organic manure to the soil (Figure 6). Crop residues play the principal role of source and sink for C and N cycles. Incorporation of crop residues into the soil ensures the supply of N to the plants for a longer period initially by conversion of N to inorganic forms and thereby mineralizing it at later crop growth stages when crop demand for N becomes substantial. Moreover, the incorporation of crop residues into the aerobic rice system can be beneficial because soil microbes temporarily immobilize the nutrients that are released into the soil and conserve the nutrients in slowly available forms. Hence, plants cannot uptake all the nutrients at once, but nutrients may become available throughout the growing season or to the succeeding crop (Sarkar et al., 2020). It has already been demonstrated that plant residues of several cereals can ensure the provision of N ranging between 40 and 100 kg ha^–1^ season^–1^ and assist in the accumulation of organic C within the soil system which further improves the soil N pool and NUE (Hu et al., 2015; Roozbeh and Rajaie, 2021). Incorporation of legume residues into the aerobic rice system can be an effective source of increasing soil N pools because of having high N content and lower C/N ratios relative to cereals (Moore et al., 2019). Moreover, plant residues of legumes have quicker rates of mineralization, and half of the total amount of N would be available for plant uptake within 2 months of residue incorporation (Wang et al., 2019).

#### Green manuring

There is a wide range of legume crops that have a superior capacity to fix atmospheric N, and therefore can be used for green manuring under aerobic rice systems (Qaswar et al., 2019). Annual N accumulation by legume crops ranges between 20 kg ha^–1^ and 300 kg ha^–1^ (Thind et al., 2012; Ali et al., 2015). To make the legume crops potentially superior in green manuring, they should be accompanied with important features of quick and shorter growth duration for easy settlement into an intensive cropping system, capabilities to produce large dry matter, should potentially fix atmospheric free N, and should be cultivated with minimum land management practices (Sharma et al., 2011). However, the beneficial impacts and efficiency of legumes, serving as green manures, largely depend on the quantity and quality of residues available, soil properties, microbial diversity and activities, the moisture content in the soil, and atmospheric variables. Hence, proper crop rotation of legumes with aerobic rice systems and cultivation of crops in a recurring succession on the same piece of land is critical to enhancing the N recovery and overall NUE (Figure 6). Moreover, the use of optimum crop sequences ascertains the efficient utilization of agricultural resources, more obviously the nutrients and soil moisture content for the long-term sustainability of the production system. The inclusion of legume crops in a rotation system with cereals is an old-age approach and has been demonstrated as an efficient crop management practice to improve soil health and crop production (Stanger et al., 2008). It has been found that legume crops have the potential to reduce the need to succeed crops in the rotation system, and narrow C:N ratios of legume residues can ameliorate the physical, chemical, and biological properties of the soil by increasing total C stocks, which further lead to increased N availability to the plants (Vogel and Below, 2018).

### Genetic improvement and precision farming

Genetic improvement through breeding approaches by the introduction and selection of several quality traits responsible for efficient N utilization can also enhance NUE (Figure 6). Different rice genotypes may produce different quantities of grain yield with differences in NUE under the same amount of N fertilizer application. On a long-term basis, differences in the NUE and N recovery may originate from differences in the efficiency of absorption and assimilation of NH_4_^+^ ions, microbial activities, and their diversity (Sa̧dej and Przekwas, 2008); the features of roots, such as the extent, distribution, age, and root-induced shifts in the rhizosphere impacting mineralization, transformation, and transport (Rahman et al., 2014); and root-associated biological N fixation. However, variations in the efficiency of internal N use by crop plants may arise from differences in the internal plant N requirements at different growth stages (Sharma et al., 2021); capacity to translocate, disperse, and mobilize the absorbed N to and from various plant organs (Masclaux-Daubresse et al., 2010); import-export of N in flag leaf (Nehe et al., 2018); leaf senescence patterns; and CO_2_ fertilization and its conversion into carbohydrates (Cao et al., 2020). Therefore, proper understanding, identification, evaluation, and incorporation of such traits into the aerobic rice production system via improved breeding approaches may help in improving the NUE of this transformed rice system.

Precision farming is an information- and technology-based farm input management system which targets the use of innovative technologies and principles in identifying, analyzing, and managing spatial and temporal variabilities associated with all characteristics of crop production systems to maximize the net profits, crop production, resource-use efficiency, and ensure the system sustainability in terms of quality and quantity (van Evert et al., 2017). Measurement of variabilities in the field conditions with respect to the availability of N for plant uptake and application of optimum of dose of N fertilizers at the right time by the utilization of the technologies, such as rate applicator, remote sensing, geographic information system (GIS), and global positioning system (GPS), may play a key role to increase the NUE under varying climatic conditions (Yousefi and Razdari, 2015). Local or remote N sensors can also be employed in advanced crop management systems to appraise crop requirements for supplemental N. Therefore, the deployment of the above-mentioned technologies and precision agriculture practices can be helpful to increase the NUE in intensive agricultural systems.

## Conclusion

### Transformation in flooded rice system for future sustainability

The conventional rice system has been challenged to sustain the higher rice production under future projected climate change and scarcity of resources, which mainly involve the inputs like freshwater, labor, nutrients, and energy. The aerobic rice system is an innovative transformation of the flooded rice system under specific soil conditions with no requirements of continuous soil saturation and puddling. It can be considered as an eco-efficient rice production system with high water-use efficiency due to less water requirements while land preparation, no specific transplanting needs, and less labor cost during the whole growth duration period. However, reduced grain yield in the aerobic rice system due to heavy N losses necessarily requires the research to focus on several agronomic management measures for future widespread adaptation and food security. If proper management measures to cope with major challenges of higher N losses and weed invasion are ensured, the aerobic rice system can be a sustainable approach to balance the future food demand under resource-deficit conditions. Provision of the optimized conditions coupled with agronomic adjustments for N losses in aerobic rice systems can reduce the yield gap between traditional and transformed rice systems. Ensuring the provision of integrated N management measures to optimize the soil chemical and physical characteristics under the aerobic rice system can ensure timely seed sowing which leads to higher stand establishment ultimately producing higher grain yield.

Shifting from an anaerobic to aerobic rice system brings in the challenge of high N losses due to shifts in soil N pools, water availability, and microbial activities, thus leading to low NUE and reduced grain yield. For the widespread adaptation of the aerobic rice system, there is a need to introduce such rice cultivars that can perform well under aerobic soil conditions occupying better root characteristics for better N uptake and N utilization, better yield traits of lowland rice cultivars, and enhanced water stress tolerance traits of upland cultivars. Moreover, improved breeding techniques play an important role and mainly focus on physiological traits, including changes in chlorophyll pigments, protein, fat, and carbohydrates content, anthesis, relative water content, osmotic adjustments, and enzyme functioning. Undertaking the research-based evaluation of agronomic management measures for aerobic rice system is necessary to reduce the N losses and increase NUE, which may subject to sustain the grain yield and ensure environmental sustainability under the future projected climate change and scarcity of inputs.

### Rationale and major features of the current study

Higher N loss is one of the major challenges associated with the sustainability of the aerobic rice system. Currently, the aerobic rice system can sustain rice production like the traditional rice system but at the cost of high N losses, threatening environmental safety which hinders the widespread adaptation of this transformation. Therefore, the adaptation of the aerobic rice system at a wider scale under climate change conditions essentially requires deeper insights into the advances in pathways of the N cycle under aerobic soil conditions, thereby suggesting management approaches to adjust these pathways aiming to reduce N losses. Therefore, this review article was designed to discuss the challenges to rice production due to global climate change, investigate the need for transformational approaches in conventional rice system, and narrow down to project the importance of NUE in aerobic rice system under a future projected scarcity of resources and environmental hazards. This study discusses the recent advances in the N cycle under varying soil conditions by comparing both conventional and aerobic rice systems, and enriches the advanced knowledge about N losses and NUE under varying soil conditions. This study focuses on the dire need for complete reinvestigation and reevaluation of the changes in the pathways of the N cycle (ammonification, nitrification, denitrification, volatilization, and leaching) due to the replacement of conventional methods with an aerobic rice system, as several novel developments have depicted shifts in N cycle during this transformation. This study also discusses that N losses either in the form of gaseous volatilization or leaching are high under the aerobic rice system due to coupled nitrification-denitrification processes under the AWD irrigation approach, making it less economic and restricting its successful adaptation. Moreover, this study unveils the roles of important microbial communities, how an aerobic rice system can be an eco-efficient production approach through management measures, and which factors impact the management measures and hinder high NUE under this transformed rice system. This study discussed the interactive management mechanisms of how proper management of the N cycle can be accomplished through optimized N fertilizer inputs along with compound form amendments. Meanwhile, several agricultural adjustments, such as SSNM, INM, and incorporation of enhanced use efficiency N fertilizers, are also suggested that may interactively impact the activities and abundance of microbial communities involved in the overall N cycle and improve plant N uptake. Moreover, resource conservation and management approaches like crop residue management, green manuring, improved genetic breeding, and precision farming measures are intimated to increase the NUE under the aerobic rice system. Hence, it has been concluded that after careful insights into the pathways of the N cycle under aerobic soil conditions, incorporation of the suggested agronomic adjustive measures can reduce N losses, thereby enhancing NUE under the aerobic rice system while ensuring higher grain yield and environmental safety.

### Future thrust and few open-ended questions

Globally, for widespread adaptation of the aerobic rice system, it is necessary to conduct research focusing on how interactive activities of microbial communities, such as ammonia oxidizers, nitrifiers, and denitrifiers, change under varying soil conditions. Currently, a major challenge associated with the aerobic rice system is the limited understanding of the relationships among different microbial communities when incorporating any N fertilizer management measure. Due to limited knowledge about interactive features among N management measures, and shifts in microbial communities and their functioning, NUE in aerobic rice system is really infelicitous. Moreover, the interactive evaluation of spatial and temporal changes in abundance, diversity, and functioning of microbial communities under aerobic soil conditions while undertaking any of the agronomic management measures is still limited. Incorporation of agronomic management measures for aerobic rice system aiming toward increasing NUE while reducing the N losses may likely impact the activities and abundance of soil microbial communities and soil biochemical features. Therefore, coupled understanding of changes in soil biochemical properties and microbial communities while incorporating N management approaches can additionally favor in increasing the overall NUE in the aerobic rice system. Thus, further research studies on aerobic rice systems are encouraged to design experiments focusing on the interactive evaluation of shifts among activities and diversity of microbial communities, plant N demands, and N management measures. This will improve the knowledge and understanding of the response mechanisms in terms of abundance, diversity, and activities of soil microbial communities under different agronomic N management practices that may further help in improving NUE and reducing N losses for higher grain yield and environmental safety.

## Author contributions

MF and HF gestated the idea and collected and compiled the relevant literature for the study. MF and MU visualized and validated the figures. MF, HF, OR, ZM, MY, MK, SF, XW, and MU helped in the write-up of the original draft. All authors carefully read, revised, and approved the article for submission.

## References

[B1] Abascal E. Gómez-Coma L. Ortiz I. Ortiz A. (2022). Global diagnosis of nitrate pollution in groundwater and review of removal technologies. *Sci. Total Environ.* 810:152233. 10.1016/J.SCITOTENV.2021.152233 34896495

[B2] Abbasi H. Vasileva V. Lu X. (2017). The Influence of the Ratio of Nitrate to Ammonium Nitrogen on Nitrogen Removal in the Economical Growth of Vegetation in Hybrid Constructed Wetlands. *Environments* 4:24. 10.3390/environments4010024

[B3] Ahmadikhah A. Marufinia A. (2016). Effect of reduced plant height on drought tolerance in rice. *3 Biotech* 6:221. 10.1007/S13205-016-0542-3/TABLES/7 PMC5061651 28330293

[B4] Ainsworth E. A. (2008). Rice production in a changing climate: a meta-analysis of responses to elevated carbon dioxide and elevated ozone concentration. *Glob. Chang. Biol.* 14 1642–1650. 10.1111/J.1365-2486.2008.01594.X

[B5] Albina P. Durban N. Bertron A. Albrecht A. Robinet J.-C. Erable B. (2019). Influence of Hydrogen Electron Donor, Alkaline pH, and High Nitrate Concentrations on Microbial Denitrification: A Review. *Int. J. Mol. Sci.* 20:5163. 10.3390/ijms20205163 31635215 PMC6834205

[B6] Ali A. M. Thind H. S. Sharma S. Yadvinder-Singh. (2015). Site-Specific Nitrogen Management in Dry Direct-Seeded Rice Using Chlorophyll Meter and Leaf Colour Chart. *Pedosphere* 25 72–81. 10.1016/S1002-0160(14)60077-1

[B7] Ali S. Liu Y. Ishaq M. Shah T. Abdullah, Ilyas A. (2017). Climate Change and Its Impact on the Yield of Major Food Crops: Evidence from Pakistan. *Foods* 6:39. 10.3390/FOODS6060039 28538704 PMC5483611

[B8] Amanullah Hidayatullah. (2016). Influence of Organic and Inorganic Nitrogen on Grain Yield and Yield Components of Hybrid Rice in Northwestern Pakistan. *Rice Sci.* 23 326–333. 10.1016/J.RSCI.2016.02.007

[B9] Amanullah, Khan S. U. T. Iqbal A. Fahad S. (2016). Growth and Productivity Response of Hybrid Rice to Application of Animal Manures. Plant Residues and Phosphorus. *Front. Plant Sci.* 7:1440. 10.3389/FPLS.2016.01440 27803701 PMC5067482

[B10] Amoo A. E. Babalola O. O. (2017). Ammonia-oxidizing microorganisms: key players in the promotion of plant growth. *J. Soil Sci. Plant Nutr.* 17 935–947. 10.4067/S0718-95162017000400008 27315006

[B11] Anas M. Liao F. Verma K. K. Sarwar M. A. Mahmood A. Chen Z. L. (2020). Fate of nitrogen in agriculture and environment: agronomic, eco-physiological and molecular approaches to improve nitrogen use efficiency. *Biol. Res.* 53:47. 10.1186/s40659-020-00312-4 33066819 PMC7565752

[B12] Aryal J. P. Sapkota T. B. Krupnik T. J. Rahut D. B. Jat M. L. Stirling C. M. (2021). Factors affecting farmers’ use of organic and inorganic fertilizers in South Asia. *Environ. Sci. Pollut. Res.* 28 51480–51496. 10.1007/S11356-021-13975-7/TABLES/10 PMC8458167 33982263

[B13] Awan M. I. Bastiaans L. van Oort P. Ahmad R. Ashraf M. Y. Meinke H. (2014). Nitrogen use and crop performance of rice under aerobic conditions in a semiarid subtropical environment. *Agron. J.* 106 199–211. 10.2134/agronj2013.0262

[B14] Bagheri Novair S. Mirseyed Hosseini H. Etesami H. Razavipour T. (2020). Rice straw and composted azolla alter carbon and nitrogen mineralization and microbial activity of a paddy soil under drying–rewetting cycles. *Appl. Soil Ecol.* 154:103638. 10.1016/J.APSOIL.2020.103638

[B15] Bailey-Serres J. Parker J. E. Ainsworth E. A. Oldroyd G. E. D. Schroeder J. I. (2019). Genetic strategies for improving crop yields. *Nature* 575 109–118. 10.1038/S41586-019-1679-0 31695205 PMC7024682

[B16] Balestrini R. Brunetti C. Chitarra W. Nerva L. (2020). Photosynthetic Traits and Nitrogen Uptake in Crops: Which Is the Role of Arbuscular Mycorrhizal Fungi? *Plants* 9:1105. 10.3390/PLANTS9091105 32867243 PMC7570035

[B17] Balmaseda M. A. Hernandez F. Storto A. Palmer M. D. Alves O. Shi L. (2015). The Ocean Reanalyses Intercomparison Project (ORA-IP). *J. Operat. Oceanogr.* 8:s80–s97. 10.1080/1755876X.2015.1022329

[B18] Balmaseda M. A. Trenberth K. E. Källén E. (2013). Distinctive climate signals in reanalysis of global ocean heat content. *Geophys. Res. Lett.* 40 1754–1759. 10.1002/GRL.50382

[B19] Banning N. C. Maccarone L. D. Fisk L. M. Murphy D. V. (2015). Ammonia-oxidising bacteria not archaea dominate nitrification activity in semi-arid agricultural soil. *Sci. Rep.* 51:11146. 10.1038/srep11146 26053257 PMC4459192

[B20] Barth G. Otto R. Almeida R. F. Cardoso E. J. B. N. Cantarella H. Vitti G. C. (2019). Conversion of ammonium to nitrate and abundance of ammonium-oxidizing-microorganism in Tropical soils with nitrification inhibitor. *Sci. Agric.* 77:2020. 10.1590/1678-992X-2018-0370

[B21] Bastani M. Harter T. (2019). Source area management practices as remediation tool to address groundwater nitrate pollution in drinking supply wells. *J. Contam. Hydrol.* 226:103521. 10.1016/J.JCONHYD.2019.103521 31330339

[B22] Bayu T. (2020). Review on contribution of integrated soil fertility management for climate change mitigation and agricultural sustainability. *Cogent Environ. Sci.* 6:1823631. 10.1080/23311843.2020.1823631

[B23] Behera S. N. Sharma M. Aneja V. P. Balasubramanian R. (2013). Ammonia in the atmosphere: a review on emission sources, atmospheric chemistry and deposition on terrestrial bodies. *Environ. Sci. Pollut. Res.* 2011 8092–8131. 10.1007/S11356-013-2051-9 23982822

[B24] Beier M. P. Fujita T. Sasaki K. Kanno K. Ohashi M. Tamura W. (2019). The urea transporter DUR3 contributes to rice production under nitrogen-deficient and field conditions. *Physiol. Plant.* 167 75–89. 10.1111/PPL.12872 30426495

[B25] Belder P. Bouman B. A. M. Spiertz J. H. J. Peng S. Castañeda A. R. Visperas R. M. (2005). Crop performance, nitrogen and water use in flooded and aerobic rice. *Plant Soil* 273 167–182. 10.1007/s11104-004-7401-4

[B26] Belder P. Bouman B. A. Cabangon R. Guoan L. Quilang E. J. Yuanhua L. (2004). Effect of water-saving irrigation on rice yield and water use in typical lowland conditions in Asia. *Agric. Water Manag.* 65 193–210. 10.1016/J.AGWAT.2003.09.002

[B27] Bernaola L. Cange G. Way M. O. Gore J. Hardke J. Stout M. (2018). Natural Colonization of Rice by Arbuscular Mycorrhizal Fungi in Different Production Areas. *Rice Sci.* 25 169–174. 10.1016/J.RSCI.2018.02.006

[B28] Beule L. Vaupel A. Moran-Rodas V. E. (2022). Abundance, Diversity, and Function of Soil Microorganisms in Temperate Alley-Cropping Agroforestry Systems: A Review. *Microorganisms* 10:616. 10.3390/microorganisms10030616 35336196 PMC8953468

[B29] Bhuyan M. H. M. Ferdousi M. R. Iqbal M. T. (2012). Yield and Growth Response to Transplanted Aman Rice under Raised Bed over Conventional Cultivation Method. *ISRN Agron.* 2012 1–8. 10.5402/2012/646859

[B30] Bijay-Singh Craswell E. (2021). Fertilizers and nitrate pollution of surface and ground water: an increasingly pervasive global problem. *SN Appl. Sci.* 3:518. 10.1007/S42452-021-04521-8

[B31] Bishwajit G. Sarker S. Kpoghomou M. A. Gao H. Jun L. Yin D. (2013). Self-sufficiency in rice and food security: A South Asian perspective. *Agric. Food Secur.* 2 1–6. 10.1186/2048-7010-2-10/TABLES/1

[B32] Black E. M. Chimenti M. S. Just C. L. (2019). Metagenomic analysis of nitrogen-cycling genes in upper Mississippi river sediment with mussel assemblages. *Microbiologyopen* 8:e00739. 10.1002/mbo3.739 30270525 PMC6528593

[B33] Bueno E. Mania D. Frostegard Ç Bedmar E. J. Bakken L. R. Delgado M. J. (2015). Anoxic growth of Ensifer meliloti 1021 by N2O-reduction, a potential mitigation strategy. *Front. Microbiol.* 6:537. 10.3389/fmicb.2015.00537 26074913 PMC4443521

[B34] Butterbach-Bahl K. Baggs E. M. Dannenmann M. Kiese R. Zechmeister-Boltenstern S. (2013). Nitrous oxide emissions from soils: how well do we understand the processes and their controls? *Philos. Trans. R. Soc. B Biol. Sci.* 368:20130122. 10.1098/RSTB.2013.0122 23713120 PMC3682742

[B35] Cameron K. C. Di H. J. Moir J. L. (2013). Nitrogen losses from the soil/plant system: a review. *Ann. Appl. Biol.* 162 145–173. 10.1111/AAB.12014

[B36] Cao P. Sun W. Huang Y. Yang J. Yang K. Lv C. (2020). Effects of Elevated CO2 Concentration and Nitrogen Application Levels on the Accumulation and Translocation of Non-Structural Carbohydrates in Japonica Rice. *Sustainability* 12:5386. 10.3390/SU12135386

[B37] Carvalho S. D. Castillo J. A. (2018). Influence of Light on Plant–Phyllosphere Interaction. *Front. Plant Sci.* 9:1482. 10.3389/FPLS.2018.01482/BIBTEX 30369938 PMC6194327

[B38] Castillo J. Kirk G. J. D. Rivero M. J. Dobermann A. Haefele S. M. (2021). The nitrogen economy of rice-livestock systems in Uruguay. *Glob. Food Sec.* 30:100566. 10.1016/J.GFS.2021.100566

[B39] Cataldo E. Salvi L. Paoli F. Fucile M. Masciandaro G. Manzi D. (2021). Application of zeolites in agriculture and other potential uses: A review. *Agronomy* 11:1547. 10.3390/agronomy11081547

[B40] Chalk P. Smith C. (2021). On inorganic N uptake by vascular plants: Can 15N tracer techniques resolve the NH4+ versus NO3- “preference” conundrum? *Eur. J. Soil Sci.* 72 1762–1779. 10.1111/EJSS.13069

[B41] Chamizo S. Mugnai G. Rossi F. Certini G. De Philippis R. (2018). Cyanobacteria inoculation improves soil stability and fertility on different textured soils: Gaining insights for applicability in soil restoration. *Front. Environ. Sci.* 6:49. 10.3389/FENVS.2018.00049/BIBTEX

[B42] Chang J. Havlík P. Leclère D. de Vries W. Valin H. Deppermann A. (2021a). Reconciling regional nitrogen boundaries with global food security. *Nat. Food* 29 700–711. 10.1038/s43016-021-00366-x 37117470

[B43] Chang J. Sun Y. Tian L. Ji L. Luo S. Nasir F. (2021b). The Structure of Rhizosphere Fungal Communities of Wild and Domesticated Rice: Changes in Diversity and Co-occurrence Patterns. *Front. Microbiol.* 12:45. 10.3389/FMICB.2021.610823/BIBTEX PMC7890246 33613482

[B44] Chapagain T. Riseman A. Yamaji E. (2011). Assessment of System of Rice Intensification (SRI) and Conventional Practices under Organic and Inorganic Management in Japan. *Rice Sci.* 18 311–320. 10.1016/S1672-6308(12)60010-9

[B45] Checcucci A. Marchetti M. (2020). The Rhizosphere Talk Show: The Rhizobia on Stage. *Front. Agron.* 2:25. 10.3389/FAGRO.2020.591494/BIBTEX

[B46] Chen C. Wang E. Yu Q. Zhang Y. (2009). Quantifying the effects of climate trends in the past 43 years (1961–2003) on crop growth and water demand in the North China Plain. *Clim. Chang.* 1003 559–578. 10.1007/S10584-009-9690-3

[B47] Chen G. Liu H. Zhang J. Liu P. Dong S. (2012). Factors affecting summer maize yield under climate change in Shandong Province in the Huanghuaihai Region of China. *Int. J. Biometeorol.* 56 621–629. 10.1007/s00484-011-0460-3 21688211

[B48] Chen J. Liu X. Liu S. Fan X. Zhao L. Song M. (2020). Co-Overexpression of OsNAR2.1 and OsNRT2.3a Increased Agronomic Nitrogen Use Efficiency in Transgenic Rice Plants. *Front. Plant Sci.* 11:1245. 10.3389/FPLS.2020.01245/BIBTEX 32903417 PMC7434940

[B49] Chen G. Zhao G. Cheng W. Zhang H. Lu C. Zhang H. (2020). Rice nitrogen use efficiency does not link to ammonia volatilization in paddy fields. *Sci. Total Environ.* 741:140433. 10.1016/J.SCITOTENV.2020.140433 32610240

[B50] Chen H. Jin W. Liang Z. Abomohra A. E. F. Zhou X. Tu R. (2017). Abundance and diversity of ammonia-oxidizing archaea in a biological aerated filter process. *Ann. Microbiol.* 67 405–416. 10.1007/s13213-017-1272-4

[B51] Chen J. Zhang Y. Tan Y. Zhang M. Zhu L. Xu G. (2016). Agronomic nitrogen-use efficiency of rice can be increased by driving OsNRT2.1 expression with the OsNAR2.1 promoter. *Plant Biotechnol. J.* 14 1705–1715. 10.1111/PBI.12531 26826052 PMC5066696

[B52] Chen Y. L. Hu H. W. Han H. Y. Du Y. Wan S. Q. Xu Z. W. (2014). Abundance and community structure of ammonia-oxidizing Archaea and Bacteria in response to fertilization and mowing in a temperate steppe in Inner Mongolia. *FEMS Microbiol. Ecol.* 89 67–79. 10.1111/1574-6941.12336 24712910

[B53] Chivenge P. Zingore S. Ezui K. S. Njoroge S. Bunquin M. A. Dobermann A. (2022). Progress in research on site-specific nutrient management for smallholder farmers in sub-Saharan Africa. *F. Crop. Res.* 281:108503. 10.1016/J.FCR.2022.108503 35582149 PMC8935389

[B54] Chu Q. Xu S. Xue L. Liu Y. Feng Y. Yu S. (2020). Bentonite hydrochar composites mitigate ammonia volatilization from paddy soil and improve nitrogen use efficiency. *Sci. Total Environ.* 718:137301. 10.1016/J.SCITOTENV.2020.137301 32105922

[B55] Congreves K. A. Otchere O. Ferland D. Farzadfar S. Williams S. Arcand M. M. (2021). Nitrogen Use Efficiency Definitions of Today and Tomorrow. *Front. Plant Sci.* 12:912. 10.3389/FPLS.2021.637108/BIBTEX PMC8220819 34177975

[B56] Coskun D. Britto D. T. Shi W. Kronzucker H. J. (2017). Nitrogen transformations in modern agriculture and the role of biological nitrification inhibition. *Nat. Plants* 3:17074. 10.1038/nplants.2017.74 28585561

[B57] Cui Y. Zhang W. Lin X. Xu S. Xu J. Li Z. (2018). Simultaneous Improvement and Genetic Dissection of Drought Tolerance Using Selected Breeding Populations of Rice. *Front. Plant Sci.* 9:320. 10.3389/fpls.2018.00320 29599789 PMC5862857

[B58] Czyżewski B. Smędzik-Ambroży K. Mrówczyńska-Kamińska A. (2020). Impact of environmental policy on eco-efficiency in country districts in Poland: How does the decreasing return to scale change perspectives? *Environ. Impact Assess. Rev.* 84:106431. 10.1016/J.EIAR.2020.106431

[B59] Dastagir M. R. (2015). Modeling recent climate change induced extreme events in Bangladesh: A review. *Weather Clim. Extrem.* 7 49–60. 10.1016/J.WACE.2014.10.003

[B60] Davidson H. Shrestha R. Cornulier T. Douglas A. Travis T. Johnson D. (2019). Spatial effects and GWA mapping of root colonization assessed in the interaction between the rice diversity panel 1 and an arbuscular mycorrhizal fungus. *Front. Plant Sci.* 10:633. 10.3389/FPLS.2019.00633/BIBTEX 31156686 PMC6533530

[B61] de Bang T. C. Husted S. Laursen K. H. Persson D. P. Schjoerring J. K. (2021). The molecular–physiological functions of mineral macronutrients and their consequences for deficiency symptoms in plants. *New Phytol.* 229 2446–2469. 10.1111/NPH.17074 33175410

[B62] De Gannes V. Eudoxie G. Hickey W. J. (2014). Impacts of Edaphic Factors on Communities of Ammonia-Oxidizing Archaea, Ammonia-Oxidizing Bacteria and Nitrification in Tropical Soils. *PLoS One* 9:e89568. 10.1371/JOURNAL.PONE.0089568 24586878 PMC3938500

[B63] Dellero Y. (2020). Manipulating Amino Acid Metabolism to Improve Crop Nitrogen Use Efficiency for a Sustainable Agriculture. *Front. Plant Sci.* 11:1857. 10.3389/FPLS.2020.602548/BIBTEX PMC7733991 33329673

[B64] Deng J. Harrison M. T. Liu K. Ye J. Xiong X. Fahad S. (2022). Integrated Crop Management Practices Improve Grain Yield and Resource Use Efficiency of Super Hybrid Rice. *Front. Plant Sci.* 13:851562. 10.3389/FPLS.2022.851562 35432400 PMC9007698

[B65] Devkota K. P. Manschadi A. M. Lamers J. P. A. Humphreys E. Devkota M. Egamberdiev O. (2013). Growth and yield of rice (Oryza sativa L.) under resource conservation technologies in the irrigated drylands of Central Asia. *F. Crop. Res.* 149 115–126. 10.1016/j.fcr.2013.04.015

[B66] Devkota N. Paija N. (2020). Impact of Climate Change on Paddy Production: Evidence from Nepal. *Asian J. Agric. Dev.* 17 63–78. 10.37801/AJAD2020.17.2.4

[B67] Ding J. Ma M. Jiang X. Liu Y. Zhang J. Suo L. (2020). Effects of applying inorganic fertilizer and organic manure for 35 years on the structure and diversity of ammonia-oxidizing archaea communities in a Chinese Mollisols field. *Microbiologyopen* 9:e00942. 10.1002/MBO3.942 31568679 PMC6957403

[B68] Ding L. J. Cui H. L. Nie S. A. Long X. E. Duan G. L. Zhu Y. G. (2019). Microbiomes inhabiting rice roots and rhizosphere. *FEMS Microbiol. Ecol* 95:fiz040. 10.1093/FEMSEC/FIZ040 30916760

[B69] Dorich C. D. Conant R. T. Albanito F. Butterbach-Bahl K. Grace P. Scheer C. (2020). Improving N2O emission estimates with the global N2O database. *Curr. Opin. Environ. Sustain.* 47 13–20. 10.1016/J.COSUST.2020.04.006

[B70] Driedonks N. Rieu I. Vriezen W. H. (2016). Breeding for plant heat tolerance at vegetative and reproductive stages. *Plant Reprod.* 291 67–79. 10.1007/S00497-016-0275-9 26874710 PMC4909801

[B71] Drury C. F. Reynolds W. D. Yang X. M. McLaughlin N. B. Welacky T. W. Calder W. (2012). Nitrogen Source, Application Time, and Tillage Effects on Soil Nitrous Oxide Emissions and Corn Grain Yields. *Soil Sci. Soc. Am. J.* 76 1268–1279. 10.2136/SSSAJ2011.0249

[B72] Dybowski D. Dzierzbicka-Glowacka L. A. Pietrzak S. Juszkowska D. Puszkarczuk T. (2020). Estimation of nitrogen leaching load from agricultural fields in the Puck Commune with an interactive calculator. *PeerJ* 2020:e8899. 10.7717/PEERJ.8899/SUPP-3 PMC7104721 32257651

[B73] Ebbisa A. (2022). “Toward the Recent Advances in Nutrient Use Efficiency (NUE): Strategies to Improve Phosphorus Availability to Plants,” in *Sustainable Crop Production - Recent Advances* (eds), Choudhary M. Yadav R. P. Meena S. K. Meena V. (London: IntechOpen), 10.5772/INTECHOPEN.102595

[B74] Edwards J. A. Santos-Medellín C. M. Liechty Z. S. Nguyen B. Lurie E. Eason S. (2018). Compositional shifts in root-associated bacterial and archaeal microbiota track the plant life cycle in field-grown rice. *PLoS Biol.* 16:e2003862. 10.1371/JOURNAL.PBIO.2003862 29474469 PMC5841827

[B75] EL Sabagh A. Islam M. S. Skalicky M. Ali Raza M. Singh K. Anwar Hossain M. (2021). Salinity Stress in Wheat (*Triticum aestivum* L.) in the Changing Climate: Adaptation and Management Strategies. *Front. Agron.* 3:43. 10.3389/FAGRO.2021.661932/BIBTEX

[B76] Elbasiouny H. Elbehiry F. (2020). “Rice Production in Egypt: The Challenges of Climate Change and Water Deficiency,” in *Climate Change Impacts on Agriculture and Food Security in Egypt*, eds Negm A. M. Omran E. S. E. (Berlin: Springer Nature), 295–319. 10.1007/978-3-030-41629-4_14/COVER

[B77] Erisman J. W. Leach A. Bleeker A. Atwell B. Cattaneo L. Galloway J. (2018). An integrated approach to a nitrogen use efficiency (NUE) indicator for the food production-consumption chain. *Sustainability* 10:925. 10.3390/su10040925

[B78] Fahad S. Bajwa A. A. Nazir U. Anjum S. A. Farooq A. Zohaib A. (2017). Crop production under drought and heat stress: Plant responses and management options. *Front. Plant Sci.* 8:1147. 10.3389/FPLS.2017.01147/BIBTEX 28706531 PMC5489704

[B79] Fan X. Chen Z. Niu Z. Zeng R. Ou J. Liu X. (2021). Replacing Synthetic Nitrogen Fertilizer with Different Types of Organic Materials Improves Grain Yield in China: A Meta-Analysis. *Agronomy* 11:2429. 10.3390/AGRONOMY11122429/S1

[B80] FAO (2016). *OECD-FAO Agricultural Outlook 2017-2026 SpECiAl FOCuS: SOuthEASt ASiA.* Rome: FAO.

[B81] Farooq M. S. Gyilbag A. Virk A. L. Xu Y. (2021). Adaptability mechanisms of japonica rice based on the comparative temperature conditions of harbin and qiqihar, heilongjiang province of Northeast China. *Agronomy* 11:2367. 10.3390/agronomy11112367

[B82] Farooq M. S. Uzair M. Raza A. Habib M. Xu Y. Yousuf M. (2022b). Uncovering the Research Gaps to Alleviate the Negative Impacts of Climate Change on Food Security: A Review. *Front. Plant Sci.* 13:927535. 10.3389/fpls.2022.927535 35903229 PMC9315450

[B83] Farooq M. S. Uzair M. Maqbool Z. Fiaz S. Yousuf M. Yang S. H. (2022a). Improving Nitrogen Use Efficiency in Aerobic Rice Based on Insights Into the Ecophysiology of Archaeal and Bacterial Ammonia Oxidizers. *Front. Plant Sci.* 13:913204. 10.3389/fpls.2022.913204 35769304 PMC9234532

[B84] Farzadfar S. Knight J. D. Congreves K. A. (2021). Soil organic nitrogen: an overlooked but potentially significant contribution to crop nutrition. *Plant Soil* 462 7–23. 10.1007/S11104-021-04860-W 34720208 PMC8550315

[B85] Fatima Z. Ahmed M. Hussain M. Abbas G. Ul-Allah S. Ahmad S. (2020). The fingerprints of climate warming on cereal crops phenology and adaptation options. *Sci. Rep.* 10:18013. 10.1038/s41598-020-74740-3 33093541 PMC7581754

[B86] Frostegård Å Vick S. H. W. Lim N. Y. N. Bakken L. R. Shapleigh J. P. (2021). Linking meta-omics to the kinetics of denitrification intermediates reveals pH-dependent causes of N2O emissions and nitrite accumulation in soil. *ISME J.* 16 26–37. 10.1038/s41396-021-01045-2 34211102 PMC8692524

[B87] Fuhrmann I. He Y. Lehndorff E. Brüggemann N. Amelung W. Wassmann R. (2018). Nitrogen fertilizer fate after introducing maize and upland-rice into continuous paddy rice cropping systems. *Agric. Ecosyst. Environ.* 258 162–171. 10.1016/J.AGEE.2018.02.021

[B88] Fukase E. Martin W. (2017). Economic Growth, Convergence, and World Food Demand and Supply. *World Dev.* 132:104954.

[B89] Galloway J. N. Townsend A. R. Erisman J. W. Bekunda M. Cai Z. Freney J. R. (2008). Transformation of the nitrogen cycle: Recent trends, questions, and potential solutions. *Science (80-.).* 320 889–892. 10.1126/SCIENCE.1136674/SUPPL_FILE/GALLOWAY_SOM.PDF 18487183

[B90] Geng Y. Cao G. Wang L. Wang S. (2019). Effects of equal chemical fertilizer substitutions with organic manure on yield, dry matter, and nitrogen uptake of spring maize and soil nitrogen distribution. *PLoS One* 14:e0219512. 10.1371/JOURNAL.PONE.0219512 31287845 PMC6615609

[B91] Giordano M. Petropoulos S. A. Rouphael Y. (2021). The fate of nitrogen from soil to plants: Influence of agricultural practices in modern agriculture. *Agriculture* 11 1–22. 10.3390/agriculture11100944

[B92] Gołaś M. Sulewski P. Was A. Pogodzińska K. Kłoczko-Gajewska A. (2020). On the way to sustainable agriculture—eco-efficiency of polish commercial farms. *Agriculture* 10 1–24. 10.3390/agriculture10100438

[B93] Groenveld T. Lazarovitch N. Kohn Y. Y. Gelfand I. (2020). Environmental Tradeoffs between Nutrient Recycling and Greenhouse Gases Emissions in an Integrated Aquaculture-Agriculture System. *Environ. Sci. Technol.* 54 9584–9592. 10.1021/ACS.EST.0C00869/ASSET/IMAGES/LARGE/ES0C00869_0005.JPEG 32790417 PMC7460073

[B94] Grzyb A. Wolna-Maruwka A. Niewiadomska A. (2021). The Significance of Microbial Transformation of Nitrogen Compounds in the Light of Integrated Crop Management. *Agronomy* 11:1415. 10.3390/AGRONOMY11071415

[B95] Guardia G. García-Gutiérrez S. Rodríguez-Pérez R. Recio J. Vallejo A. (2021). Increasing N use efficiency while decreasing gaseous N losses in a non-tilled wheat (Triticum aestivum L.) crop using a double inhibitor. *Agric. Ecosyst. Environ.* 319:107546. 10.1016/j.agee.2021.107546

[B96] Guo H. Ma L. Liang Y. Hou Z. Min W. (2020). Response of ammonia-oxidizing Bacteria and Archaea to long-term saline water irrigation in alluvial grey desert soils. *Sci. Rep.* 10:489. 10.1038/s41598-019-57402-x 31949227 PMC6965641

[B97] Guo S. Yan T. Zhai L. Yen H. Liu J. Li W. (2022). Nitrogen Transport/Deposition from Paddy Ecosystem and Potential Pollution Risk Period in Southwest China. *Water* 14:539. 10.3390/W14040539

[B98] Gweyi-Onyango J. P. Ntinyari W. Ogollaegesa A. Mose R. Njinju S. Giweta M. (2021). Differences in seasons and rice varieties provide opportunities for improving nitrogen use efficiency and management in irrigated rice in Kenya. *Environ. Res. Lett.* 16:075003. 10.1088/1748-9326/AC03DD

[B99] Habbib H. Hirel B. Spicher F. Dubois F. Tétu T. (2019). In Winter Wheat (Triticum Aestivum L.), No-Till Improves Photosynthetic Nitrogen and Water-Use Efficiency. *J. Crop Sci. Biotechnol.* 231 39–46. 10.1007/S12892-019-0122-0

[B100] Hakeem K. R. Ahmad A. Iqbal M. Gucel S. Ozturk M. (2011). Nitrogen-efficient rice cultivars can reduce nitrate pollution. *Environ. Sci. Pollut. Res.* 18 1184–1193. 10.1007/S11356-010-0434-8 21359512

[B101] Hakeem K. R. Sabir M. Ozturk M. Akhtar M. S. Ibrahim F. H. Ashraf M. (2016). Nitrate and Nitrogen Oxides: Sources, Health Effects and Their Remediation. *Rev. Environ. Contam. Toxicol.* 242 183–217. 10.1007/398_2016_11 27734212

[B102] Hall A. J. Richards R. A. (2013). Prognosis for genetic improvement of yield potential and water-limited yield of major grain crops. *F. Crop. Res.* 143 18–33. 10.1016/J.FCR.2012.05.014

[B103] Han S. Li X. Luo X. Wen S. Chen W. Huang Q. (2018). Nitrite-oxidizing bacteria community composition and diversity are influenced by fertilizer regimes, but are independent of the soil aggregate in acidic subtropical red soil. *Front. Microbiol.* 9:885. 10.3389/FMICB.2018.00885/BIBTEX 29867799 PMC5951965

[B104] Han Z. Walter M. T. Drinkwater L. E. (2017). N2O emissions from grain cropping systems: a meta-analysis of the impacts of fertilizer-based and ecologically-based nutrient management strategies. *Nutr. Cycl. Agroecosyst.* 107 335–355. 10.1007/S10705-017-9836-Z

[B105] Harman G. Khadka R. Doni F. Uphoff N. (2021). Benefits to Plant Health and Productivity From Enhancing Plant Microbial Symbionts. *Front. Plant Sci.* 11:2001. 10.3389/FPLS.2020.610065/BIBTEX PMC8072474 33912198

[B106] Hassan M. U. Aamer M. Mahmood A. Awan M. I. Barbanti L. Seleiman M. F. (2022). Management Strategies to Mitigate N2O Emissions in Agriculture. *Life* 12:439. 10.3390/LIFE12030439 35330190 PMC8949344

[B107] Hayatsu M. Katsuyama C. Tago K. (2021). Overview of recent researches on nitrifying microorganisms in soil. *Soil Sci. Plant Nutr.* 67 619–632. 10.1080/00380768.2021.1981119

[B108] Hayatsu M. Tago K. Uchiyama I. Toyoda A. Wang Y. Shimomura Y. (2017). An acid-tolerant ammonia-oxidizing γ-proteobacterium from soil. *ISME J.* 115 1130–1141. 10.1038/ismej.2016.191 28072419 PMC5437925

[B109] He H. Jansson P. E. Svensson M. Meyer A. Klemedtsson L. Kasimir Å (2016). Factors controlling Nitrous Oxide emission from a spruce forest ecosystem on drained organic soil, derived using the CoupModel. *Ecol. Modell.* 321 46–63. 10.1016/J.ECOLMODEL.2015.10.030

[B110] He H. Zhen Y. Mi T. Fu L. Yu Z. (2018). Ammonia-oxidizing archaea and bacteria differentially contribute to ammonia oxidation in sediments from adjacent waters of Rushan Bay, China. *Front. Microbiol.* 9:116. 10.3389/FMICB.2018.00116/BIBTEX 29456526 PMC5801408

[B111] He X. Li S. Wu F. (2021). Responses of ammonia-oxidizing microorganisms to intercropping systems in different seasons. *Agriculture* 11:195. 10.3390/agriculture11030195

[B112] Hernández-Guzmán M. Pérez-Hernández V. Navarro-Noya Y. E. Luna-Guido M. L. Verhulst N. Govaerts B. (2022). Application of ammonium to a N limited arable soil enriches a succession of bacteria typically found in the rhizosphere. *Sci. Rep.* 12:4110. 10.1038/s41598-022-07623-4 35260645 PMC8904580

[B113] Hess L. J. T. Hinckley E. L. S. Robertson G. P. Matson P. A. (2020). Rainfall intensification increases nitrate leaching from tilled but not no-till cropping systems in the U.S. Midwest. *Agric. Ecosyst. Environ.* 290:106747. 10.1016/j.agee.2019.106747

[B114] Hirzel J. Matus I. (2013). Effect of soil depth and increasing fertilization rate on yield and its components of two durum wheat varieties. *Chil. J. Agric. Res.* 73 55–59. 10.4067/S0718-58392013000100008 27315006

[B115] Hoffman F. M. Randerson J. T. Arora V. K. Bao Q. Cadule P. Ji D. (2014). Causes and implications of persistent atmospheric carbon dioxide biases in Earth System Models. *J. Geophys. Res. Biogeosci.* 119 141–162. 10.1002/2013JG002381

[B116] Horie T. (2019). Global warming and rice production in Asia: Modeling, impact prediction and adaptation. *Proc. Jpn. Acad. Ser. B. Phys. Biol. Sci.* 95:211. 10.2183/PJAB.95.016 31189777 PMC6751296

[B117] Hornsey M. J. Harris E. A. Bain P. G. Fielding K. S. (2016). Meta-analyses of the determinants and outcomes of belief in climate change. *Nat. Clim. Chang.* 66 622–626. 10.1038/nclimate2943

[B118] Howell K. R. Shrestha P. Dodd I. C. (2015). Alternate wetting and drying irrigation maintained rice yields despite half the irrigation volume, but is currently unlikely to be adopted by smallholder lowland rice farmers in Nepal. *Food Energy Secur.* 4:144. 10.1002/FES3.58 27610231 PMC4998133

[B119] Hu G. Liu X. He H. Zhang W. Xie H. Wu Y. (2015). Multi-Seasonal Nitrogen Recoveries from Crop Residue in Soil and Crop in a Temperate Agro-Ecosystem. *PLoS One* 10:e0133437. 10.1371/JOURNAL.PONE.0133437 26192436 PMC4507866

[B120] Hu H. W. He J. Z. Singh B. K. (2017). Harnessing microbiome-based biotechnologies for sustainable mitigation of nitrous oxide emissions. *Microb. Biotechnol.* 10 1226–1231. 10.1111/1751-7915.12758 28696064 PMC5609469

[B121] Hu Y. Fan L. Liu Z. Yu Q. Liang S. Chen S. (2019). Rice production and climate change in Northeast China: evidence of adaptation through land use shifts. *Environ. Res. Lett.* 14:024014. 10.1088/1748-9326/AAFA55

[B122] Huang J. Duan Y. Hua X. M. Gang Z. L. Mei Z. X. Bo W. (2017). Nitrogen mobility, ammonia volatilization, and estimated leaching loss from long-term manure incorporation in red soil. *J. Integr. Agric.* 16 2082–2092. 10.1016/S2095-3119(16)61498-3

[B123] Huang T. Ju X. Yang H. (2017). Nitrate leaching in a winter wheat-summer maize rotation on a calcareous soil as affected by nitrogen and straw management. *Sci. Rep.* 71:42247. 10.1038/srep42247 28176865 PMC5296732

[B124] Huang M. Fan L. Chen J. Jiang L. Zou Y. (2018). Continuous applications of biochar to rice: Effects on nitrogen uptake and utilization. *Sci. Rep.* 8:11461. 10.1038/s41598-018-29877-7 30061619 PMC6065394

[B125] Huang S. Lv W. Bloszies S. Shi Q. Pan X. Zeng Y. (2016). Effects of fertilizer management practices on yield-scaled ammonia emissions from croplands in China: A meta-analysis. *F. Crop. Res.* 192 118–125. 10.1016/J.FCR.2016.04.023

[B126] Huang T. Gao B. Hu X. K. Lu X. Well R. Christie P. (2014). Ammonia-oxidation as an engine to generate nitrous oxide in an intensively managed calcareous Fluvo-aquic soil. *Sci. Rep.* 41:3950. 10.1038/srep03950 24492201 PMC3912618

[B127] Huang Y. Gerber S. (2015). Global soil nitrous oxide emissions in a dynamic carbon-nitrogen model. *Biogeosciences* 12, 6405–6427.

[B128] Huérfano X. Estavillo J. M. Torralbo F. Vega-Mas I. González-Murua C. Fuertes-Mendizábal T. (2022). Dimethylpyrazole-based nitrification inhibitors have a dual role in N2O emissions mitigation in forage systems under Atlantic climate conditions. *Sci. Total Environ.* 807:150670. 10.1016/J.SCITOTENV.2021.150670 34610408

[B129] Hulmani S. Salakinkop S. R. Somangouda G. (2022). Productivity, nutrient use efficiency, energetic, and economics of winter maize in south India. *PLoS One* 17:e0266886. 10.1371/JOURNAL.PONE.0266886 35862389 PMC9302768

[B130] Hwalla N. El Labban S. Bahn R. A. (2016). Nutrition security is an integral component of food security. *Front. Life Sci.* 9 167–172. 10.1080/21553769.2016.1209133

[B131] Ierna A. Mauromicale G. (2019). Sustainable and Profitable Nitrogen Fertilization Management of Potato. *Agronomy* 9:582. 10.3390/AGRONOMY9100582

[B132] IPCC. (2021). *Climate Change 2021 The Physical Science Basis Summary for Policymakers Working Group I Contribution to the Sixth Assessment Report of the Intergovernmental Panel on Climate Change.* Geneva: IPCC.

[B133] Iqbal A. Dong Q. Wang X. Gui H. Zhang H. Zhang X. (2020a). Variations in Nitrogen Metabolism are Closely Linked with Nitrogen Uptake and Utilization Efficiency in Cotton Genotypes under Various Nitrogen Supplies. *Plants* 9:250. 10.3390/PLANTS9020250 32075340 PMC7076418

[B134] Iqbal A. Qiang D. Alamzeb M. Xiangru W. Huiping G. Hengheng Z. (2020b). Untangling the molecular mechanisms and functions of nitrate to improve nitrogen use efficiency. *J. Sci. Food Agric.* 100 904–914. 10.1002/JSFA.10085 31612486

[B135] Ishii S. Ikeda S. Minamisawa K. Senoo K. (2011). Nitrogen cycling in rice paddy environments: Past achievements and future challenges. *Microbes Environ.* 26 282–292. 10.1264/jsme2.ME11293 22008507

[B136] Ishii S. Yamamoto M. Kikuchi M. Oshima K. Hattori M. Otsuka S. (2009). Microbial populations responsive to denitrification-inducing conditions in rice paddy soil, as revealed by comparative 16S rRNA gene analysis. *Appl. Environ. Microbiol.* 75 7070–7078. 10.1128/AEM.01481-09 19767468 PMC2786546

[B137] Islam S. F. Sander B. O. Quilty J. R. de Neergaard A. van Groenigen J. W. Jensen L. S. (2020). Mitigation of greenhouse gas emissions and reduced irrigation water use in rice production through water-saving irrigation scheduling, reduced tillage and fertiliser application strategies. *Sci. Total Environ.* 739:140215. 10.1016/J.SCITOTENV.2020.140215 32758960

[B138] Islam S. N. Winkel J. (2017). *Climate Change and Social Inequality *.* Available online at: http://www.ejnetindiaresource.org/ejissues/bali.pdfen- (accessed on Apr 18, 2022).

[B139] Jacoby R. Peukert M. Succurro A. Koprivova A. Kopriva S. (2017). The role of soil microorganisms in plant mineral nutrition—current knowledge and future directions. *Front. Plant Sci.* 8:1617. 10.3389/FPLS.2017.01617/BIBTEX 28974956 PMC5610682

[B140] Jamil F. Mukhtar H. Fouillaud M. Dufossé L. (2022). Rhizosphere Signaling: Insights into Plant–Rhizomicrobiome Interactions for Sustainable Agronomy. *Microorganisms* 10:899. 10.3390/MICROORGANISMS10050899 35630345 PMC9147336

[B141] Jang J. Anderson E. L. Venterea R. T. Sadowsky M. J. Rosen C. J. Feyereisen G. W. (2019). Denitrifying Bacteria Active in Woodchip Bioreactors at Low-Temperature Conditions. *Front. Microbiol.* 10:635. 10.3389/fmicb.2019.00635 31001220 PMC6454037

[B142] Jankowski K. J. Neill C. Davidson E. A. Macedo M. N. Costa C. Galford G. L. (2018). Deep soils modify environmental consequences of increased nitrogen fertilizer use in intensifying Amazon agriculture. *Sci. Rep.* 8:13478. 10.1038/S41598-018-31175-1 30194382 PMC6128839

[B143] Jia D. Dai X. Xie Y. He M. (2021). Alternate furrow irrigation improves grain yield and nitrogen use efficiency in winter wheat. *Agric. Water Manag.* 244:106606. 10.1016/J.AGWAT.2020.106606

[B144] Jia Y. Wang J. Qu Z. Zou D. Sha H. Liu H. (2019). Effects of low water temperature during reproductive growth on photosynthetic production and nitrogen accumulation in rice. *F. Crop. Res.* 242:107587. 10.1016/J.FCR.2019.107587

[B145] Jiang H. Huang L. Deng Y. Wang S. Zhou Y. Liu L. (2014). Latitudinal Distribution of Ammonia-Oxidizing Bacteria and Archaea in the Agricultural Soils of Eastern China. *Appl. Environ. Microbiol.* 80:5593. 10.1128/AEM.01617-14 25002421 PMC4178612

[B146] Jiao X. Maimaitiyiming A. Salahou M. K. Liu K. Guo W. (2017). Impact of Groundwater Level on Nitrate Nitrogen Accumulation in the Vadose Zone Beneath a Cotton Field. *Water* 9:171. 10.3390/W9030171

[B147] Johnston A. M. Bruulsema T. W. (2014). 4R Nutrient Stewardship for Improved Nutrient Use Efficiency. *Procedia Eng.* 83 365–370. 10.1016/J.PROENG.2014.09.029

[B148] Joshi R. Singh B. Shukla A. (2018). Evaluation of elite rice genotypes for physiological and yield attributes under aerobic and irrigated conditions in tarai areas of western Himalayan region. *Curr. Plant Biol.* 13 45–52. 10.1016/J.CPB.2018.05.001

[B149] Ju X. T. Zhang C. (2017). Nitrogen cycling and environmental impacts in upland agricultural soils in North China: A review. *J. Integr. Agric* 16 2848–2862. 10.1016/S2095-3119(17)61743-X

[B150] Jung M. Y. Park S. J. Min D. Kim J. S. Rijpstra W. I. C. Damsté J. S. S. (2011). Enrichment and Characterization of an Autotrophic Ammonia-Oxidizing Archaeon of Mesophilic Crenarchaeal Group I.1a from an Agricultural Soil. *Appl. Environ. Microbiol.* 77:8635. 10.1128/AEM.05787-11 22003023 PMC3233086

[B151] Kadiyala M. D. M. Mylavarapu R. S. Li Y. C. Reddy G. B. Reddy M. D. (2012). Impact of aerobic rice cultivation on growth, yield, and water productivity of rice-maize rotation in semiarid tropics. *Agron. J.* 104 1757–1765. 10.2134/agronj2012.0148

[B152] Kang S. Eltahir E. A. B. (2018). North China Plain threatened by deadly heatwaves due to climate change and irrigation. *Nat. Commun.* 91:2894. 10.1038/s41467-018-05252-y 30065269 PMC6068174

[B153] Kang Y. Liu M. Song Y. Huang X. Yao H. Cai X. (2016). High-resolution ammonia emissions inventories in China from 1980 to 2012. *Atmos. Chem. Phys.* 16 2043–2058. 10.5194/ACP-16-2043-2016

[B154] Kaur G. Singh G. Motavalli P. P. Nelson K. A. Orlowski J. M. Golden B. R. (2020). Impacts and management strategies for crop production in waterlogged or flooded soils: A review. *Agron. J.* 112 1475–1501. 10.1002/AGJ2.20093

[B155] Ke X. Lu Y. (2012). Adaptation of ammonia-oxidizing microorganisms to environment shift of paddy field soil. *FEMS Microbiol. Ecol.* 80 87–97. 10.1111/J.1574-6941.2011.01271.X 22145990

[B156] Keating B. A. Carberry P. S. Bindraban P. S. Asseng S. Meinke H. Dixon J. (2010). Eco-efficient agriculture: Concepts, Challenges, And opportunities. *Crop Sci.* 50 S–109–S–119. 10.2135/cropsci2009.10.0594

[B157] Kerou M. Offre P. Valledor L. Abby S. S. Melcher M. Nagler M. (2016). Proteomics and comparative genomics of Nitrososphaera viennensis reveal the core genome and adaptations of archaeal ammonia oxidizers. *Proc. Natl. Acad. Sci. U.S.A.* 113:E7937–E7946. 10.1073/pnas.1601212113 27864514 PMC5150414

[B158] Khadka R. B. Uphoff N. (2019). Effects of trichoderma seedling treatment with system of rice intensification management and with conventional management of transplanted rice. *PeerJ* 2019:e5877. 10.7717/PEERJ.5877/SUPP-2 PMC6343584 30693151

[B159] Khairul Alam M. Bell R. W. Hasanuzzaman M. Salahin N. Rashid M. H. Akter N. (2020). Rice (Oryza sativa L.) Establishment Techniques and Their Implications for Soil Properties, Global Warming Potential Mitigation and Crop Yields. *Agronomy* 10:888. 10.3390/AGRONOMY10060888

[B160] Kim H. Lee Y. H. (2020). The rice microbiome: A model platform for crop holobiome. *Phytobiomes J.* 4 5–18.

[B161] Kimura M. Asakawa S. (2005). Comparison of community structures of microbiota at main habitats in rice field ecosystems based on phospholipid fatty acid analysis. *Biol. Fertil. Soils* 43 20–29. 10.1007/S00374-005-0057-2

[B162] Klinnawee L. Noirungsee N. Nopphakat K. Runsaeng P. Chantarachot T. (2021). Flooding overshadows phosphorus availability in controlling the intensity of arbuscular mycorrhizal colonization in Sangyod Muang Phatthalung lowland indica rice. *ScienceAsia* 47 202–210. 10.2306/SCIENCEASIA1513-1874.2021.025

[B163] Koch H. Lücker S. Albertsen M. Kitzinger K. Herbold C. Spieck E. (2015). Expanded metabolic versatility of ubiquitous nitrite-oxidizing bacteria from the genus Nitrospira. *Proc. Natl. Acad. Sci. U.S.A.* 112 11371–11376. 10.1073/PNAS.1506533112 26305944 PMC4568715

[B164] Koch M. Naumann M. Pawelzik E. Gransee A. Thiel H. (2019). The Importance of Nutrient Management for Potato Production Part I: Plant Nutrition and Yield. *Potato Res.* 63 97–119. 10.1007/S11540-019-09431-2

[B165] Kojima S. Bohner A. Gassert B. Yuan L. Wirén N.. (2007). AtDUR3 represents the major transporter for high-affinity urea transport across the plasma membrane of nitrogen-deficient Arabidopsis roots. *Plant J.* 52 30–40. 10.1111/J.1365-313X.2007.03223.X 17672841

[B166] Kumar N. Chhokar R. S. Meena R. P. Kharub A. S. Gill S. C. Tripathi S. C. (2021). Challenges and opportunities in productivity and sustainability of rice cultivation system: a critical review in Indian perspective. *Cereal Res. Commun.* [Epub ahead of print]. 10.1007/S42976-021-00214-5 34642509 PMC8498983

[B167] Ladha J. K. Peoples M. B. Reddy P. M. Biswas J. C. Bennett A. Jat M. L. (2022). Biological nitrogen fixation and prospects for ecological intensification in cereal-based cropping systems. *F. Crop. Res.* 283:108541. 10.1016/J.FCR.2022.108541 35782167 PMC9133800

[B168] LaHue G. T. Chaney R. L. Adviento-Borbe M. A. Linquist B. A. (2016). Alternate wetting and drying in high yielding direct-seeded rice systems accomplishes multiple environmental and agronomic objectives. *Agric. Ecosyst. Environ.* 229 30–39. 10.1016/j.agee.2016.05.020

[B169] Lama S. Velescu A. Leimer S. Weigelt A. Chen H. Eisenhauer N. (2020). Plant diversity influenced gross nitrogen mineralization, microbial ammonium consumption and gross inorganic N immobilization in a grassland experiment. *Oecologia* 193 731–748. 10.1007/S00442-020-04717-6/FIGURES/5 32737568 PMC7406533

[B170] Lammerts van Bueren E. T. Struik P. C. (2017). Diverse concepts of breeding for nitrogen use efficiency. A review. *Agron. Sustain. Dev.* 37:50. 10.1007/S13593-017-0457-3

[B171] Lassaletta L. Billen G. Garnier J. Bouwman L. Velazquez E. Mueller N. D. (2016). Nitrogen use in the global food system: past trends and future trajectories of agronomic performance, pollution, trade, and dietary demand. *Environ. Res. Lett.* 11:095007. 10.1088/1748-9326/11/9/095007

[B172] Lawrencia D. Wong S. K. Low D. Y. S. Goh B. H. Goh J. K. Ruktanonchai U. R. (2021). Controlled Release Fertilizers: A Review on Coating Materials and Mechanism of Release. *Plants* 10:238. 10.3390/PLANTS10020238 33530608 PMC7912041

[B173] Lee S. (2021). Recent advances on nitrogen use efficiency in rice. *Agronomy* 11:753. 10.3390/agronomy11040753

[B174] Lehtovirta-Morley L. E. (2018). Ammonia oxidation: Ecology, physiology, biochemistry and why they must all come together. *FEMS Microbiol. Lett.* 365:fny058. 10.1093/FEMSLE/FNY058 29668934

[B175] Lei T. Sun X. H. Guo X. H. Ma J. J. (2017). Quantifying the relative importance of soil moisture, nitrogen, and temperature on the urea hydrolysis rate. *Soil Sci. Plant Nutr.* 63 225–232. 10.1080/00380768.2017.1340813

[B176] Li H. Xia Y. Zhang G. Zheng G. Fan M. Zhao H. (2022). Effects of straw and straw-derived biochar on bacterial diversity in soda saline-alkaline paddy soil. *Ann. Microbiol.* 72:15. 10.1186/S13213-022-01673-9/TABLES/3 28736578

[B177] Li M. Wei G. Shi W. Sun Z. Li H. Wang X. (2018). Distinct distribution patterns of ammonia-oxidizing archaea and bacteria in sediment and water column of the Yellow River estuary. *Sci. Rep.* 8:1584. 10.1038/S41598-018-20044-6 29371667 PMC5785527

[B178] Li Q. Cui X. Liu X. Roelcke M. Pasda G. Zerulla W. (2017). A new urease-inhibiting formulation decreases ammonia volatilization and improves maize nitrogen utilization in North China Plain. *Sci. Rep.* 71:43853. 10.1038/srep43853 28272451 PMC5341050

[B179] Li Y. Huang L. Zhang H. Wang M. Liang Z. (2017). Assessment of ammonia volatilization losses and nitrogen utilization during the rice growing season in alkaline salt-affected soils. *Sustain* 9:132. 10.3390/su9010132

[B180] Li Q. Yang A. Wang Z. Roelcke M. Chen X. Zhang F. (2015). Effect of a new urease inhibitor on ammonia volatilization and nitrogen utilization in wheat in north and northwest China. *F. Crop. Res.* 175 96–105. 10.1016/J.FCR.2015.02.005

[B181] Li S. Wang X. Zhang X. Liu Z. Zhao H. Zhao Z. (2019). Effects of swine slurry application on ammonia emission, nitrogen utilization and apparent balance of a winter wheat-summer maize rotation system. *Chin. J. Eco Agricult.* 27 1502–1514. 10.13930/J.CNKI.CJEA.190150

[B182] Li Z. Tang H. Yang P. Wu W. Chen Z. Zhou Q. (2012). Spatio-temporal responses of cropland phenophases to climate change in Northeast China. *J. Geogr. Sci.* 221 29–45. 10.1007/S11442-012-0909-2

[B183] Liang D. Ouyang Y. Tiemann L. Robertson G. P. (2020). Niche Differentiation of Bacterial Versus Archaeal Soil Nitrifiers Induced by Ammonium Inhibition Along a Management Gradient. *Front. Microbiol.* 11:2753. 10.3389/FMICB.2020.568588/BIBTEX PMC7689314 33281763

[B184] Liu J. Ma K. Ciais P. Polasky S. (2016). Reducing human nitrogen use for food production. *Sci. Rep.* 6:30104. 10.1038/SREP30104 27445108 PMC4957089

[B185] Liu J. Yu Z. Yao Q. Sui Y. Shi Y. Chu H. (2018). Ammonia-Oxidizing Archaea Show More Distinct Biogeographic Distribution Patterns than Ammonia-Oxidizing Bacteria across the Black Soil Zone of Northeast China. *Front. Microbiol.* 9:171. 10.3389/fmicb.2018.00171 29497404 PMC5819564

[B186] Liu T. Huang J. Chai K. Cao C. Li C. (2018). Effects of N fertilizer sources and tillage practices on NH3 volatilization, grain yield, and n use efficiency of rice fields in central China. *Front. Plant Sci.* 9:385. 10.3389/FPLS.2018.00385/BIBTEX 29623086 PMC5874310

[B187] Liu L. Ding M. Zhou L. Chen Y. Li H. Zhang F. (2021). Effects of different rice straw on soil microbial community structure. *Agron. J.* 113 794–805. 10.1002/AGJ2.20509

[B188] Liu L. Wang E. Zhu Y. Tang L. (2012). Contrasting effects of warming and autonomous breeding on single-rice productivity in China. *Agric. Ecosyst. Environ.* 149 20–29. 10.1016/J.AGEE.2011.12.008

[B189] Liu L. Zhang X. Xu W. Liu X. Li Y. Wei J. (2020). Ammonia volatilization as the major nitrogen loss pathway in dryland agro-ecosystems. *Environ. Pollut.* 265:114862. 10.1016/j.envpol.2020.114862 32497822

[B190] Liu L. Zhu Y. Tang L. Cao W. Wang E. (2013). Impacts of climate changes, soil nutrients, variety types and management practices on rice yield in East China: A case study in the Taihu region. *F. Crop. Res.* 149 40–48. 10.1016/J.FCR.2013.04.022

[B191] Liu Y. Liu Y. Ding Y. Zheng J. Zhou T. Pan G. (2014). Abundance, Composition and Activity of Ammonia Oxidizer and Denitrifier Communities in Metal Polluted Rice Paddies from South China. *PLoS One* 9:e102000. 10.1371/JOURNAL.PONE.0102000 25058658 PMC4109924

[B192] Liu Y. Wang E. Yang X. Wang J. (2010). Contributions of climatic and crop varietal changes to crop production in the North China Plain, since 1980s. *Glob. Chang. Biol.* 16 2287–2299. 10.1111/J.1365-2486.2009.02077.X

[B193] Lobell D. B. Field C. B. (2007). Global scale climate–crop yield relationships and the impacts of recent warming. *Environ. Res. Lett.* 2:014002. 10.1088/1748-9326/2/1/014002

[B194] Lobell D. B. Schlenker W. Costa-Roberts J. (2011). Climate trends and global crop production since 1980. *Science* 333 616–620. 10.1126/SCIENCE.1204531 21551030

[B195] Lopes A. R. Manaia C. M. Nunes O. C. (2014). Bacterial community variations in an alfalfa-rice rotation system revealed by 16S rRNA gene 454-pyrosequencing. *FEMS Microbiol. Ecol.* 87 650–663. 10.1111/1574-6941.12253 24245591

[B196] Lorenz C. Kunstmann H. Lorenz C. Kunstmann H. (2012). The Hydrological Cycle in Three State-of-the-Art Reanalyses: Intercomparison and Performance Analysis. *J. Hydrometeorol.* 13 1397–1420. 10.1175/JHM-D-11-088.1 35865671

[B197] Lu L. Li H. He Y. Zhang J. Xiao J. Peng C. (2018). Compositional Shifts in Ammonia-Oxidizing Microorganism Communities of Eight Geographically Different Paddy Soils —Biogeographical Distribution of Ammonia-Oxidizing Microorganisms. *Agric. Sci.* 9 351–373. 10.4236/AS.2018.93025

[B198] Lu X. Taylor A. E. Myrold D. D. Neufeld J. D. (2020). Expanding perspectives of soil nitrification to include ammonia-oxidizing archaea and comammox bacteria. *Soil Sci. Soc. Am. J.* 84 287–302. 10.1002/SAJ2.20029

[B199] Lukumbuzya M. Kristensen J. M. Kitzinger K. Pommerening-Röser A. Nielsen P. H. Wagner M. (2020). A refined set of rRNA-targeted oligonucleotide probes for in situ detection and quantification of ammonia-oxidizing bacteria. *bioRxiv* [Preprint]. 10.1101/2020.05.27.119446 32916620

[B200] Luo S. Jiang Z. Chou J. Tu G. Wang S. (2022). Response of Temperature-Related Rice Disaster to Different Warming Levels Under an RCP8.5 Emission Scenario in a Major Rice Production Region of China. *Front. Clim.* 3:195. 10.3389/FCLIM.2021.736459/BIBTEX

[B201] Luxhøi J. Recous S. Fillery I. R. P. Murphy D. V. Jensen L. S. (2005). Comparison of 15NH 4+ pool dilution techniques to measure gross N fluxes in a coarse textured soil. *Soil Biol. Biochem.* 37 569–572. 10.1016/j.soilbio.2004.09.004

[B202] Ma X. Zhang F. Liu F. Guo G. Cheng T. Wang J. (2022). An Integrated Nitrogen Management Strategy Promotes Open-Field Pepper Yield, Crop Nitrogen Uptake, and Nitrogen Use Efficiency in Southwest China. *Agriculture* 12:524. 10.3390/AGRICULTURE12040524

[B203] Ma M. Song C. Fang H. Zhang J. Wei J. Liu S. (2022). Development of a Process-Based N2O Emission Model for Natural Forest and Grassland Ecosystems. *J. Adv. Model. Earth Syst.* 14:e2021MS002460. 10.1029/2021MS002460

[B204] Ma Y. Schwenke G. Sun L. Liu D. L. Wang B. Yang B. (2018). Modeling the impact of crop rotation with legume on nitrous oxide emissions from rain-fed agricultural systems in Australia under alternative future climate scenarios. *Sci. Total Environ.* 630 1544–1552. 10.1016/j.scitotenv.2018.02.322 29554771

[B205] Maeda K. Hanajima D. Toyoda S. Yoshida N. Morioka R. Osada T. (2011). Microbiology of nitrogen cycle in animal manure compost. *Microb. Biotechnol.* 4 700–709. 10.1111/j.1751-7915.2010.00236.x 21375720 PMC3815407

[B206] Mahabubur Rahman M. Yamamoto A. (2021). “Methane Cycling in Paddy Field: A global warming issue,” in *Agrometeorology*, ed. Meena R. S. (London: IntechOpen). 10.5772/INTECHOPEN.94200

[B207] Mahal N. K. Osterholz W. R. Miguez F. E. Poffenbarger H. J. Sawyer J. E. Olk D. C. (2019). Nitrogen fertilizer suppresses mineralization of soil organic matter in maize agroecosystems. *Front. Ecol. Evol.* 7:59. 10.3389/FEVO.2019.00059/BIBTEX

[B208] Maharjan B. Venterea R. T. (2013). Nitrite intensity explains N management effects on N2O emissions in maize. *Soil Biol. Biochem.* 66 229–238. 10.1016/J.SOILBIO.2013.07.015

[B209] Mahdu O. Mills B. (2019). *The Impacts of Climate Change on Rice Production and Small Farmers ‘ Adaptation : A Case of Guyana The Impacts of Climate Change on Rice Production and Small Farmers ’ Adaptation : A Case of Guyana.* Blacksburg, VA:Virginia Tech

[B210] Mahmud K. Panday D. Mergoum A. Missaoui A. (2021). Nitrogen losses and potential mitigation strategies for a sustainable agroecosystem. *Sustainability* 13 1–23. 10.3390/su13042400

[B211] Manik S. M. N. Pengilley G. Dean G. Field B. Shabala S. Zhou M. (2019). Soil and crop management practices to minimize the impact of waterlogging on crop productivity. *Front. Plant Sci.* 10:140. 10.3389/FPLS.2019.00140/BIBTEX 30809241 PMC6379354

[B212] Manjunath M. Kumar U. Yadava R. B. Rai A. B. Singh B. (2018). Influence of organic and inorganic sources of nutrients on the functional diversity of microbial communities in the vegetable cropping system of the Indo-Gangetic plains. *C. R. Biol.* 341 349–357. 10.1016/J.CRVI.2018.05.002 29861196

[B213] Mannke F. (2011). Key Themes of Local Adaptation to Climate Change: Results from Mapping Community-Based Initiatives in Africa. *Clim. Chang. Manag.* 17–32. 10.1007/978-3-642-22315-0_2

[B214] Manschadi A. M. Soltani A. (2021). Variation in traits contributing to improved use of nitrogen in wheat: Implications for genotype by environment interaction. *F. Crop. Res.* 270:108211. 10.1016/J.FCR.2021.108211

[B215] Maragatham N. Gj M. Poongodi T. (2010). “Effect of Nitrogen sources on Aerobic Rice production under various rice soil Eco systems,” in *Proceedings of 19th World Congress of Soil Science, Soil Solutions for a Changing World*, Brisbane, QLD, 13–16.

[B216] Martikainen P. J. (2022). Heterotrophic nitrification – An eternal mystery in the nitrogen cycle. *Soil Biol. Biochem.* 168:108611. 10.1016/J.SOILBIO.2022.108611

[B217] Masclaux-Daubresse C. Daniel-Vedele F. Dechorgnat J. Chardon F. Gaufichon L. Suzuki A. (2010). Nitrogen uptake, assimilation and remobilization in plants: challenges for sustainable and productive agriculture. *Ann. Bot.* 105 1141–1157. 10.1093/AOB/MCQ028 20299346 PMC2887065

[B218] Matsuo N. Mochizuki T. (2009). Growth and yield of six rice cultivars under three water-saving cultivations. *Plant Prod. Sci.* 12 514–525. 10.1626/pps.12.514

[B219] Mauceri A. Bassolino L. Lupini A. Badeck F. Rizza F. Schiavi M. (2020). Genetic variation in eggplant for Nitrogen Use Efficiency under contrasting NO3- supply. *J. Integr. Plant Biol.* 62 487–508. 10.1111/JIPB.12823/SUPPINFO 31087763

[B220] Mboyerwa P. A. Kibret K. Mtakwa P. W. Aschalew A. (2021). Evaluation of growth, yield, and water productivity of paddy rice with water-saving irrigation and optimization of nitrogen fertilization. *Agronomy* 11:1629. 10.3390/agronomy11081629

[B221] Mboyerwa P. A. Kibret K. Mtakwa P. Aschalew A. (2022). Lowering nitrogen rates under the system of rice intensification enhanced rice productivity and nitrogen use efficiency in irrigated lowland rice. *Heliyon* 8:e09140. 10.1016/j.heliyon.2022.e09140 35846470 PMC9280497

[B222] Meng X. Li Y. Yao H. Wang J. Dai F. Wu Y. (2020). Nitrification and urease inhibitors improve rice nitrogen uptake and prevent denitrification in alkaline paddy soil. *Appl. Soil Ecol.* 154:103665. 10.1016/j.apsoil.2020.103665

[B223] Messiga A. J. Nyamaizi S. Yu S. Dorais M. (2021). Blueberry yield and soil mineral nitrogen response to nitrogen fertilizer and nitrification inhibitors under drip-fertigation systems. *Agronomy* 11:2144. 10.3390/agronomy11112144

[B224] Min W. Guo H. J. Zhang W. Zhou G. W. Ma L. J. Ye J. (2016). Irrigation water salinity and N fertilization: Effects on ammonia oxidizer abundance, enzyme activity and cotton growth in a drip irrigated cotton field. *J. Integr. Agric.* 15 1121–1131. 10.1016/S2095-3119(15)61158-3

[B225] Moloantoa K. M. Khetsha Z. P. van Heerden E. Castillo J. C. Cason E. D. (2022). Nitrate Water Contamination from Industrial Activities and Complete Denitrification as a Remediation Option. *Water* 14:799. 10.3390/W14050799

[B226] Mongiano G. Titone P. Pagnoncelli S. Sacco D. Tamborini L. Pilu R. (2020). Phenotypic variability in Italian rice germplasm. *Eur. J. Agron.* 120:126131. 10.1016/j.eja.2020.126131

[B227] Moore K. J. Anex R. P. Elobeid A. E. Fei S. Flora C. B. Goggi A. S. (2019). Regenerating Agricultural Landscapes with Perennial Groundcover for Intensive Crop Production. *Agronomy* 9:458. 10.3390/AGRONOMY9080458

[B228] Móring A. Hooda S. Raghuram N. Adhya T. K. Ahmad A. Bandyopadhyay S. K. (2021). Nitrogen Challenges and Opportunities for Agricultural and Environmental Science in India. *Front. Sustain. Food Syst.* 5:13. 10.3389/FSUFS.2021.505347/BIBTEX

[B229] Mukhtar H. Lin Y. P. Anthony J. (2017). Ammonia Oxidizing Archaea and Bacteria in East Asian Paddy Soils—A Mini Review. *Environ.* 4:84. 10.3390/ENVIRONMENTS4040084

[B230] Mukhtar H. Lin Y. P. Lin C. M. Lin Y. R. (2019). Relative abundance of ammonia oxidizing archaea and bacteria influences soil nitrification responses to temperature. *Microorganisms* 7:526. 10.3390/microorganisms7110526 31690001 PMC6920900

[B231] Müller C. Stevens R. J. Laughlin R. J. (2004). A 15N tracing model to analyse N transformations in old grassland soil. *Soil Biol. Biochem.* 36 619–632. 10.1016/J.SOILBIO.2003.12.006

[B232] Nacke H. Schöning I. Schindler M. Schrumpf M. Daniel R. Nicol G. W. (2017). Links between seawater flooding, soil ammonia oxidiser communities and their response to changes in salinity. *FEMS Microbiol. Ecol* 93:fix144. 10.1093/FEMSEC/FIX144 29069386

[B233] Naser H. M. Nagata O. Sultana S. Hatano R. (2020). Carbon sequestration and contribution of CO2, CH4 and N2O fluxes to global warming potential from paddy-fallow fields on mineral soil beneath peat in Central Hokkaido, Japan. *Agriculture* 10:6. 10.3390/agriculture10010006

[B234] Naz M. Y. Sulaiman S. A. (2017). Attributes of natural and synthetic materials pertaining to slow-release urea coating industry. *Rev. Chem. Eng.* 33 293–308. 10.1515/REVCE-2015-0065/MACHINEREADABLECITATION/RIS

[B235] Nehe A. S. Misra S. Murchie E. H. Chinnathambi K. Foulkes M. J. (2018). Genetic variation in N-use efficiency and associated traits in Indian wheat cultivars. *F. Crop. Res.* 225:152. 10.1016/J.FCR.2018.06.002 30078934 PMC6065306

[B236] Nie L. Peng S. Chen M. Shah F. Huang J. Cui K. (2012). Aerobic rice for water-saving agriculture. A review. *Agron. Sustain. Dev.* 32 411–418. 10.1007/s13593-011-0055-8

[B237] Nikolajsen M. T. Pacholski A. S. Sommer S. G. (2020). Urea Ammonium Nitrate Solution Treated with Inhibitor Technology: Effects on Ammonia Emission Reduction. Wheat Yield, and Inorganic N in Soil. *Agronomy* 10:161. 10.3390/AGRONOMY10020161

[B238] Nishizawa T. Uei Y. Tago K. Isobe K. Otsuka S. Senoo K. (2013). Taxonomic composition of denitrifying bacterial isolates is different among three rice paddy field soils in Japan. *Soil Sci. Plant Nutr.* 59 305–310. 10.1080/00380768.2013.773256

[B239] Norton J. Ouyang Y. (2019). Controls and Adaptive Management of Nitrification in Agricultural Soils. *Front. Microbiol.* 10:1931. 10.3389/fmicb.2019.01931 31543867 PMC6728921

[B240] Oelbermann M. (ed.) (2014). *Sustainable Agroecosystems In Climate Change Mitigation.* Wageningen: Wageningen Academic Publishers, 10.3920/978-90-8686-788-2

[B241] Oladele S. Adeyemo A. Awodun M. Ajayi A. Fasina A. (2019). Effects of biochar and nitrogen fertilizer on soil physicochemical properties, nitrogen use efficiency and upland rice (Oryza sativa) yield grown on an Alfisol in Southwestern Nigeria. *Int. J. Recycl. Org. Waste Agric.* 8 295–308. 10.1007/S40093-019-0251-0/TABLES/6

[B242] Omomowo O. I. Babalola O. O. (2019). Bacterial and Fungal Endophytes: Tiny Giants with Immense Beneficial Potential for Plant Growth and Sustainable Agricultural Productivity. *Microorganisms* 7:481. 10.3390/MICROORGANISMS7110481 31652843 PMC6921065

[B243] Onoja U. S. Dibua U. M. E. Enete A. A. (2011). Climate Change: Causes, Effects and Mitigation Measures-a Review. *Glob. J. Pure Appl. Sci.* 17 469–479.

[B244] Oo A. Z. Sudo S. Inubushi K. Mano M. Yamamoto A. Ono K. (2018). Methane and nitrous oxide emissions from conventional and modified rice cultivation systems in South India. *Agric. Ecosyst. Environ.* 252 148–158. 10.1016/J.AGEE.2017.10.014

[B245] Oshiki M. Segawa T. Ishii S. (2018). Nitrogen Cycle Evaluation (NiCE) Chip for Simultaneous Analysis of Multiple N Cycle-Associated Genes. *Appl. Environ. Microbiol* 84:e02615–17. 10.1128/AEM.02615-17 29427421 PMC5881049

[B246] Otte J. M. Blackwell N. Ruser R. Kappler A. Kleindienst S. Schmidt C. (2019). N2O formation by nitrite-induced (chemo)denitrification in coastal marine sediment. *Sci. Rep.* 9:10691. 10.1038/s41598-019-47172-x 31366952 PMC6668465

[B247] Pan J. Liu Y. Zhong X. Lampayan R. M. Singleton G. R. Huang N. (2017). Grain yield, water productivity and nitrogen use efficiency of rice under different water management and fertilizer-N inputs in South China. *Agric. Water Manag.* 184 191–200. 10.1016/J.AGWAT.2017.01.013

[B248] Pan S.-Y. He K.-H. Lin K.-T. Fan C. Chang C.-T. (2022). Addressing nitrogenous gases from croplands toward low-emission agriculture. *Npj Clim. Atmos. Sci.* 5:43. 10.1038/s41612-022-00265-3

[B249] Paterson R. R. M. Lima N. (2010). How will climate change affect mycotoxins in food? *Food Res. Int.* 43 1902–1914. 10.1016/J.FOODRES.2009.07.010

[B250] Pathak H. Li C. Wassmann R. (2005). Greenhouse gas emissions from Indian rice fields: calibration and upscaling using the DNDC model. *Biogeosci. Discuss.* 2 77–102. 10.5194/bgd-2-77-2005

[B251] Peng S. Buresh R. J. Huang J. Zhong X. Zou Y. Yang J. (2010). Improving nitrogen fertilization in rice by sitespecific N management. A review. *Agron. Sustain. Dev.* 303 649–656. 10.1051/AGRO/2010002

[B252] Peng S. Tang Q. Zou Y. (2009). Current status and challenges of rice production in China. *Plant Prod. Sci.* 12 3–8. 10.1626/pps.12.3

[B253] Peng X. Yando E. Hildebrand E. Dwyer C. Kearney A. Waciega A. (2012). Differential responses of ammonia-oxidizing archaea and bacteria to long-term fertilization in a New England salt marsh. *Front. Microbiol.* 3:445. 10.3389/FMICB.2012.00445 23346081 PMC3551258

[B254] Perchlik M. Tegeder M. (2017). Improving Plant Nitrogen Use Efficiency through Alteration of Amino Acid Transport Processes. *Plant Physiol.* 175 235–247. 10.1104/PP.17.00608 28733388 PMC5580756

[B255] Prasanna R. Nain L. Pandey A. K. Saxena A. K. (2011). Microbial diversity and multidimensional interactions in the rice ecosystem. *Arch. Agron. Soil Sci.* 58 723–744. 10.1080/03650340.2010.537325

[B256] Prosser J. I. Nicol G. W. (2012). Archaeal and bacterial ammonia-oxidisers in soil: the quest for niche specialisation and differentiation. *Trends Microbiol.* 20 523–531. 10.1016/j.tim.2012.08.001 22959489

[B257] Qaswar M. Jing H. Ahmed W. Shujun L. Dongchu L. Lu Z. (2019). Substitution of Inorganic Nitrogen Fertilizer with Green Manure (GM) Increased Yield Stability by Improving C Input and Nitrogen Recovery Efficiency in Rice Based Cropping System. *Agronomy* 9:609. 10.3390/AGRONOMY9100609

[B258] Qiu S. J. Ju X. T. Ingwersen J. Guo Z. De, Stange C. F. (2013). Role of Carbon Substrates Added in the Transformation of Surplus Nitrate to Organic Nitrogen in a Calcareous Soil. *Pedosphere* 23 205–212. 10.1016/S1002-0160(13)60008-9

[B259] Qureshi A. S. (2018). “Challenges and Opportunities of Groundwater Management in Pakistan,”. *Groundwater of South Asia* (ed) Mukherjee A. (Berlin: Springer) 10.1007/978-981-10-3889-1_43

[B260] Rachwał K. Gustaw K. Kazimierczak W. Waśko A. (2021). Is soil management system really important? comparison of microbial community diversity and structure in soils managed under organic and conventional regimes with some view on soil properties. *PLoS One* 16:e0256969. 10.1371/JOURNAL.PONE.0256969 34499697 PMC8428661

[B261] Rahman M. M. Islam M. A. Azirun M. S. Boyce A. N. (2014). Agronomic and nitrogen recovery efficiency of rice under tropical conditions as affected by nitrogen fertilizer and legume crop rotation. *J. Anim. Plant Sci.* 24 891–896.

[B262] Rajta A. Bhatia R. Setia H. Pathania P. (2020). Role of heterotrophic aerobic denitrifying bacteria in nitrate removal from wastewater. *J. Appl. Microbiol.* 128 1261–1278. 10.1111/JAM.14476 31587489

[B263] Ransom C. J. Jolley V. D. Blair T. A. Sutton L. E. Hopkins B. G. (2020). Nitrogen release rates from slow- and controlled-release fertilizers influenced by placement and temperature. *PLoS One* 15:e0234544. 10.1371/JOURNAL.PONE.0234544 32555670 PMC7299380

[B264] Rasul G. (2016). Managing the food, water, and energy nexus for achieving the Sustainable Development Goals in South Asia. *Environ. Dev.* 18 14–25. 10.1016/J.ENVDEV.2015.12.001

[B265] Rawal N. Pande K. R. Shrestha R. Vista S. P. (2022). Nutrient use efficiency (NUE) of wheat (Triticum aestivum L.) as affected by NPK fertilization. *PLoS One* 17:e0262771. 10.1371/JOURNAL.PONE.0262771 35085333 PMC8794114

[B266] Raza A. Razzaq A. Mehmood S. S. Zou X. Zhang X. Lv Y. (2019). Impact of Climate Change on Crops Adaptation and Strategies to Tackle Its Outcome: A Review. *Plants* 8:34. 10.3390/PLANTS8020034 30704089 PMC6409995

[B267] Raza S. Zhou J. Aziz T. Afzal M. R. Ahmed M. Javaid S. (2018). Piling up reactive nitrogen and declining nitrogen use efficiency in Pakistan: A challenge not challenged (1961-2013). *Environ. Res. Lett.* 13:034012. 10.1088/1748-9326/aaa9c5

[B268] Ren X. Zhang J. Bah H. Müller C. Cai Z. Zhu B. (2021). Soil gross nitrogen transformations in forestland and cropland of Regosols. *Sci. Rep.* 11:223. 10.1038/s41598-020-80395-x 33420303 PMC7794575

[B269] Riache M. Revilla P. Malvar R. A. Djemel A. Chemlal A. Mefti M. (2022). Assessment of Nitrogen Use Efficiency in Algerian Saharan Maize Populations for Tolerance under Drought and No-Nitrogen Stresses. *Agronomy* 12:1123. 10.3390/agronomy12051123

[B270] Rochette P. Angers D. A. Chantigny M. H. Gasser M. O. MacDonald J. D. Pelster D. E. (2013). NH3 volatilization, soil NH4+concentration and soil pH following subsurface banding of urea at increasing rates. *Can. J. Soil Sci.* 93 261–268. 10.4141/CJSS2012-095/ASSET/IMAGES/LARGE/CJSS2012-095F4.JPEG

[B271] Rodriguez D. G. P. (2020). An assessment of the site-specific nutrient management (SSNM) strategy for irrigated rice in Asia. *Agriculture* 10:559. 10.3390/agriculture10110559

[B272] Roozbeh M. Rajaie M. (2021). Effects of residue management and nitrogen fertilizer rates on accumulation of soil residual nitrate and wheat yield under no-tillage system in south-west of Iran. *Int. Soil Water Conserv. Res.* 9 116–126. 10.1016/J.ISWCR.2020.09.007

[B273] Rose M. T. Patti A. F. Little K. R. Brown A. L. Jackson W. R. Cavagnaro T. R. (2014). A Meta-Analysis and Review of Plant-Growth Response to Humic Substances: Practical Implications for Agriculture. *Adv. Agron.* 124 37–89. 10.1016/B978-0-12-800138-7.00002-4

[B274] Rupp H. Tauchnitz N. Meissner R. (2021). The effects of soil drying out and rewetting on nitrogen and carbon leaching–results of a long-term lysimeter experiment. *Water* 13:2601. 10.3390/w13182601

[B275] Ruser R. Schulz R. (2015). The effect of nitrification inhibitors on the nitrous oxide (N2O) release from agricultural soils—a review. *J. Plant Nutr. Soil Sci.* 178 171–188. 10.1002/JPLN.201400251

[B276] Sa̧dej W. Przekwas K. (2008). Fluctuations of nitrogen levels in soil profile under conditions of a long-term fertilization experiment. *Plant Soil Environ.* 54 197–203. 10.17221/394-pse

[B277] Sahrawat K. L. (2010). Nitrogen mineralization in lowland rice soils: The role of organic matter quantity and quality. *Arch. Agron. Soil Sci.* 56 337–353. 10.1080/03650340903093158

[B278] Sahu K. P. Kumar A. Sakthivel K. Reddy B. Kumar M. Patel A. (2022). Deciphering core phyllomicrobiome assemblage on rice genotypes grown in contrasting agroclimatic zones: implications for phyllomicrobiome engineering against blast disease. *Environ. Microbiome* 17:28. 10.1186/S40793-022-00421-5 35619157 PMC9134649

[B279] Saiful Alam M. Ren G. Lu L. Zheng Y. Peng X. Jia Z. (2013). Ecosystem-specific selection of microbial ammonia oxidizers in an acid soil. *Biogeosci. Discuss.* 10 1717–1746. 10.5194/bgd-10-1717-2013

[B280] Saito T. Ishii S. Otsuka S. Nishiyama M. Senoo K. (2008). Identification of Novel Betaproteobacteria in a Succinate-Assimilating Population in Denitrifying Rice Paddy Soil by Using Stable Isotope Probing. *Microbes Environ.* 23 192–200. 10.1264/jsme2.23.192 21558708

[B281] Sandhu N. Sethi M. Kumar A. Dang D. Singh J. Chhuneja P. (2021). Biochemical and Genetic Approaches Improving Nitrogen Use Efficiency in Cereal Crops: A Review. *Front. Plant Sci.* 12:757. 10.3389/FPLS.2021.657629/BIBTEX PMC8213353 34149755

[B282] Sandhu N. Yadaw R. B. Chaudhary B. Prasai H. Iftekharuddaula K. Venkateshwarlu C. (2019). Evaluating the performance of rice genotypes for improving yield and adaptability under direct seeded aerobic cultivation conditions. *Front. Plant Sci.* 10:159. 10.3389/FPLS.2019.00159/FULL 30828343 PMC6384261

[B283] Sarkar S. Skalicky M. Hossain A. Brestic M. Saha S. Garai S. (2020). Management of Crop Residues for Improving Input Use Efficiency and Agricultural Sustainability. *Sustainability* 12:9808. 10.3390/SU12239808

[B284] Sarwar N. Atique-ur-Rehman, Farooq O. Wasaya A. Hussain M. El-Shehawi A. M. (2021). Integrated nitrogen management improves productivity and economic returns of wheat-maize cropping system. *J. King Saud Univ. Sci.* 33:101475. 10.1016/J.JKSUS.2021.101475

[B285] Saud S. Wang D. Fahad S. Alharby H. F. Bamagoos A. A. Mjrashi A. (2022). Comprehensive Impacts of Climate Change on Rice Production and Adaptive Strategies in China. *Front. Microbiol.* 13:2254. 10.3389/FMICB.2022.926059 35875578 PMC9300054

[B286] Sawada K. Funakawa S. Toyota K. Kosaki T. (2015). Potential nitrogen immobilization as influenced by available carbon in Japanese arable and forest soils. *Soil Sci. Plant Nutr.* 61 917–926. 10.1080/00380768.2015.1075364

[B287] Schmidt J. E. Mazza Rodrigues J. L. Brisson V. L. Kent A. Gaudin A. C. M. (2020). Impacts of directed evolution and soil management legacy on the maize rhizobiome. *Soil Biol. Biochem.* 145 1–37. 10.1016/j.soilbio.2020.107794

[B288] Sebilo M. Mayer B. Nicolardot B. Pinay G. Mariotti A. (2013). Long-term fate of nitrate fertilizer in agricultural soils. *Proc. Natl. Acad. Sci. U.S.A.* 110 18185–18189. 10.1073/PNAS.1305372110/SUPPL_FILE/PNAS.201305372SI.PDF 24145428 PMC3831475

[B289] Sekaran U. Lai L. Ussiri D. A. N. Kumar S. Clay S. (2021). Role of integrated crop-livestock systems in improving agriculture production and addressing food security – A review. *J. Agric. Food Res.* 5:100190. 10.1016/J.JAFR.2021.100190

[B290] Seleiman M. F. Elshayb O. M. Nada A. M. El-Leithy S. A. Baz L. Alhammad B. A. (2022). Azolla Compost as an Approach for Enhancing Growth. Productivity and Nutrient Uptake of *Oryza sativa L*. *Agronomy* 12:416. 10.3390/AGRONOMY12020416

[B291] Shah A. L. Naher U. A. Hasan Z. Panhwar Q. A. Radziah O. (2014). Influence of arsenic on rice growth and its mitigation with different water management techniques. *Asian J. Crop Sci.* 6 373–382. 10.3923/AJCS.2014.373.382

[B292] Shahane A. A. Shivay Y. S. Prasanna R. Kumar D. (2019). Nitrogen nutrition and use efficiency in rice as influenced by crop establishment methods, cyanobacterial and phosphate solubilizing bacterial consortia and zinc fertilization. *Commun. Soil Sci. Plant Anal.* 50 1487–1499. 10.1080/00103624.2019.1626876

[B293] Sharma N. Sinha V. B. Prem Kumar N. A. Subrahmanyam D. Neeraja C. N. Kuchi S. (2021). Nitrogen Use Efficiency Phenotype and Associated Genes: Roles of Germination, Flowering, Root/Shoot Length and Biomass. *Front. Plant Sci.* 11:2329. 10.3389/FPLS.2020.587464/BIBTEX PMC7855041 33552094

[B294] Sharma P. Abrol V. Sharma R. K. (2011). Impact of tillage and mulch management on economics, energy requirement and crop performance in maize–wheat rotation in rainfed subhumid inceptisols, India. *Eur. J. Agron.* 34 46–51. 10.1016/J.EJA.2010.10.003

[B295] Sharma S. Panneerselvam P. Castillo R. Manohar S. Raj R. Ravi V. (2019). Web-based tool for calculating field-specific nutrient management for rice in India. *Nutr. Cycl. Agroecosyst.* 113:21. 10.1007/S10705-018-9959-X 32684798 PMC7357723

[B296] Shew A. M. Durand-Morat A. Putman B. Nalley L. L. Ghosh A. (2019). Rice intensification in Bangladesh improves economic and environmental welfare. *Environ. Sci. Policy* 95 46–57. 10.1016/J.ENVSCI.2019.02.004

[B297] Shi Y. Zhang Q. Liu X. Jing X. Shi C. Zheng L. (2022). Organic manure input and straw cover improved the community structure of nitrogen cycle function microorganism driven by water erosion. *Int. Soil Water Conserv. Res.* 10 129–142. 10.1016/J.ISWCR.2021.03.005

[B298] Sigler W. A. Ewing S. A. Jones C. A. Payn R. A. Miller P. Maneta M. (2020). Water and nitrate loss from dryland agricultural soils is controlled by management, soils, and weather. *Agric. Ecosyst. Environ.* 304:107158. 10.1016/J.AGEE.2020.107158

[B299] Signor D. Cerri C. E. P. (2013). Emissões de óxido nitroso em solos agrícolas: Uma revisão. *Pesqui. Agropecu. Trop.* 43 322–338. 10.1590/S1983-40632013000300014

[B300] Silva E. F. Melo M. F. Sombra K. E. S. Silva T. S. de Freitas D. F. da Costa M. E. (2019). “Organic Nitrogen in Agricultural Systems,” in *Nitrogen Fixation*, (eds) Rigobelo E. C. Serra A. P. (London: Intechopen). 10.5772/INTECHOPEN.90242

[B301] Silwal S. Bhattarai S. P. Midmore D. J. (2020). Aerobic rice with or without strategic irrigation in the subtropics. *Agronomy* 10:1831. 10.3390/agronomy10111831

[B302] Singh B. (2018). Are Nitrogen Fertilizers Deleterious to Soil Health? *Agronomy* 8:48 10.3390/agronomy8040048

[B303] Singh B. Mishra S. Bisht S. Joshi R. Singh B. Mishra S. (2021). Growing Rice with Less Water: Improving Productivity by Decreasing Water Demand. *Rice Improv.* 147–170. 10.1007/978-3-030-66530-2_5

[B304] Smith L. E. D. Siciliano G. (2015). A comprehensive review of constraints to improved management of fertilizers in China and mitigation of diffuse water pollution from agriculture. *Agric. Ecosyst. Environ.* 209 15–25. 10.1016/J.AGEE.2015.02.016

[B305] Soares J. R. Cantarella H. Menegale M. L. de C. (2012). Ammonia volatilization losses from surface-applied urea with urease and nitrification inhibitors. *Soil Biol. Biochem.* 52 82–89. 10.1016/J.SOILBIO.2012.04.019

[B306] Solomon S. Qin D. Manning M. Chen Z. Marquis M. Averyt K. B. (2007). *Global Climate Projections.* 1–8. Available online at: https://philpapers.org/rec/SOLGCP (accessed on May 14, 2019).

[B307] Somenahally A. C. Loeppert R. H. Zhou J. Gentry T. J. (2021). Niche Differentiation of Arsenic-Transforming Microbial Groups in the Rice Rhizosphere Compartments as Impacted by Water Management and Soil-Arsenic Concentrations. *Front. Microbiol.* 12:736751. 10.3389/FMICB.2021.736751/FULL 34803950 PMC8602891

[B308] Song Y. N. Lin Z. M. (2014). Abundance and community composition of ammonia-oxidizers in paddy soil at different nitrogen fertilizer rates. *J. Integr. Agric.* 13 870–880. 10.1016/S2095-3119(13)60426-8

[B309] Song Y. Wang C. Linderholm H. W. Fu Y. Cai W. Xu J. (2022). The negative impact of increasing temperatures on rice yields in southern China. *Sci. Total Environ.* 820 153262. 10.1016/J.SCITOTENV.2022.153262 35065105

[B310] Sosulski T. Stępień W. Was A. Szymańska M. (2020). N2 o and co2 emissions from bare soil: Effect of fertilizer management. *Agric.* 10:602. 10.3390/agriculture10120602

[B311] Stanger T. F. Lauer J. G. Chavas J. P. (2008). The profitability and risk of long-term cropping systems featuring different rotations and nitrogen rates. *Agron. J.* 100 105–113. 10.2134/agronj2006.0322

[B312] Stecula D. A. Merkley E. (2019). Framing Climate Change: Economics, Ideology, and Uncertainty in American News Media Content from 1988 to 2014. *Front. Commun.* 4:6. 10.3389/FCOMM.2019.00006/BIBTEX

[B313] Stein L. Y. (2019). Insights into the physiology of ammonia-oxidizing microorganisms. *Curr. Opin. Chem. Biol.* 49 9–15. 10.1016/J.CBPA.2018.09.003 30236860

[B314] Stuecker M. F. Tigchelaar M. Kantar M. B. (2018). Climate variability impacts on rice production in the Philippines. *PLoS One* 13:e0201426. 10.1371/JOURNAL.PONE.0201426 30091991 PMC6084865

[B315] Subbarao G. V. Rao I. M. Nakahara K. Sahrawat K. L. Ando Y. Kawashima T. (2013). Potential for biological nitrification inhibition to reduce nitrification and N2O emissions in pasture crop-livestock systems. *Animal* 7 322–332. 10.1017/s1751731113000761 23739474

[B316] Sun R. Myrold D. D. Wang D. Guo X. Chu H. (2019). AOA and AOB communities respond differently to changes of soil pH under long-term fertilization. *Soil Ecol. Lett.* 1 126–135. 10.1007/s42832-019-0016-8

[B317] Sun S. Yang X. Lin X. Sassenrath G. F. Li K. (2018). Climate-smart management can further improve winter wheat yield in China. *Agric. Syst.* 162 10–18. 10.1016/j.agsy.2018.01.010

[B318] Sun Y. De Vos P. Heylen K. (2016). Nitrous oxide emission by the non-denitrifying, nitrate ammonifier Bacillus licheniformis. *BMC Genomics* 17:68. 10.1186/S12864-016-2382-2/FIGURES/4 26786044 PMC4719734

[B319] Tabari H. (2020). Climate change impact on flood and extreme precipitation increases with water availability. *Sci. Rep.* 10:13768. 10.1038/s41598-020-70816-2 32792563 PMC7426818

[B320] Tamele R. A. Ueno H. Toma Y. Morita N. (2020). Nitrogen recoveries and nitrogen use efficiencies of organic fertilizers with different c/n ratios in maize cultivation with low-fertile soil by15n method. *Agriculture* 10:272. 10.3390/agriculture10070272

[B321] Tang H. Pang J. Zhang G. Takigawa M. Liu G. Zhu J. (2014). Mapping ozone risks for rice in China for years 2000 and 2020 with flux-based and exposure-based doses. *Atmos. Environ.* 86 74–83. 10.1016/J.ATMOSENV.2013.11.078

[B322] Tang H. Xiao X. Li C. Cheng K. Pan X. Li W. (2019). Effects of rhizosphere and long-term fertilisation practices on the activity and community structure of ammonia oxidisers under double-cropping rice field. *Acta Agric. Scand. Sect. B Soil Plant Sci.* 69 356–368. 10.1080/09064710.2019.1576763

[B323] Tao F. Zhang Z. Shi W. Liu Y. Xiao D. Zhang S. (2013). Single rice growth period was prolonged by cultivars shifts, but yield was damaged by climate change during 1981–2009 in China, and late rice was just opposite. *Glob. Chang. Biol.* 19 3200–3209. 10.1111/GCB.12250 23661287

[B324] Taylor A. E. Ottoman C. Chaplen F. (2021). Implications of the Thermodynamic Response of Soil Mineralization, Respiration, and Nitrification on Soil Organic Matter Retention. *Front. Microbiol.* 12:1190. 10.3389/FMICB.2021.651210/BIBTEX PMC8170049 34093466

[B325] Thakur A. K. Mohanty R. K. Singh R. Patil D. U. (2015). Enhancing water and cropping productivity through Integrated System of Rice Intensification (ISRI) with aquaculture and horticulture under rainfed conditions. *Agric. Water Manag.* 161 65–76. 10.1016/J.AGWAT.2015.07.008

[B326] Thakur N. S. Singh M. K. Bhayal L. Meena K. Choudhary S. K. Kumawat N. (2022). Sustainability in Rainfed Maize (Zea mays L.) Production Using Choice of Corn Variety and Nitrogen Scheduling. *Sustainability* 14:3116. 10.3390/su14053116

[B327] Thind S. S. Wu G. Tian M. Chen A. (2012). Significant enhancement in the photocatalytic activity of N, W co-doped TiO2 nanomaterials for promising environmental applications. *Nanotechnology* 23:475706. 23110785 10.1088/0957-4484/23/47/475706

[B328] The S. V. Snyder R. Tegeder M. (2021). Targeting Nitrogen Metabolism and Transport Processes to Improve Plant Nitrogen Use Efficiency. *Front. Plant Sci.* 11:2330. 10.3389/FPLS.2020.628366/BIBTEX PMC7957077 33732269

[B329] Tian D. Chen Z. Lin Y. Liang T. Chen Z. Guo X. (2021). The Interaction between Rice Genotype and Magnaporthe oryzae Regulates the Assembly of Rice Root-Associated Microbiota. *Rice* 14:40. 10.1186/S12284-021-00486-9/FIGURES/6 33974154 PMC8113375

[B330] van Evert F. K. Gaitán-Cremaschi D. Fountas S. Kempenaar C. (2017). Can precision agriculture increase the profitability and sustainability of the production of potatoes and olives? *Sustainability* 9:1863. 10.3390/su9101863

[B331] Viero F. Bayer C. Vieira R. C. B. Carniel E. (2015). Manejo da irrigação e fertilizantes nitrogenados para reduzir a volatilização de amônia. *Rev. Bras. Cienc. Solo* 39 1737–1743. 10.1590/01000683rbcs20150132

[B332] Virk A. L. Farooq M. S. Ahmad A. Khaliq T. Rehmani M. I. A. Haider F. U. (2020). Effect of seedling age on growth and yield of fine rice cultivars under alternate wetting and drying system. *J. Plant Nutr.* 44 1–15. 10.1080/01904167.2020.1812642

[B333] Vogel A. M. Below F. E. (2018). Hybrid selection and agronomic management to lessen the continuous corn yield penalty. *Agronomy* 8:228. 10.3390/agronomy8100228

[B334] Wang X. Wang Y. Zhu F. Zhang C. Wang P. Zhang X. (2021b). Effects of Different Land Use Types on Active Autotrophic Ammonia and Nitrite Oxidizers in Cinnamon Soils. *Appl. Environ. Microbiol.* 87:e92–e21. 10.1128/AEM.00092-21 33837020 PMC8174603

[B335] Wang C. Amon B. Schulz K. Mehdi B. (2021a). Factors that influence nitrous oxide emissions from agricultural soils as well as their representation in simulation models: A review. *Agronomy* 11:770. 10.3390/agronomy11040770

[B336] Wang F. Peng S. B. (2017). Yield potential and nitrogen use efficiency of China’s super rice. *J. Integr. Agric.* 16 1000–1008. 10.1016/S2095-3119(16)61561-7

[B337] Wang F. Chen S. Wang Y. Zhang Y. Hu C. Liu B. (2018). Long-term nitrogen fertilization elevates the activity and abundance of nitrifying and denitrifying microbial communities in an upland soil: Implications for nitrogen loss from intensive agricultural systems. *Front. Microbiol.* 9:2424. 10.3389/FMICB.2018.02424/BIBTEX 30405543 PMC6206047

[B338] Wang L. Xue C. Pan X. Chen F. Liu Y. (2018). Application of controlled-release urea enhances grain yield and nitrogen use efficiency in irrigated rice in the yangtze river basin. China. *Front. Plant Sci.* 9:999. 10.3389/FPLS.2018.00999/BIBTEX 30073007 PMC6060282

[B339] Wang H. Liu Z. Ma L. Li D. Liu K. Huang Q. (2020). Denitrification Potential of Paddy and Upland Soils Derived From the Same Parent Material Respond Differently to Long-Term Fertilization. *Front. Environ. Sci.* 8:105. 10.3389/FENVS.2020.00105/BIBTEX

[B340] Wang Z. Li W. Li H. Zheng W. Guo F. (2020). Phylogenomics of Rhodocyclales and its distribution in wastewater treatment systems. *Sci. Rep.* 10:3883. 10.1038/S41598-020-60723-X 32127605 PMC7054561

[B341] Wang J. Ni L. Song Y. Rhodes G. Li J. Huang Q. (2017). Dynamic response of ammonia-oxidizers to four fertilization regimes across a wheat-rice rotation system. *Front. Microbiol.* 8:630. 10.3389/FMICB.2017.00630/FULL 28446904 PMC5388685

[B342] Wang L. Huang D. (2021). Soil ammonia-oxidizing archaea in a paddy field with different irrigation and fertilization managements. *Sci. Rep.* 11:14563. 10.1038/s41598-021-93898-y 34267287 PMC8282617

[B343] Wang L. Hao D. C. Fan S. Xie H. Bao X. Jia Z. (2022). N2 O Emission and Nitrification/Denitrification Bacterial Communities in Upland Black Soil under Combined Effects of Early and Immediate Moisture. *Agriculture* 12:330. 10.3390/agriculture12030330

[B344] Wang L. Yuan X. Liu C. Li Z. Chen F. Li S. (2019). Soil C and N dynamics and hydrological processes in a maize-wheat rotation field subjected to different tillage and straw management practices. *Agric. Ecosyst. Environ.* 285:106616. 10.1016/J.AGEE.2019.106616 31798199 PMC6743210

[B345] Wassmann R. Jagadish S. V. K. Heuer S. Ismail A. Redona E. Serraj R. (2009). Chapter 2 Climate Change Affecting Rice Production: The Physiological and Agronomic Basis for Possible Adaptation Strategies. *Adv. Agron.* 101 59–122. 10.1016/S0065-2113(08)00802-X

[B346] Wei S. Peng A. Liu J. Wei D. Chen C. (2021). Asymmetric differences in the effects of average air temperature and solar radiation on early rice and late rice yield. *Atmosphere* 12:1541. 10.3390/atmos12121541

[B347] Wessén E. (2011). Niche Differentiation of Ammonia Oxidizing Bacteria and Archaea in Managed Soils. Uppsala: Swedish University of Agricultural Sciences.

[B348] Wick K. Heumesser C. Schmid E. (2012). Groundwater nitrate contamination: Factors and indicators. *J. Environ. Manag.* 111:178. 10.1016/J.JENVMAN.2012.06.030 22906701 PMC3482663

[B349] Wild L. M. Mayer B. Einsiedl F. (2018). Decadal Delays in Groundwater Recovery from Nitrate Contamination Caused by Low O2 Reduction Rates. *Water Resour. Res.* 54 9996–10012. 10.1029/2018WR023396

[B350] Williams S. T. Vail S. Arcand M. M. (2021). Nitrogen use efficiency in parent vs. Hybrid canola under varying nitrogen availabilities. *Plants* 10:2364. 10.3390/PLANTS10112364/S1 34834725 PMC8623409

[B351] Wipf H. M. L. Xu L. Gao C. Spinner H. B. Taylor J. Lemaux P. (2021). Agricultural Soil Management Practices Differentially Shape the Bacterial and Fungal Microbiomes of Sorghum bicolor. *Appl. Environ. Microbiol.* 87:e02345–20. 10.1128/AEM.02345-20 33310712 PMC8090879

[B352] Woli P. Hoogenboom G. (2018). Simulating weather effects on potato yield, nitrate leaching, and profit margin in the US Pacific Northwest. *Agric. Water Manag.* 201 177–187. 10.1016/j.agwat.2018.01.023

[B353] Woodley A. L. Drury C. F. Yang X. Y. Phillips L. A. Reynolds D. W. Calder W. (2020). Ammonia volatilization, nitrous oxide emissions, and corn yields as influenced by nitrogen placement and enhanced efficiency fertilizers. *Soil Sci. Soc. Am. J.* 84 1327–1341. 10.1002/SAJ2.20079

[B354] Wu Y. Xi X. Tang X. Luo D. Gu B. Lam S. K. (2018). Policy distortions, farm size, and the overuse of agricultural chemicals in China. *Proc. Natl. Acad. Sci. U.S.A.* 115 7010–7015. 10.1073/PNAS.1806645115/-/DCSUPPLEMENTAL 29915067 PMC6142251

[B355] Xia L. Lam S. K. Chen D. Wang J. Tang Q. Yan X. (2017). Can knowledge-based N management produce more staple grain with lower greenhouse gas emission and reactive nitrogen pollution? A meta-analysis. *Glob. Chang. Biol.* 23 1917–1925. 10.1111/GCB.13455 27506858

[B356] Xiao G. Zhang Q. Yao Y. Zhao H. Wang R. Bai H. (2008). Impact of recent climatic change on the yield of winter wheat at low and high altitudes in semi-arid northwestern China. *Agric. Ecosyst. Environ.* 127 37–42. 10.1016/J.AGEE.2008.02.007

[B357] Xiong Z.-Q. Huang T.-Q. Ma Y.-C. Xing G.-X. Zhu Z.-L. (2010). Nitrate and Ammonium Leaching in Variable- and Permanent-Charge Paddy Soils. *Pedosphere* 20 209–216. 10.1016/S1002-0160(10)60008-2

[B358] Xu A. Li L. Xie J. Gopalakrishnan S. Zhang R. Luo Z. (2022). Changes in Ammonia-Oxidizing Archaea and Bacterial Communities and Soil Nitrogen Dynamics in Response to Long-Term Nitrogen Fertilization. *Int. J. Environ. Res. Public Health* 19:2732. 10.3390/IJERPH19052732/S1 35270425 PMC8910298

[B359] Xu C. Yang F. Tang X. Lu B. Li Z. Liu Z. (2021). Super Rice With High Sink Activities Has Superior Adaptability to Low Filling Stage Temperature. *Front. Plant Sci.* 12:2111. 10.3389/FPLS.2021.729021/BIBTEX PMC8578116 34777415

[B360] Xu P. Zhang Y. Gong W. Hou X. Kroeze C. Gao W. (2015). An inventory of the emission of ammonia from agricultural fertilizer application in China for 2010 and its high-resolution spatial distribution. *Atmos. Environ.* 115 141–148. 10.1016/J.ATMOSENV.2015.05.020

[B361] Yang Z. Li N. Ma P. Li Y. Zhang R. Song Q. (2020). Improving nitrogen and water use efficiencies of hybrid rice through methodical nitrogen–water distribution management. *F. Crop. Res.* 246:107698. 10.1016/J.FCR.2019.107698

[B362] Yao H. Gao Y. Nicol G. W. Campbell C. D. Prosser J. I. Zhang L. (2011). Links between Ammonia Oxidizer Community Structure, Abundance, and Nitrification Potential in Acidic Soils. *Appl. Environ. Microbiol.* 77:4618. 10.1128/AEM.00136-11 21571885 PMC3127715

[B363] Yao R. J. Li H. Q. Yang J. S. Wang X. P. Xie W. P. Zhang X. (2022). Biochar Addition Inhibits Nitrification by Shifting Community Structure of Ammonia-Oxidizing Microorganisms in Salt-Affected Irrigation-Silting Soil. *Microorganisms* 10:436. 10.3390/MICROORGANISMS10020436/S1 35208890 PMC8878283

[B364] Yao Z. Zheng X. Zhang Y. Liu C. Wang R. Lin S. (2017). Urea deep placement reduces yield-scaled greenhouse gas (CH4 and N2O) and NO emissions from a ground cover rice production system. *Sci. Rep.* 7:11415. 10.1038/S41598-017-11772-2 28900234 PMC5595888

[B365] Ye Y. Liang X. Chen Y. Liu J. Gu J. Guo R. (2013). Alternate wetting and drying irrigation and controlled-release nitrogen fertilizer in late-season rice. Effects on dry matter accumulation, yield, water and nitrogen use. *F. Crop. Res.* 144 212–224. 10.1016/J.FCR.2012.12.003

[B366] Yi J. Gao J. Zhang W. Zhao C. Wang Y. Zhen X. (2019). Differential Uptake and Utilization of Two Forms of Nitrogen in Japonica Rice Cultivars From North-Eastern China. *Front. Plant Sci.* 10:1061. 10.3389/FPLS.2019.01061/BIBTEX 31552066 PMC6738331

[B367] Yoon S. Song B. Phillips R. L. Chang J. Song M. J. (2019). Ecological and physiological implications of nitrogen oxide reduction pathways on greenhouse gas emissions in agroecosystems. *FEMS Microbiol. Ecol* 95:fiz066. 10.1093/FEMSEC/FIZ066 31077302

[B368] Yoshida M. Ishii S. Fujii D. Otsuka S. Senoo K. (2012). Identification of active denitrifiers in rice paddy soil by DNA- and RNA-based analyses. *Microbes Environ.* 27 456–461. 10.1264/jsme2.me12076 22972387 PMC4103554

[B369] Yousefi M. Razdari A. M. (2015). Application of Gis and gps in Precision Agriculture (a Review). *Int. J. Adv. Biol. Biom. Res* 3 7–9.

[B370] Yuan C. L. Zhang L. M. Wang J. T. Hu H. W. Shen J. P. Cao P. (2019). Distributions and environmental drivers of archaea and bacteria in paddy soils. *J. Soils Sediments* 19 23–37. 10.1007/s11368-018-1997-0

[B371] Yuan S. Linquist B. A. Wilson L. T. Cassman K. G. Stuart A. M. Pede V. (2021). Sustainable intensification for a larger global rice bowl. *Nat. Commun.* 12 1–11. 10.1038/s41467-021-27424-z 34887412 PMC8660894

[B372] Yvon-Durocher G. Allen A. P. Bastviken D. Conrad R. Gudasz C. St-Pierre A. (2014). Methane fluxes show consistent temperature dependence across microbial to ecosystem scales. *Nature* 507 488–491. 10.1038/NATURE13164 24670769

[B373] Zanin L. Tomasi N. Wirdnam C. Meier S. Komarova N. Y. Mimmo T. (2014). Isolation and functional characterization of a high affinity urea transporter from roots of Zea mays. *BMC Plant Biol.* 14:222. 10.1186/S12870-014-0222-6/FIGURES/8 25168432 PMC4160556

[B374] Zeng J. Zhao D. Yu Z. Huang R. Wu Q. L. (2014). Temperature Responses of Ammonia-Oxidizing Prokaryotes in Freshwater Sediment Microcosms. *PLoS One* 9:e100653. 10.1371/JOURNAL.PONE.0100653 24959960 PMC4069112

[B375] Zhan X. Shao C. He R. Shi R. (2021). Evolution and efficiency assessment of pesticide and fertiliser inputs to cultivated land in china. *Int. J. Environ. Res. Public Health* 18 3371. 10.3390/IJERPH18073771/S1 33916603 PMC8038477

[B376] Zhang S. Zheng Q. Noll L. Hu Y. Wanek W. (2019b). Environmental effects on soil microbial nitrogen use efficiency are controlled by allocation of organic nitrogen to microbial growth and regulate gross N mineralization. *Soil Biol. Biochem.* 135 304. 10.1016/J.SOILBIO.2019.05.019 31579295 PMC6774787

[B377] Zhang H. Sun H. Zhou S. Bai N. Zheng X. Li S. (2019a). Effect of Straw and Straw Biochar on the Community Structure and Diversity of Ammonia-oxidizing Bacteria and Archaea in Rice-wheat Rotation Ecosystems. *Sci. Reports 2019* 91 1–11. 10.1038/s41598-019-45877-7 31249385 PMC6597706

[B378] Zhang Y. Ding H. Zheng X. Cai Z. Misselbrook T. Carswell A. (2018c). Soil N transformation mechanisms can effectively conserve N in soil under saturated conditions compared to unsaturated conditions in subtropical China. *Biol. Fertil. Soils* 54 495–507. 10.1007/S00374-018-1276-7/FIGURES/7

[B379] Zhang H. Yu C. Kong X. Hou D. Gu J. Liu L. (2018a). Progressive integrative crop managements increase grain yield, nitrogen use efficiency and irrigation water productivity in rice. *F. Crop. Res.* 215 1–11. 10.1016/J.FCR.2017.09.034

[B380] Zhang Y. Liu Y. Zhang G. Guo X. Sun Z. Li T. (2018d). The Effects of Rice Straw and Biochar Applications on the Microbial Community in a Soil with a History of Continuous Tomato Planting History. *Agronomy* 8:65. 10.3390/AGRONOMY8050065

[B381] Zhang J. Zhang N. Liu Y. X. Zhang X. Hu B. Qin Y. (2018b). Root microbiota shift in rice correlates with resident time in the field and developmental stage. *Sci. China Life Sci.* 61 613–621. 10.1007/S11427-018-9284-4 29582350

[B382] Zhang J. B. Wang L. Zhao W. Hu H. F. Feng X. J. Müller C. (2016). Soil gross nitrogen transformations along the Northeast China Transect (NECT) and their response to simulated rainfall events. *Sci. Rep.* 61:22830. 10.1038/srep22830 26949201 PMC4780001

[B383] Zhang J. Feng L. Zou H. Liu D. L. (2015). Using ORYZA2000 to model cold rice yield response to climate change in the Heilongjiang province. *China. Crop J.* 3 317–327. 10.1016/J.CJ.2014.09.005

[B384] Zhang J. Zhang W. Jansson P.-E. Petersen S. O. (2022). Modeling nitrous oxide emissions from agricultural soil incubation experiments using CoupModel. *Biogeosci. Discuss* 2022 1–32.

[B385] Zhang J. Zhu T. Meng T. Zhang Y. Yang J. Yang W. (2013). Agricultural land use affects nitrate production and conservation in humid subtropical soils in China. *Soil Biol. Biochem.* 62 107–114. 10.1016/J.SOILBIO.2013.03.006

[B386] Zhang L.-M. Hu H.-W. Shen J.-P. He J.-Z. (2012). Ammonia-oxidizing archaea have more important role than ammonia-oxidizing bacteria in ammonia oxidation of strongly acidic soils. *ISME J.* 6 1032–1045. 10.1038/ismej.2011.168 22134644 PMC3329103

[B387] Zhang S. Y. Xiao X. Chen S. C. Zhu Y. G. Sun G. X. Konstantinidis K. T. (2021). High Arsenic Levels Increase Activity Rather than Diversity or Abundance of Arsenic Metabolism Genes in Paddy Soils. *Appl. Environ. Microbiol.* 87:e0138321. 34378947 10.1128/AEM.01383-21 PMC8478449

[B388] Zhang Y. Tang Q. Zou Y. Li D. Qin J. Yang S. (2009). Yield potential and radiation use efficiency of “super” hybrid rice grown under subtropical conditions. *F. Crop. Res.* 114 91–98. 10.1016/j.fcr.2009.07.008

[B389] Zhang Z. Xiong S. Wei Y. Meng X. Wang X. Ma X. (2017). The role of glutamine synthetase isozymes in enhancing nitrogen use efficiency of N-efficient winter wheat. *Sci. Rep.* 7:1000. 10.1038/s41598-017-01071-1 28428629 PMC5430530

[B390] Zhao C. Liu B. Piao S. Wang X. Lobell D. B. Huang Y. (2017). Temperature increase reduces global yields of major crops in four independent estimates. *Proc. Natl. Acad. Sci. U.S.A.* 114 9326–9331. 10.1073/pnas.1701762114 28811375 PMC5584412

[B391] Zhao C. Ma G. Zhou L. Zhang S. Su L. Sun X. (2021). Effects of nitrogen levels on gene expression and amino acid metabolism in Welsh onion. *BMC Genomics* 22:803. 10.1186/s12864-021-08130-y 34743697 PMC8573885

[B392] Zhao H. Mo Z. Lin Q. Pan S. Duan M. Tian H. (2020). Relationships between grain yield and agronomic traits of rice in southern China. *Chil. J. Agric. Res.* 80 72–79. 10.4067/s0718-58392020000100072 27315006

[B393] Zhao J. Wang B. Jia Z. (2015). Phylogenetically Distinct Phylotypes Modulate Nitrification in a Paddy Soil. *Appl. Environ. Microbiol.* 81:3218. 10.1128/AEM.00426-15 25724959 PMC4393434

[B394] Zheng Y. Lin X. (2020). Niche Specialization and Functional Overlap of Bamboo Leaf and Root Microbiota. *Front. Microbiol.* 11:2326. 10.3389/FMICB.2020.571159/BIBTEX PMC7531387 33072031

[B395] Zhengchao Z. Zhuoting G. Zhouping S. Fuping Z. (2013). Effects of long-term repeated mineral and organic fertilizer applications on soil organic carbon and total nitrogen in a semi-arid cropland. *Eur. J. Agron.* 45 20–26. 10.1016/J.EJA.2012.11.002

[B396] Zhou Q. Ju C. Wang Z. Zhang H. Liu L. Yang J. (2017). Grain yield and water use efficiency of super rice under soil water deficit and alternate wetting and drying irrigation. *J. Integr. Agric.* 16 1028–1043. 10.1016/S2095-3119(16)61506-X

[B397] Zhou X. Wang J. T. Zhang Z. F. Li W. Chen W. Cai L. (2020). Microbiota in the Rhizosphere and Seed of Rice From China. With Reference to Their Transmission and Biogeography. *Front. Microbiol.* 11:995. 10.3389/FMICB.2020.00995/BIBTEX 32754120 PMC7365946

[B398] Zhu T. Zhang J. Cai Z. Müller C. (2011). The N transformation mechanisms for rapid nitrate accumulation in soils under intensive vegetable cultivation. *J. Soils Sediments* 117 1178–1189. 10.1007/S11368-011-0384-X

[B399] Zhu X. Burger M. Doane T. A. Horwath W. R. (2013). Ammonia oxidation pathways and nitrifier denitrification are significant sources of N2O and NO under low oxygen availability. *Proc. Natl. Acad. Sci.U.S.A.* 110 6328–6333. 10.1073/PNAS.1219993110/SUPPL_FILE/PNAS.201219993SI.PDF 23576736 PMC3631630

[B400] Zhu Z. Bai Y. Lv M. Tian G. Zhang X. Li L. (2020). Soil Fertility, Microbial Biomass, and Microbial Functional Diversity Responses to Four Years Fertilization in an Apple Orchard in North China. *Hortic. Plant J.* 6 223–230. 10.1016/J.HPJ.2020.06.003

[B401] Zistl-Schlingmann M. Kengdo S. K. Kiese R. Dannenmann M. (2020). Management Intensity Controls Nitrogen-Use-Efficiency and Flows in Grasslands—A 15N Tracing Experiment. *Agronomy* 10:606. 10.3390/AGRONOMY10040606

[B402] Zou W. Lang M. Zhang L. Liu B. Chen X. (2022). Ammonia-oxidizing bacteria rather than ammonia-oxidizing archaea dominate nitrification in a nitrogen-fertilized calcareous soil. *Sci. Total Environ.* 811:151402. 10.1016/J.SCITOTENV.2021.151402 34740642

[B403] Zuo J. Hu H. Fu Q. Zhu J. Zheng H. Mo M. (2022). Responses of N2O Production and Abundances of Associated Microorganisms to Soil Profiles and Water Regime in Two Paddy Soils. *Agronomy* 12:743. 10.3390/agronomy12030743

